# Prediction of cognitive performance differences in older age from multimodal neuroimaging data

**DOI:** 10.1007/s11357-023-00831-4

**Published:** 2023-06-13

**Authors:** Camilla Krämer, Johanna Stumme, Lucas da Costa Campos, Paulo Dellani, Christian Rubbert, Julian Caspers, Svenja Caspers, Christiane Jockwitz

**Affiliations:** 1 Institute of Neuroscience and Medicine (INM-1), Research Centre Jülich, Jülich, Germany; 2 Institute for Anatomy I, Medical Faculty & University Hospital Düsseldorf, Heinrich Heine University Düsseldorf, Düsseldorf, Germany; 3 Department of Diagnostic and Interventional Radiology, Medical Faculty & University Hospital Düsseldorf, Heinrich Heine University Düsseldorf, Düsseldorf, Germany

**Keywords:** Cognition, Aging, Machine learning, Multimodal analyses, Graph theoretical approaches

## Abstract

Differences in brain structure and functional and structural network architecture have been found to partly explain cognitive performance differences in older ages. Thus, they may serve as potential markers for these differences. Initial unimodal studies, however, have reported mixed prediction results of selective cognitive variables based on these brain features using machine learning (ML). Thus, the aim of the current study was to investigate the general validity of cognitive performance prediction from imaging data in healthy older adults. In particular, the focus was with examining whether (1) multimodal information, i.e., region-wise grey matter volume (GMV), resting-state functional connectivity (RSFC), and structural connectivity (SC) estimates, may improve predictability of cognitive targets, (2) predictability differences arise for global cognition and distinct cognitive profiles, and (3) results generalize across different ML approaches in 594 healthy older adults (age range: 55–85 years) from the 1000BRAINS study. Prediction potential was examined for each modality and all multimodal combinations, with and without confound (i.e., age, education, and sex) regression across different analytic options, i.e., variations in algorithms, feature sets, and multimodal approaches (i.e., concatenation vs. stacking). Results showed that prediction performance differed considerably between deconfounding strategies. In the absence of demographic confounder control, successful prediction of cognitive performance could be observed across analytic choices. Combination of different modalities tended to marginally improve predictability of cognitive performance compared to single modalities. Importantly, all previously described effects vanished in the strict confounder control condition. Despite a small trend for a multimodal benefit, developing a biomarker for cognitive aging remains challenging.

**Supplementary Information:**

The online version contains supplementary material available at 10.1007/s11357-023-00831-4.

## Introduction

The aging population experiences declines in many cognitive functions, e.g., memory and executive functions [1, 2]. In groups of healthy older adults, age-related cognitive decline has been partly explained by alterations in network architecture, structural (SC) and resting-state functional connectivity (RSFC) of major resting-state networks (RSNs), and grey matter (GM) atrophy [1, 3–13]. However, despite robust findings at the group level, cognitive performance has been found to vary greatly at the individual level [1, 14], particularly in the older ages. In light of the increasing aging population and high relevance of cognitive health for the quality of life of healthy older adults, research has turned to searching for a neuroimaging marker for individual cognitive ability in aging [11, 15–20].

Machine learning (ML) approaches may be particularly appropriate to search for an imaging marker for age-related cognitive decline. This is due to the fact that they may provide information at the individual level and may find patterns in high-dimensional data that might be difficult to capture with univariate methods [21]. Initial ML approaches investigating either resting-state functional connectivity (RSFC), structural connectivity, or grey matter volume (GMV), revealed mixed prediction performance of cognitive measures [15, 18, 19, 22–27]. For instance, by investigating SC, i.e., nodal global and local efficiency, Li et al. could successfully predict attention and executive function in a large sample of healthy older adults (*N* = 633, age range: 45–86 years) [25]. In turn, regional GMV was found to predict fluid reasoning abilities across the adult population (*N* = 335, age range: 20–80 years) in a study by Tsapanou et al. [26], while Hilger et al. revealed decidedly error-prone prediction of intelligence in a large sample of healthy adults (*N* = 308, age range: 18–60 years) [27]. Moreover, recent results from our group emphasize low classifiability and predictability of RSFC strength measures for both, global and domain-specific cognitive abilities, in a large sample of older adults (age range: 55–85 years) [24]. Thus, these partially promising results seem to be rather circumscribed to specific settings, as previous studies all differ in, e.g., their study characteristics, input modalities, and cognitive target variables. To make more general predictions of cognition based on imaging data, however, it may become necessary to directly compare prediction performance across different cognitive variables and input modalities within one sample and the same ML framework.

Furthermore, most previous studies have focused on a single modality in the prediction of cognitive ability in healthy older adults neglecting that brain-behavior relationships arise through the complex interplay between different organizational levels of the brain and its network architecture. Research on neurodegenerative diseases has recently started to integrate information across different modalities in diagnostic classification studies revealing a benefit for multimodal approaches in terms of ML performance [28–30]. For instance, a combination of functionally and structurally derived graph metrics, which may allow to specifically characterize the network architecture of the brain, led to better classification performance in distinguishing patients with mild cognitive impairment (MCI) and Alzheimer’s disease (AD) from healthy controls (HC) [29, 30]. Results from combining multimodal data in healthy older adults and across the lifespan in the prediction of cognitive targets also appear promising [31–33]. For example, Xiao et al. have shown that multimodal imaging models, i.e., amplitude of low-frequency fluctuations (ALFF), fractional anisotropy (FA), and GMV, performed mostly better than unimodal ones in the prediction of visual working memory in a large sample across the lifespan (age range: 18–88 years) [33]. Furthermore, Dadi et al. have demonstrated that fluid intelligence could be predicted from brain volumetric measures, RSFC, and diffusion-derived parameters in a large sample of older adults from the UK Biobank (age range: 40–70 years) [31]. Nevertheless, it remains elusive, if combining information from a functional and structural network perspective, which has already been successfully employed in patient samples, combined with morphologic brain data, i.e., region-wise GMV, may lead to equally promising prediction results especially in higher older ages.

Finally, switching to a methodological perspective, prior studies have shown that prediction accuracies may be affected by the use of different algorithms, feature set sizes, feature selection steps, and deconfounding strategies [34–38]. There is currently no agreement on a standard ML pipeline using neuroimaging data [39] and given the high variability in ML approaches used throughout the field, it may become difficult to compare and discern informational value of each modality for prediction. It, thus, appears warranted to systematically evaluate different analytical choices and their impact on prediction performance.

The current study, hence, aimed at examining the general validity of the prediction of cognitive performance from imaging data in healthy older adults. Particularly, it was directed at investigating whether (1) combining information from a network perspective, i.e., RSFC and SC estimates, with morphological brain data, i.e., region-wise GMV, may lead to better predictability of different cognitive targets than unimodal models, (2) differences emerge in the prediction of global cognition and distinct cognitive profiles, and (3) results generalize across different ML pipeline configurations and approaches, i.e., different modality combinations, algorithms, feature sets, deconfounding analyses, and multimodal approaches, in a large sample of healthy older adults from the 1000BRAINS study.

## Methods

### Participants

Data for the current analyses was derived from the 1000BRAINS study [40], which aims at investigating age-related variability in brain structure and function in light of environmental, behavioural and genetic factors in an epidemiologic population-based design. The 10-year follow-up cohort of the Heinz Nixdorf Recall Study and the MultiGeneration Study was used to define the 1000BRAINS sample [41]. A total of 966 participants of the whole sample met the age criteria of the current study (age range: 55–85 years). Missing resting-state functional magnetic resonance imaging (fMRI), structural magnetic resonance imaging (sMRI), or diffusion-weighted imaging (DWI) data or failed preprocessing of functional and structural imaging data led to the exclusion of 248 participants from the initial sample. In a next step, 95 participants were excluded as preprocessed data did not meet quality standards described in more detail below. Further, 27 participants with missing values on the dementia screening test DemTect or scoring ≤ 8 were excluded in light of potential cognitive impairment [42]. More than three missing values in the neuropsychological assessment led to the exclusion of additional 2 participants. A final sample of 594 participants (296 females, *M*_age_ = 66.88 years, SD_age_ = 6.67, see Table 1) was used for further analyses. The study protocol of 1000BRAINS was approved by the Ethics Committee of the University of Essen, Germany, and all subjects provided written consent prior to inclusion.

**Table 1:** Demographic information of sample regarding age, educational level and risk of dementia

	*N*	Age (in years)	Education (measured by ISCED)	DemTect score
Female	296	66.26 (6.44)	5.99 (1.83)	15.55 (2.25)
Male	298	67.50 (6.84)	7.03 (1.91)	14.41 (2.34)
Total	594	66.88 (6.67)	6.51 (1.94)	14.98 (2.36)

Mean displayed with standard deviation (SD) appearing in parentheses

### Functional and structural brain data

Functional and structural imaging data was acquired on a 3T Siemens Tim-TRIO MR scanner with a 32-channel head coil. A 3D high-resolution T1-weighted magnetization prepared rapid acquisition gradient-echo (MPRAGE) sequence was obtained for subsequent surface reconstruction and brain structural analyses (176 slices, slice thickness = 1 mm, TR = 2250 ms, TE = 3.03 ms, FoV = 256 × 256 mm^2^, flip angle = 9°, voxel resolution = 1 × 1 × 1 mm^3^). Resting-state fMRI was acquired for about 11 min resulting in 300 EPI (gradient-echo planar imaging) volumes (slices = 36, slice thickness = 3.1 mm, TR = 2200 ms, TE = 30 ms, FoV = 200 × 200 mm^2^, voxel resolution = 3.1 × 3.1 × 3.1 mm^3^). During the resting-state scan, participants were asked to keep their eyes closed, to relax and let their mind wander, but not to fall asleep. A post-scan debriefing was used as a check. Additionally, high-angular resolution diffusion imaging (HARDI) data was obtained using the following parameters: (i) 60 direction subset; EPI, TR = 6300 ms, TE = 81 ms, 7 b0-images (interleaved), 60 images with b = 1000 s/mm^2^, voxel resolution = 2.4 × 2.4 × 2.4 mm^3^; (ii) 120 direction subset; EPI, TR = 8000 ms, TE = 112 ms, 13 b0-images (interleaved), 120 images with b = 2700 s/mm^2^, voxel resolution = 2.4 × 2.4 × 2.4mm^3^.

### Image preprocessing

**Fig. 1:**
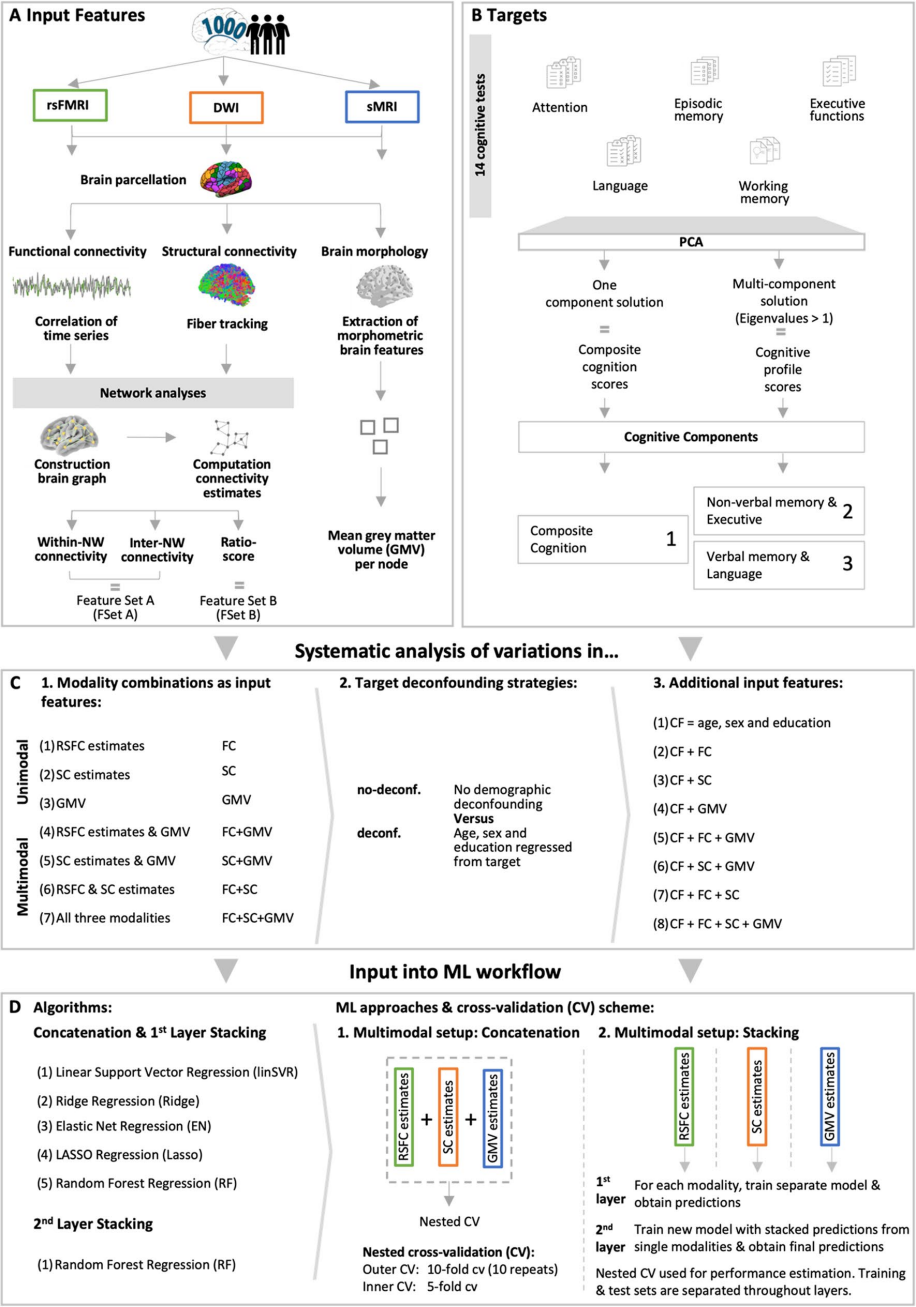
Schematic overview of workflow

The T1-weighted 3D anatomical images were preprocessed using the “recon-all” automated cortical reconstruction pipeline of the FreeSurfer 7.1.0 Software package [43] as described under http://surfer.nmr.mgh.harvard.edu. The original pipeline includes a range of brain parcellations derived from cortical surface models constructed from manually or automated labelled training sets. We adapted the original pipeline to also include the 400-node Schaefer parcellation, which is based on cortical surface models calculated from rsfMRI measurements of 1489 participants using a gradient weighted Markov random field approach [44]. First, the parcellation was transformed to individual subject space using FreeSurfer’s mris_ca_label tool. Then, morphology values were gathered for every transformed node using FreeSurfer's mris_anatomical_stats tool. Afterwards measures, such as surface area, grey matter volume (GMV), and cortical thickness of every node for the left and right brain hemisphere, were summarized in separate tables using FreeSurfer’s aparcstats2table utility. The GMV values for each node (= 400) were used as features in the ML pipeline (see Fig. 1: Features). To ensure data quality, mean GMV values were calculated and participants with values greater than 1.5 times the inter-quartile range were excluded from further analyses.

Functional and diffusion tensor images were preprocessed according to an established pipeline by [12]. For all functional images, this included (1) deletion of the first four EPI volumes, (2) head movement correction using a two-pass procedure, (2) application of ICA-based Automatic Removal of Motion Artifacts (ICA-AROMA) [45] combined with global signal regression, (3) application of a band-pass filter (0.01–0.1 Hz), and (4) registration to MNI152 template using a unified segmentation approach [46]. An additional quality check for the preprocessing of functional images was carried out according to [12], which included (1) checking for potential misalignments in the mean functional AROMA data with the check sample homogeneity option in the Computational Anatomy Toolbox (CAT12) [47] (participants identified as outliers with > 2 SD away from the mean excluded) and (2) checking for volume-wise severe intensity dropouts (DVARS) in the preprocessed data using an algorithm by [48] (participants with more than 10% of the 300 volumes detected as dropouts excluded).

Diffusion image processing involved (1) calculation of tissue probability maps (TPM) for grey matter (GM), white matter (WM), and cerebrospinal fluid (CSF) from T1 data using CAT12 toolbox [47], (2) extraction of brain from T1 data by using brain masks created by superimposing the three probability maps, (3) bias field correction of T1 data, rigid alignment to the MNI152 template and resampling to 1.25 mm isotropic voxel size, (4) correction of dMRI data for eddy currents and motion artefacts, (5) visual quality control to remove remaining noisy data, (6) alignment of dMRI data to individual T1 space, (7) computation of anisotropic power maps (APMs) from b2700 dMRI data for image registration, (8) transformation of TPMs to diffusion space via APMs, (9) merging of the two dMRI datasets (b1000 & b2700) into one, (10) computation of the constrained spherical deconvolution (CSD) model using multi-tissue CSD with multi-shell data [49], and (11) application of probabilistic streamline tractography and computation of 10 million streamlines with dynamic seeding at the grey-white matter interface using the iFOD2 algorithm (max. length = 250 mm; cut-off value = 0.06).

### Functional and structural connectivity analyses

For connectivity analyses, the same protocol as in [12] was followed. The brain was parcellated into 400 cortical parcels according to [44], which were assigned to seven known resting-state networks (visual, sensorimotor, limbic, frontoparietal, default mode, dorsal, and ventral attention network) [50]. Each parcel served as nodes in the subsequent graph-theoretical analysis.

For both functional and structural connectivity, a 400 × 400 adjacency matrix for each participant was obtained. For functional data, each matrix entry reflected the Pearson’s correlation of the average time series of two nodes. As an additional step, a statistical significant test of each correlation coefficient was performed making use of the Fourier transform and permutation testing (1000 repeats) to reduce the amount of spurious correlations [11, 12, 51]. Non-significant edges at *p* ≥ 0.05 were set to zero. Afterwards, a Fisher’s r-to-z-transformation was used to transform the 400 × 400 adjacency matrix. In subsequent analyses only positive correlations were considered and no further thresholding in terms of network size and network density was applied to the brain graph. Thus, a positively weighted network was used for the computation of connectivity estimates. For diffusion data, each matrix entry constituted a weighting factor derived from streamline counts between each pair of nodes using a cross-sectional area multiplier (SIFT-2) [52]. Before obtaining each matrix entry, the following steps were performed: (1) warping of the parcellation template to individual diffusion space using the combination of nonlinear warps of spatial T1 registration to MNI152 template and distortion correction with APMs, (2) expansion of template by adding voxels towards the grey-white matter boundary for seeding points to be included in regions. Ultimately, the diffusion matrix was log10 transformed.

In a final step, connectivity estimates were calculated from both functional and structural connectome data using the software *bctpy* with network parameters defined as in [53] (https://pypi.org/project/bctpy/) (see Fig. 1A). For both RSFC and SC, the focus was with nodal-level (1) within-network connectivity (400 features) defined as the sum of weights of one node attached to all nodes within its respective network divided by the total number of edges in the network, (2) inter-network connectivity (400 features) defined as the sum of weights from one node to all nodes outside its respective network divided by the number of edges in the network as well as (3) a ratio score (400 features) defined as within-network connectivity of a node in relation to its inter-network connectivity [12]. The total feature vector for each participant encompassed 2,800 features (1200 RSFC estimates + 1200 SC estimates + 400 region-wise GMV values). Two different feature sets were obtained from this and used in the ML framework explained below (Feature Set (FSet) A: 2 × 400 within- & inter-network connectivity for FC & SC + 400 region-wise GMV = 2000 features; Feature Set (FSet) B: 2 × 400 ratio-score for FC & SC + 400 region-wise GMV = 1200 features; see Fig. 1A).

### Cognitive performance

All subjects took part in extensive neuropsychological assessment. For the current analyses, 14 cognitive tests spanning the cognitive domains attention, executive functions, episodic memory, working memory (WM) and language were selected (for details regarding test and variables chosen, see Suppl. Table S1) [40]. Due to the differential impact of aging on specific cognitive functions, we were interested in the examination of both global cognition and specific cognitive profiles in the prediction setting [1]. Therefore, we derived composite cognition scores following [24]. In summary, this included (1) replacement of missing values by the median for respective sex (males, females) and age groups (55 − 64 years, 65 − 74 years, 75 − 85 years), (2) conversion of raw scores into *z*-scores, (3) inversion of test scores with higher values meaning lower performance (i.e., time to complete the tasks or number of errors made), and (4) reduction of test performance to a global composite (one component solution) and distinct cognitive profiles (multicomponent solution based on eigenvalues > 1) using principal component analysis (PCA). Targets in ML prediction of cognitive performance constituted the individual global component and cognitive profile scores extracted from the PCA (see Fig. 1B). All cognitive analyses were performed using IBM SPSS Statistics 26 (https://www.ibm.com/de-de/analytics/spss-statistics-software) and custom Python (Version 3.7.6) code.

### Machine learning framework

To answer the main question of this study, whether cognitive performance in healthy older adults can be predicted more accurately by multimodal information (region-wise GMV, RSFC & SC estimates) than by single modalities, a comprehensive ML framework approach was chosen. A schematic overview of the workflow can be found in Fig. 1D. Previous studies have shown that the use of a stacking approach in a multimodal context may be beneficial for prediction performance [54, 55]. To systematically examine a potential additional benefit of stacking for prediction accuracy, multimodal analyses were carried out both in a concatenation and stacking approach. In the concatenation approach, feature vectors in the multimodal settings were simply concatenated into one feature vector and entered into the ML pipeline. In contrast, stacking refers to an ensemble learning paradigm, which comprises two levels of learning [54, 55]. In the first layer, a machine learning (ML) model is obtained from each modality separately and each modality is in turn used to predict the cognitive variable of interest. The cross-validated predictions from the single-modality models are then used as the new feature vector for the second layer. In the second layer, the new input vector is used to train a meta-estimator and used for final predictions.

ML estimations were obtained for all single modalities, for pairwise combinations, and for a three-way combination (see Fig. 1C: Modality combinations as input features). Performance of different prediction algorithms were compared, which have been frequently applied in similar settings [32, 54–58]. These included Ridge regression, linear Support Vector Regression (linSVR), LASSO regression, Elastic Net (EN) regression, and Random Forest (RF) regression [32, 54–56, 59] (see Fig. 1D: Algorithms). The different algorithms were used in concatenation and in the first layer of the stacking approach. As the meta-estimator in stacking, a RF regressor was implemented according to recommendations in the literature [54–56, 58, 60, 61].

Following [62], ML model performance was evaluated using a repeated nested 10-fold cross-validation with 10 repeats (see Fig. 1D: ML approaches & cross-validation (CV) scheme). All hyperparameters were optimized in the inner folds to avoid data leakage (5-fold CV). In an initial step of the ML pipeline, all input features were scaled using the StandardScaler from scikit-learn within the cross-validation setup to ensure comparability in magnitudes of input features. In stacking, splits into training and test sets for single modalities were retained for training the second layer meta-estimator, i.e., RF regressor, to ensure separation of training and test set across layers and avoid data leakage [62]. To obtain the new input data for the second layer for each modality, predictions in the training set were obtained for each iteration of the repeated 10-fold CV based on the optimal hyperparameter configuration determined by an inner 5-fold CV. Those cross-validation predictions were then stacked for each iteration of the outer CV cycle and used as the new training set for the second layer. In turn, predictions on the test set for each iteration of the repeated tenfold CV were obtained, stacked and used as the new test set for the second layer. This procedure was performed to ensure that throughout all layers the training and test set were kept separate and that final stacked models were tested on previously unseen predictions [62]. Hyperparameters, i.e., number of trees and tree depth, of the meta-estimator were optimized in inner folds. The best parameter combination in terms of inner fold performance (i.e., MAE) was selected, applied to the outer fold training set and tested on the outer test set to evaluate ML performance. The following hyperparameters were tuned in both the concatenation and stacking approach: (i) regularization parameter C for linSVR (C: 10^−4^ to 10^1^, 10 steps, logarithmic scale), (ii) regularization parameter lambda $\lambda$ for Lasso ($\lambda$: 10^−1^ to 10^2^, 10 steps) and Ridge ($\lambda$:10^−3^ to 10^5^, 10 steps, logarithmic scale), (iii) regularization parameter lambda,$\lambda$, and alpha,$\alpha$, for EN ($\lambda$: 10^−1^ to 10^2^, 10 steps, logarithmic scale;$\alpha$: 0.1 to 1, 10 steps), and (iv) number of trees and tree depth for RF (number of trees: 100 or 1000; tree depth: 4, 6, 8, 10, 20, 40, None). Mean absolute error (MAE) and coefficient of determination (*R*^2^) were used to assess prediction performance. For completeness, the Pearson’s correlation (*r*) between true and predicted targets was also calculated and reported in the Supplement. All machine learning analyses were performed using the scikit-learn library (version: 0.22.1) in Python [63] (https://scikit-learn.org/stable/index.html). Scripts for stacking were based on those from [62] (https://github.com/axifra/BrainAge_MRI-MEG) and adapted for the current study.

### Confounder analyses

As ML performance may be extensively impacted by confounding variables, two different confounder analyses were carried out in the current study. First, we investigated prediction performance in conditions with different extents of deconfounding, i.e., without (no-deconf. condition) and with (deconf. condition) demographic confound regression (see Fig. 1C: Deconfounding). In both conditions, we controlled for the influence of estimated total intracranial volume (eTIV) by regressing it from the target [27, 55, 64]. In the deconf. condition, we additionally controlled for the demographic variables age, sex, and educational level in a similar fashion [55]. Confound regression was always performed within the ML pipeline to avoid data leakage [24, 55]. Second, prediction performance was examined in models using age, sex, and educational level as extra features (see Fig. 1C: Additional input features) [55]. ML estimations were obtained for demographic variables only and for all combinations with brain features.

### Feature importance

Feature importance information was derived at two levels, i.e., feature and modality level, in the current study. For a more fine-grained anatomical exploration of the most relevant features (i.e., feature level), we decided to investigate results from the concatenation approach. To identify important features, mean coefficients were calculated by averaging coefficients across all CV folds for each ML model. For complexity reduction, we focused on the concatenation approach in the no-deconf. condition and models, in which all features were combined, to extract relevant features for prediction. The analyses of meaningful features were separately performed for models without and with extra features to gain a greater insight into the relevance of demographic features and the added benefit of using brain features for prediction. In an initial step, the 20 features with the highest coefficients were selected for each target in each algorithm (i.e., linSVR, Ridge, EN, Lasso, RF) and feature set (FSet A & FSet B). To ensure that features were consistently highly ranked across different analytic choices, only those features present in all algorithms and feature sets for each target were kept. Then, centroid coordinates of selected nodes in MNI space were retrieved from the 400-node Schaefer parcellation. Ultimately, an anatomical label using the cytoarchitectonically defined Julich-Brain atlas [65] implemented in the EBRAINS multilevel atlas framework (https://ebrains.eu/) was provided. In cases, in which a node was found within a gap map, the Desikan-Killiany atlas [66] implemented in FreeSurfer’s freeview was additionally used.

For the closer examination at the modality level, feature importance information was derived from the second layer, i.e., meta-learner RF, of the stacking approach. Mean feature importances for each modality were calculated in the same way as in the feature level analysis. Again, to reduce complexity, focus was with the no-deconf. condition and models, in which all modalities, i.e., FC + SC + GMV, were combined. Feature importance analyses were performed for models without and with extra features. Each modality was ranked based on the feature importance results across analytic choices for each cognitive target. The most common ranking was reported in the Supplement.

### ML validation analyses

We performed further analyses to validate our ML approach. Firstly, prediction performance was assessed for a theoretically defined composite (global) cognitive score to evaluate whether similar results are achieved as in our data-driven approach. To obtain a theoretically defined composite cognition score, test performance on the 14 cognitive tests (i.e., Z-scores) was averaged for each individual and used as targets in ML. Additionally, we chose to validate our findings by classifying extreme cognitive groups using a linear Support Vector Classifier (linSVC), Logistic Regression (Log), Ridge and Random Forest (RF) classifier. Extreme groups were defined as the top 25% (high cognitive performers) and lowest 25% (low cognitive performers) of individuals scoring on the global cognition component [31, 32]. Groups were matched for age, educational level, sex, and eTIV using propensity score matching (*N* = 116, 56 females, *M*_age_ = 65.89, SD_age_ = 6.06; see Suppl. Table S3-4). Moreover, we investigated the impact of including RSFC estimates derived from negative correlations on prediction performance exemplary for global cognition in the concatenation approach across analytic choices (FSet C: 2 × 400 within- & inter-network connectivity for positive FC, 2 × 400 within- & inter-network connectivity for negative FC, 2 × 400 within- & inter-network connectivity for SC + 400 region-wise GMV = 2800 features). To validate our ML pipeline and to gain a greater insight into the confounding variables, we also performed age, educational level, and sex (matched for age, education & eTIV; *N* = 340, 170 females, *M*_age_ = 66.57, SD_age_ = 6.77; see Suppl. Table S2) predictions.

### Model comparison and statistical analyses

Partial correlations between cognitive scores and age (corrected for education and sex) as well as education (corrected for age and sex) were computed to examine the link between potential confounders and cognitive performance, as summarised by the components derived from the PCA. A multivariate analysis of covariance (MANCOVA) was calculated to examine sex differences in cognitive variables (DV = cognitive scores, IV = sex, covariates = age and education).

ML performance was compared to estimations from a reference model, i.e., Dummy regressor [56]. In this case, the percentage of folds, for which the ML models were better than the reference model, was calculated. Further, two different types of multimodal bonus, B_all_ and B_best_, were calculated for each multimodal combination according to [55]. B_all_ reflects the difference in performance between each multimodal model and the average of single modalities, while B_best_ constitutes the difference in performance between the multimodal model and the best single modality.

## Results

### Cognitive composite scores derived from principal component analysis

**Fig. 2:**
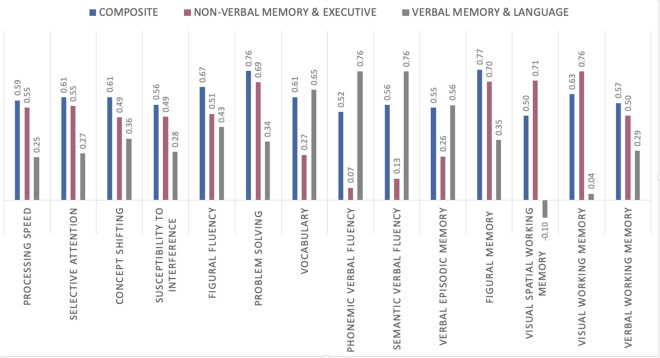
Factor loadings of each cognitive function on the one component and multicomponent solution extracted from PCA analysis (after Varimax rotation)

Principal component analysis (PCA) was used to derive cognitive composite scores, i.e., global cognition and specific cognitive profiles. First, the Kaiser-Meyer-Olkin (KMO) index was used to assess data suitability for PCA. The index was found to be satisfactory with a value of 0.91. Cognitive composite scores for each participant were defined as component scores derived from a one component solution. Cognitive profile scores for each individual were extracted from a solution based on the eigenvalue criterion > 1. In this context, two components could be identified by PCA (see Suppl. Tables S5-6 & Suppl. Fig. S7). The first component mostly related to (working) memory and executive functions, i.e., visual, visual spatial, and verbal WM, figural memory, problem solving, concept shifting, and susceptibility to interference (non-verbal memory & executive component; see Fig. 2 & Suppl. Table S6). The second component primarily pertained to verbal memory and language functions, i.e., semantic and phonemic verbal fluency, vocabulary, and verbal episodic memory (verbal memory & language component; see Fig. 2 & Suppl. Table S6).

All three cognitive scores were significantly negatively associated with age (cognitive composite: *r* =  − 0.45, *p* < 0.001, non-verbal memory & executive: *r* =  − 0.41, *p* < 0.001, verbal memory & language: *r* =  − 0.16, *p* < 0.001; adjusted for educational level and sex). Higher performance in all cognitive scores was significantly correlated with higher educational level (cognitive composite: *r* = 0.43, *p* < 0.001, non-verbal memory & executive: *r* = 0.21, *p* < 0.001, verbal memory & language: *r* = 0.39, *p* < 0.001). No sex differences were found for the global composite cognitive score using a MANCOVA with age and education as covariates (cognitive composite: *F*(1,590) = 0.83,* p* = 0.36, *η*_p_^2^ = 0.001). However, significant performance differences between males and females emerged for the two cognitive profiles (memory & executive: *F*(1,590) = 16.52, *p* < 0.001, *η*_p_^2^ = 0.03; verbal memory & language:* F*(1,590) = 43.04,* p* < 0.001, *η*_p_^2^ = 0.07).

### ML results

#### Prediction results from unimodal and multimodal brain features for global cognition

**Fig. 3:**
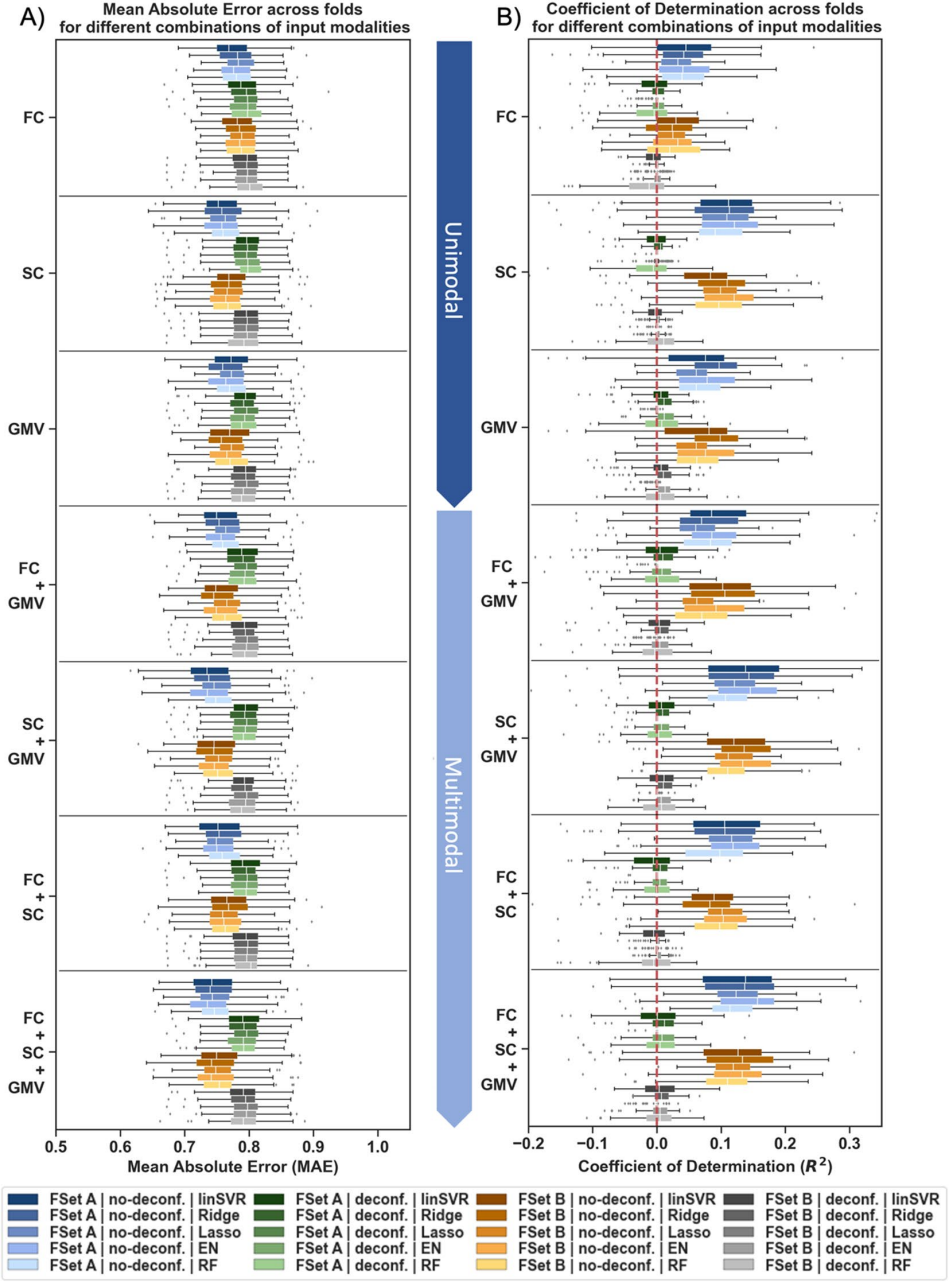
Prediction performance for global cognition using unimodal and multimodal data across feature sets (FSet) A and B in the concatenation approach. **A** Mean absolute error (MAE) and **B** coefficient of determination (*R*^2^) shown across folds for different algorithms (linear Support Vector Regression (linSVR), Ridge, Lasso, Elastic Net (EN) and Random Forest (RF) regression) and deconfounding strategies (no-deconf. = no deconfounding except for controlling for eTIV in target, deconf. = confound regression of age, sex, education, & eTIV)

**Fig. 4:**
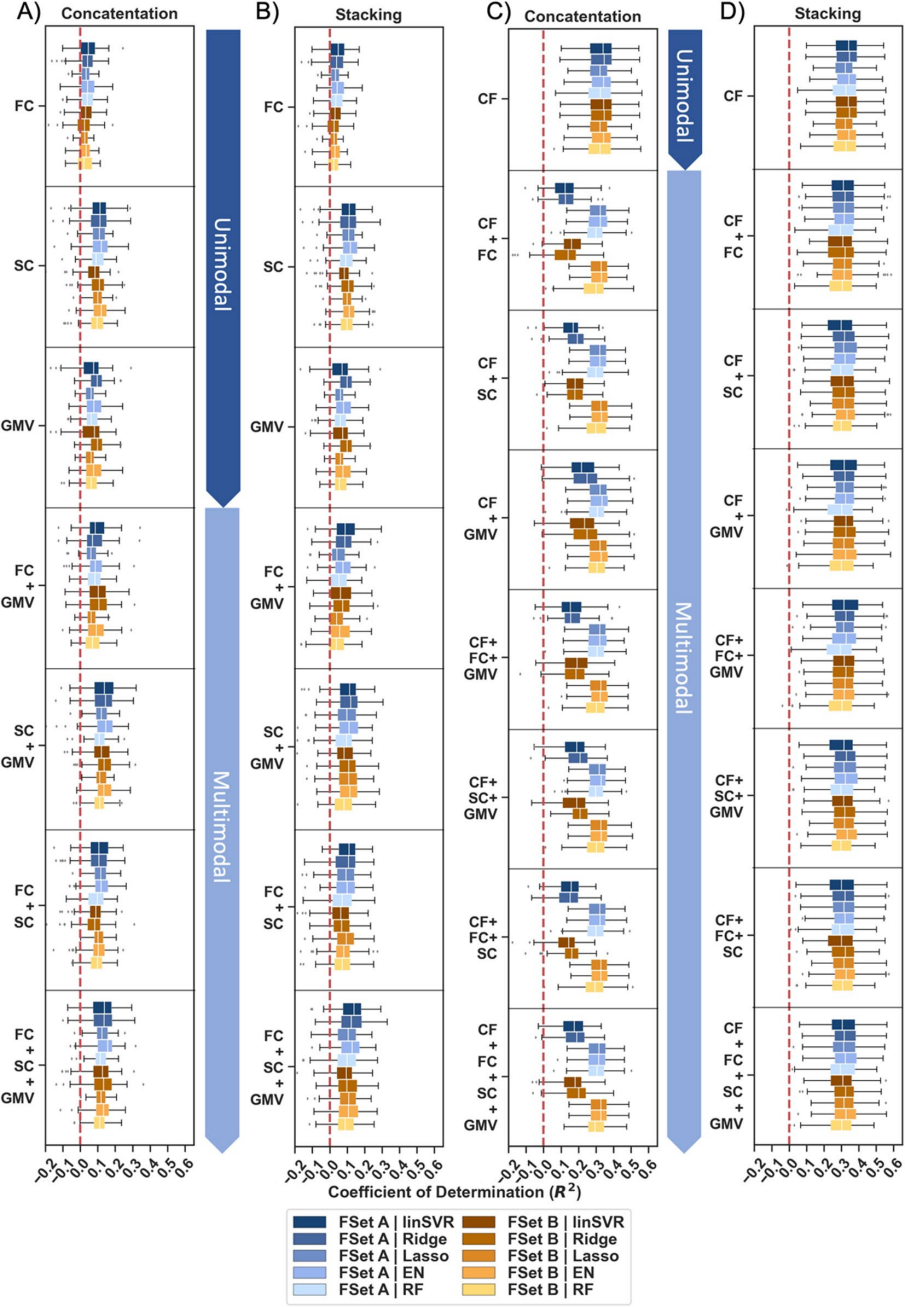
Prediction performance for global cognition using unimodal and multimodal data across feature sets (FSet) **A** and **B **in the concatenation and stacking approach with and without extra features. Coefficient of determination (*R*^2^) displayed across folds for different algorithms (linear Support Vector Regression (linSVR), Ridge, Lasso, Elastic Net (EN) and Random Forest (RF) regression). Results shown for **A** the concatenation approach without extra features, **B** the stacking approach without extra features, **C** the concatenation approach with extra features, **D** the stacking approach with extra features. Only no-deconf. condition shown

Initially, ML was used to assess the prediction power of multimodal brain features, i.e., region-wise GMV, RSFC, and SC estimates, for global cognitive performance in older adults. Prediction performance across algorithms, feature sets, and ML approaches differed greatly between deconfounding strategies. Satisfactory prediction performance was only observed when no deconfounding was applied (Mean MAE: 0.74–0.79, Mean *R*^2^: 0.02–0.14, in 65–100% of folds *R*^*2*^ > dummy regressor; see Suppl. Tables S8-9, 11–16 & Suppl. Fig. S10). In this setting, multimodal models (Mean MAE: 0.74–0.78, Mean *R*^2^: 0.03–0.14) tended to slightly better predict global cognitive performance than unimodal models (Mean MAE: 0.75–0.79, Mean *R*^2^: 0.02–0.11) in different approaches, feature sets, and algorithms (see Figs. 3, 4 & Suppl. Tables S8-9, 11–16 & Suppl. Fig. S10). Across cognitive domains, a prediction performance gain in the best cases of up to 0.04 (best unimodal, B_best_) to 0.06 (average unimodal, B_all_) in *R*^2^ could be observed in multimodal compared to unimodal models (see Suppl. Tables S17-20). Among single modalities, RSFC estimates (Mean MAE: 0.77–0.79, Mean *R*^2^: 0.02–0.04) were found to be least predictive of global cognition across analytic choices (SC & GMV: Mean MAE: 0.75–0.78, Mean *R*^2^: 0.05–0.11; see Figs. 3, 4 & Suppl. Tables S8-9, 11–16 Suppl. Fig. S10). Once we controlled for age, sex, and education, global cognition could no longer be successfully predicted and all previously reported differences between modalities disappeared (Mean MAE: 0.79–0.80, Mean *R*^2^: –0.04–0.01, in 3–77% of folds *R*^2^ > dummy regressor; Suppl. Tables S8-9, 11–16 & Suppl. Fig. S10). Thus, successful prediction of global cognition based on structural as well as structural and functional connectivity neuroimaging features along with a tendency for a multimodal benefit was only found in absence of confounder control.

#### Prediction results for global cognition using demographic features, i.e., age, sex, and education, and brain features plus extra demographic features

To get a better understanding of the impact of demographic feature on the cognitive performance prediction, prediction performance for global cognition was then investigated for models using only demographic features and models using brain features plus demographic features in absence of confounder control. Across approaches, algorithms, and feature sets, models including demographic features (i.e., age, sex, and education) could predict global cognition to a much greater degree than models solely based on brain features (Without extra features: Mean MAE: 0.74–0.79, Mean *R*^2^: − 0.02–0.14, in 65–100% of folds *R*^2^ > dummy regressor; With extra features: Mean MAE: 0.64–0.75, Mean *R*^2^: 0.12–0.34, i.e., in 92–100% of folds *R*^2^ > dummy regressor; see Fig. 4 & Suppl. Tables S21-24). Numerically, models with extra features could explain up to 20% more variance (*R*^2^) in global cognition compared to those without. Importantly, it should be highlighted that solely demographic features (Mean MAE: 0.64–0.65, Mean *R*^2^: 0.32–0.34, in 100% of folds *R*^2^ > dummy regressor) predicted global cognition to a similar or even higher extent than brain features combined with demographic features (Mean MAE: 0.64–0.75, Mean *R*^2^: 0.12–0.33; see Fig. 4 & Suppl. Tables S21-24). Thus, demographic information, i.e., age, sex, and education, were found to be highly predictive of global cognitive performance in older subjects (once these are not strictly controlled for by confound regression).

#### Prediction results for global cognition in the concatenation and stacking approach

As previous studies have reported a benefit of stacking in terms of prediction accuracy, ML performance for global cognition was compared between a concatenation and stacking approach. In the current study, global cognition was predicted to a similar extent in the stacking (Mean MAE: 0.64–0.81, Mean *R*^2^: − 0.03–0.34) and the concatenation (Mean MAE: 0.64–0.80, Mean *R*^2^: − 0.04–0.34) approach (see Fig. 4 & Suppl. Tables S8-9, 11–16, 21–24). Only in models with extra features, differences between approaches emerged for two algorithms, i.e., linSVR and Ridge regression. Here, the prediction behaviour was found to be more stable in the stacking approach (see Fig. 4B, D). Nonetheless, the overall benefit of using a stacking approach remained marginal in the current investigation. Results for the two specific cognitive profiles are reported in the Supplement (see Suppl. Tables S25-48) and follow a similar pattern as global cognition.

#### Prediction results for global cognition and specific cognitive profiles

**Fig. 5:**
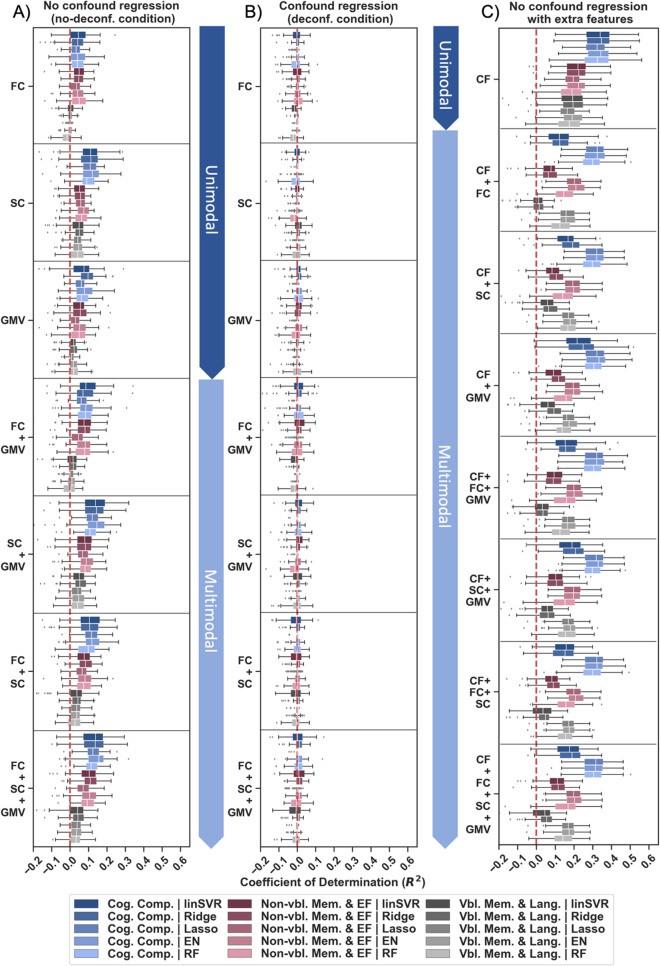
Prediction performance for global cognition (Cog. Comp.) and specific cognitive profiles (Non-vbl. Mem. & EF, Vbl. Mem., & Lang.) using unimodal and multimodal data in feature set (FSet) **A **in the concatenation approach with and without extra features. Coefficient of Determination (*R*^2^) displayed across folds for different algorithms (linear Support Vector Regression (linSVR), Ridge, Lasso, Elastic Net (EN) and Random Forest (RF) regression). Results shown for **A** no-deconf. condition without extra features, **B** deconf. condition without extra features, **C** no-deconf. condition with extra features

To address potential predictability differences across cognitive domains, prediction performance was further considered separately for global cognition and distinct cognitive profiles. Results revealed that global cognition and the two cognitive profiles may be predicted to different extents in absence of confounder control. Across modalities, pipeline configurations and algorithms, multimodal imaging data best predicted global cognition (Mean MAE: 0.74–0.79, Mean *R*^2^: − 0.04–0.14) followed by the non-verbal memory & executive functions component (Mean MAE: 0.74–0.78, Mean *R*^2^: − 0.03–0.11) and the verbal memory & language component (Mean MAE: 0.79–0.82, Mean *R*^2^: − 0.03–0.05; see Fig. 5A & Suppl. Tables S8-9, 11–16, 21–48). It should be emphasized that while ML models could explain at least a moderate amount of variance in both global cognition and the non-verbal memory & executive functions component, this was not the case for the verbal memory & language component (see Fig. 5A). Despite an overall increase in prediction performance, predictability differences between targets were also found in models with extra features and disappeared altogether, when we controlled for age, sex, and education (see Fig. 5B, C). Hence, results hint at considerably lower predictability of language functions in older age based on currently employed multimodal input features.

#### Relevant features for the prediction of cognitive performance in older age

**Fig. 6:**
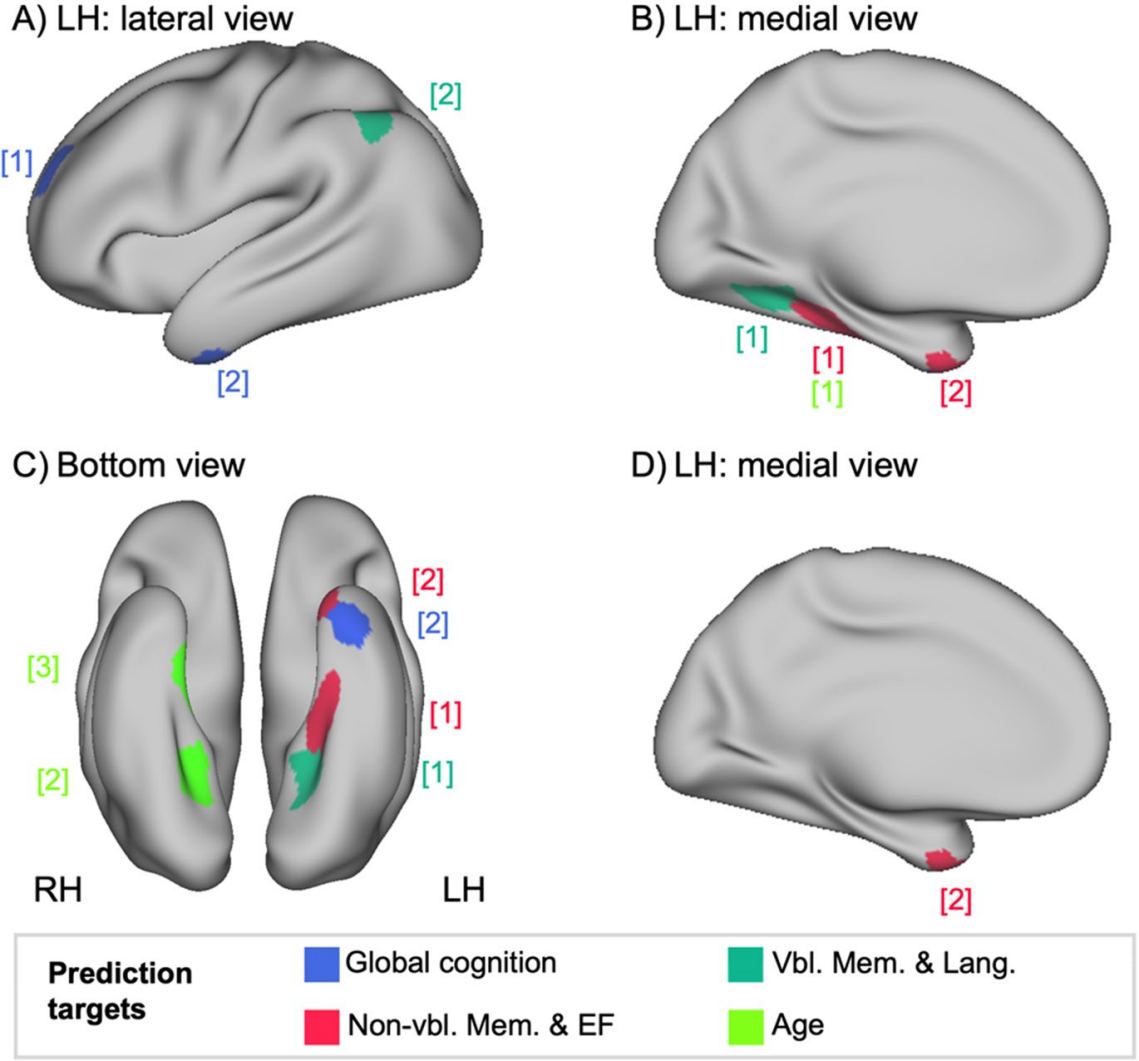
Mapping of relevant features for the prediction of cognitive performance in older age to brain.** A**–**C** Nodes (in different colors labelled for different targets) relevant for prediction with no extra features, **D** node relevant for prediction with extra features.

The analyses of important features were performed at both feature and modality level. In the feature level approach, analyses were separately carried out for models with and without extra features for the different cognitive targets and age in the concatenation approach. Across models without extra features, top ranked features for prediction of cognitive targets either belonged to the modality SC or GMV. In case of SC, inter-network connectivity features were more frequently found among the top ranked features than within-network features (see Fig. 6 & Table 2). For global cognition, nodes found in the rostral middle frontal gyrus (GMV; DMN) and the inferior temporal/parahippocampal gyrus (SC; limbic network) were found to be important (see Fig. 6 & Table 2). In turn for the non-verbal memory & executive functions component, nodes in the parahippocampal / fusiform gyrus (SC; visual network) and temporal pole / entorhinal cortex (SC; limbic network) were relevant for prediction. For the verbal memory & language component, relevant nodes were found in the lingual / fusiform / parahippocampal gyrus (SC; visual network) and the angular gyrus (GMV; DMN) (see Fig. 6 & Table 2). For the age prediction, important nodes were found in the left and right parahippocampal gyrus (SC; visual and limbic network) and right fusiform / lingual gyrus (SC; visual network). Overlap was encountered in one feature with the non-verbal memory & executive functions component (see Fig. 6 & Table 2). In contrast, in models with extra features, the most relevant features constituted the demographic extra features and nearly no brain features reappeared among the top ranked features (see Table 2). For global cognition and the non-verbal memory & executive functions component, age and education were now found to be the most important features for prediction. A node in the temporal pole/entorhinal cortex (SC; limbic network) was additionally relevant for the prediction of the non-verbal memory & executive functions component (see Fig. 6 & Table 2). Interestingly, age seemed less important for the prediction of the verbal memory & language component. In this case, education appeared to be the sole feature with a consistently high mean coefficient across algorithms and feature sets. This also fits with our univariate results, which revealed a stronger correlation between the verbal memory & language component and education than with age.

**Table 2:** Highly ranked features (according to mean coefficient) across algorithms and features sets in models with and without extra features in the concatenation approach

Model	Target		Feature	Centroid Coordinates			Desikan-Killiany Atlas **|** Julich-Brain Atlas
			Modality | Hemisphere | Network | Node	x	y	z	
− extra features	Global cognition	**1**	**GMV** LH | DMN | PFC 15	− 22	50	32	Rostral middle frontal gyrus **|** Frontal-l (Gap Map)
		**2**	**SC: within** LH | Limbic | Node 6	− 38	− 6	− 42	Inferior temporal & parahippocampal gyrus **|** Temporal-to-Parietal (Gap Map)
	Comp 1	**1**	**SC: inter** LH | Visual | Node 2	− 30	− 32	− 18	Parahippocampal & fusiform gyrus **|** Temporal-to-Parietal (Gap Map)
		**2**	**SC: inter** LH | Limbic | Node 7	− 24	6	− 40	Temporal pole & entorhinal cortex **|** Temporal-to-Parietal (Gap Map)
	Comp 2	**1**	**SC: inter** LH | Visual | Node 4	− 24	− 54	− 8	Fusiform & lingual gyrus **|** Ph1 (PhG)
		**2**	**GMV** LH | DMN | Par 7	− 48	− 60	46	Inferior parietal lobule & angular gyrus **|** PGa (IPL)
	Age	**1**	**SC: inter** LH | Visual | Node 2	− 30	− 32	− 18	Parahippocampal gyrus **|** Temporal-to-Parietal (Gap Map)
		**2**	**SC: inter** RH | Visual | Node 36	26	− 52	− 8	Parahippocampal gyrus **|** Ph3 (PhG)
		**3**	**SC: inter** RH | Limbic | Node 26	22	− 18	− 28	Fusiform & lingual gyrus **|** Temporal-to-Parietal (Gap Map)
+ extra features	Global cognition	**1**	**Age**	–	–	–	–
		**2**	**Education**	–	–	–	–
	Comp 1	**1**	**Age**	–	–	–	–
		**2**	**Education**	–	–	–	–
		**3**	**SC: inter** LH | Limbic | Node 7	-24	6	-40	Temporal pole & entorhinal cortex **|** Temporal-to-Parietal (Gap Map)
	Comp 2	**1**	**Education**	–	–	–	–

Comp 1 = non-verbal memory & executive functions component; Comp 2 = verbal memory & language component; LH = left hemisphere; RH = right hemisphere; DMN = default mode network; Visual = visual network; Limbic = limbic network; PFC = prefrontal cortex; Par = parietal

Results from the feature level were complemented by those from the modality level. Across analytic choices and cognitive targets, SC and GMV were commonly ranked as the most important modalities in the second level of the stacking approach (see Suppl. Table S49). Along the lines, FC was ranked regularly as the least important modality in the current analyses for all cognitive targets. Once the extra features, i.e., age, sex, and education, were added to the models, these were found to be the most relevant modality in all models (see Suppl. Table S49). Nevertheless, the pattern of differences between brain modalities, i.e., FC, SC, and GMV, was mostly preserved. Thus, results from the modality level, further, supported those from the feature level and emphasized that brain structural features appear more important than brain functionally derived ones in predicting cognitive performance within in the current sample of healthy older adults from the 1000BRAINS study.

#### Validation results

Prediction performance was initially compared between the PCA-derived (used in the main analysis) and a theoretically defined global cognitive score (i.e., average test performance across 14 different cognitive tests). Across different options, prediction accuracies were found to be very similar for the two definitions of global cognition (PCA-defined: Mean *R*^2^: − 0.04–0.14; theoretically defined: Mean *R*^2^: − 0.04–0.14; see Suppl. Tables S50-57 & Suppl. Fig. S58). Additionally, we investigated the classification performance of extreme groups to further substantiate findings from the main analysis. Results suggested that the multimodal input data could not reliably distinguish between extreme cognitive groups with best performing models achieving only 65% accuracy (Mean accuracy: 45.5–65.4%; see Suppl. Tables S59 & Suppl. Fig. S60). As groups were matched for all confounders, these results further substantiated findings from our main analyses in the deconf. condition. Moreover, including RSFC estimates derived from negative correlations as additional input features (i.e., FSet C) revealed a relatively similar pattern of results as observed in the main analysis (FSet C: Without extra features: Mean *R*^2^: 0.05–0.14 (no-deconf.)/-0.01–0.01 (deconf.); with extra features: Mean *R*^2^: 0.10–0.34; FSet A&B: Without extra features: Mean *R*^2^: 0.02–0.14 (no-deconf.) / − 0.02–0.01 (deconf.); with extra features: Mean *R*^2^: 0.12–0.34; see Suppl. Tables S8, S13, S21, S23, S61-63 & Suppl. Fig. S64). Similarly as in the main analysis, FC estimates were found to lead to lowest prediction performance compared to SC estimates and region-wise GMV (see Suppl. Tables S61-63 & Suppl. Fig. S64). Thus, the inclusion of negative edge values in the estimation of RSFC estimates did not seem to boost signal for the ML models. Furthermore, to validate our ML pipeline and gain a greater insight into the confounding variables, we examined the predictability of age, sex, and educational level from our input features. Age (Mean *R*^2^: 0.05–0.44; see Suppl. Tables S65-66 & Suppl. Fig. S67) and sex (Mean accuracy: 60.5–83.0%; see Suppl. Tables S68 & Suppl. Fig. S69) could be predicted with high accuracies. In contrast, educational level could be predicted less reliably from our features (Mean *R*^2^: − 0.45–0.04; see Suppl. Tables S70 & Suppl. Fig. S71).

## Discussion

The aim of the current study was to investigate the general validity of the prediction of cognition from imaging data in healthy older adults. Thereby, we were specifically interested in whether (1) integrating information from a network perspective, i.e., RSFC and SC estimates, with morphological brain data, i.e., region-wise GMV, may lead to better prediction performance of different cognitive targets than unimodal models, (2) global cognition and distinct cognitive profiles differ in their predictability from imaging data, and (3) results generalize across different ML pipeline configurations and approaches, i.e., different modality combinations, algorithms, feature sets, deconfounding analyses and multimodal approaches, in a large sample of healthy older adults from the 1000BRAINS study. Across a variety of different analytic choices, moderate prediction performance of cognitive variables could solely be observed in absence of confounder control. In this context, we found only a slight trend for better predictability in multimodal than unimodal models, higher prediction accuracies for SC and GMV than RSFC and for global cognition compared to specific cognitive profiles. Noticeably, once age, sex, and education were controlled for, all previously reported effects disappeared and rather low predictability was observed. Subsequent analyses showed that demographic variables alone already explained a substantial amount of variance in the target variables. Thus, results emphasize despite a small potential benefit of a multimodal approach, the considerable impact of factors such as age, sex, and education on the prediction of cognitive targets in healthy older adults.

Cognition emerges from the complex interaction of multiple organizational levels in the brain. As such, differences in structural and functional brain network architecture as well as in morphological brain features have been related to cognitive performance differences in older age [1, 3–13]. In terms of prediction, most prior studies have focused on the usage of single modalities to predict cognitive ability in healthy older adults. A multimodal approach, however, may allow for a more complete description of age-related cognitive decline than each single modality as aging has been found to affect the brain at all levels [67]. Initial encouraging results in different samples have demonstrated that the use of multimodal data may improve prediction performance for different cognitive abilities, e.g., fluid intelligence, global cognitive function, visual working memory, fluid reasoning, vocabulary [26, 31, 33, 55, 68]. For example, multimodal models, including information from structural and functional imaging, yielded improved prediction accuracies of up to *R*^2^ = 0.05 compared to *R*^*2*^ = 0.02–0.04 in unimodal models for fluid intelligence in a large sample from the UK Biobank [31]. Similarly, in a longitudinal setting, changes in a clinical score, i.e., Clinical dementia rating (CDR), were found to be predicted with higher accuracies from different multimodal models (*R*^2^ range = 0.34–0.42), including non-brain information and brain features, than from single modalities (*R*^2^ range = 0.01–0.28) in a large sample from the OASIS-3 project [32]. Our findings extend prior research by revealing moderate prediction performance of different cognitive variables (global and domain-specific) across different analytic choices using combined parameters of brain structure and network architecture, i.e., region-wise GMV, RSFC, and SC estimates, and no demographic deconfounding. In the no deconfounding conditions, the best performing unimodal model (SC estimates) was found to explain up to 11% of variance (*R*^2^) in our global cognitive target, while the best multimodal model (GMV + RSFC + SC) explained 14% of variance (*R*^2^). In terms of magnitude of prediction performance, current results, thus, fall into the range of what has been reported in prior studies. Noticeably, this hints at a slight benefit of integrating information across different imaging modalities for the prediction of cognition in healthy aging.

Focusing on the single modalities, the lowest predictability was encountered for RSFC estimates. This further substantiates results from previous analyses of limited predictive potential of RSFC strength measures in different feature set combinations and hints at variations in prediction potential of RSFC for cognitive targets [24, 31, 54, 55, 67, 69]. For example, RSFC data led to lower prediction results (*R*^2^ = 0.01) than anatomical markers (*R*^2^ = 0.28), e.g., mean cortical thickness, cerebral GMV, and volumes of subcortical areas, in predicting cognitive decline (CDR change) in a sample of older adults from the OASIS-3 project [32]. Thus, it appears that cognitive performance differences in older age may be less clearly encoded in functional connectivity, especially in RSFC estimates, but more so in brain structural information. This may be due to the fact that brain function, i.e., RSFC and task-based FC, responds more adaptively to aging. Aging is accompanied by both increases and decreases in RSFC, which successively have been related to cognitive performance alterations [70]. Importantly, it has been postulated that the brain may engage into compensatory scaffolding and the recruitment of additional neural resources, e.g., connectivity, in an attempt to maintain cognitive function, when confronted with brain functional and structural decline [71, 72]. In this context, whether the additional neural response will lead to preserved cognition, will depend on the degree of scaffolding available and with it on the extent of neural insults that might have already taken place [71, 72]. Thus, it may be argued that age-related RSFC alterations and their relation to cognition are subject to high variability, which may complicate a clear mapping between RSFC patterns and cognitive performance in prediction. In contrast, age-related structural decline once having reached a sufficient degree typically results in cognitive performance decreases [73–76]. This clear correspondence may, in turn, be well captured by ML prediction models and may explain the moderate predictability based on SC estimates and region-wise GMV in the current study. Current results, in turn, emphasize that brain structural measures may be central to cognitive aging and suggest a prediction power advantage of brain structural information over RSFC patterns for cognitive abilities in older age [77].

Some cognitive functions are more strongly affected than others during the aging process, e.g., executive and memory functions [1]. This may also be expressed in different extents of predictability. To investigate this further, we considered different cognitive targets in our sample of older adults, i.e., global cognition and distinct cognitive profiles, in the present study. Results showed that global cognition was best predicted, followed by the non-verbal memory & executive functions component and finally the verbal memory & language component across analytic choices in the no-deconf. condition. One potential explanation for the performance benefit of global cognition over specific cognitive profiles may be related to cognitive aging being thought of as a largely domain-general process [78–81]. As such, it may be argued that general cognitive performance differences in older age may be much more prominent and in turn may also be more detectable at the whole-brain level than domain-specific alterations. In terms of relevant features for prediction, results revealed regions in the frontal and temporal lobe to be most predictive, which have been implicated in healthy and pathological aging as well as have been associated with age-related cognitive decline [82–86]. Specifically, our results suggest that alterations in the communication within the limbic network and structural properties of the middle frontal gyrus in the DMN may be critical for identifying individual differences in global cognitive performance in older age.

The non-verbal memory & executive functions component was predicted second best. Highest loadings on this component were found for cognitive tests on problem-solving, figural memory as well as visual and visual-spatial WM. The structural wiring of the parahippocampal/fusiform gyrus (visual network) and temporal pole/entorhinal cortex (limbic network) to other networks throughout the brain were found to be important for prediction. Thus, predictive features spanned regions that are typically thought to be involved in cognitive tasks related to visual and memory-related processes [87–95]. Thus, global and domain-specific cognitive functions may not only be captured by distinct neural correlates, but may also differ in their most predictive features.

Interestingly, lowest prediction performance was observed for the verbal memory & language component in the current investigation. Results from prior prediction studies with older adults fit this account [26, 96, 97]. For example, language functions (HCP-A: *r* = 0.23, BARBI: *r* = 0.12) have been shown to lead to lower prediction performance than executive functions (HCP-A: *r* = 0.32, BARBI: *r* = 0.28) and attention (HCP-A: *r* = 0.37, BARBI: *r* = 0.25) in two independent samples based on SC data [96]. Thus, results are comparable to our SC results. Across algorithms, feature sets and multimodal approaches, we found correlation values between true and predicted scores to range from *r* = 0.19 to 0.34 for global cognition and non-verbal memory & executive functions, while for the verbal memory & language component smaller correlation values in a range of *r* = 0.08 to 0.23 were observed. Language functions, thus, not only appear to differ in aging trajectories (e.g., tend to remain more stable than for example executive and memory functions), but also in their predictability to other cognitive domains, e.g., processing speed, memory and executive functions, in older aged individuals [97]. A potential explanation may be that factors like education or occupational attainment may be highly relevant for the prediction of language-related cognitive performance overshadowing the predictive utility of brain features [26, 98]. This is also supported by the feature importance analyses in the current study. Without the addition of extra features, relevant regions for prediction included parts of the lingual/fusiform/parahippocampal gyrus (visual network) and the inferior parietal lobule/angular gyrus (DMN), which not only seem to be involved in different language-related functions, but also to be predictive of language abilities in older age [17, 99–102]. However, once added to the ML models, educational level appeared to be the most important feature for the prediction of verbal memory & language and with it to explain a large portion of variance in the target, which corresponds to prior research reporting strong associations between language measures and educational level [103, 104]. Current findings, thus, add to previous research by emphasizing the unique role of language functions in aging and stressing the intricate link to educational measures in older age.

Importantly, all previously described effects of successful prediction and emerging differences between modalities and cognitive targets were no longer encountered, once age, sex and education were controlled for. The significant drop in prediction performance after confounder control has to some degree also been reported in former studies [15, 18, 105]. For example, Kwak et al. reported a drop in mean prediction accuracy of neuropsychological test performance from RSFC in models adjusted for age (without confounder control: *r* = 0.253, adjustment for age: *r* = 0.179) [18]. Nevertheless, different cognitive targets could still be successfully predicted in healthy older adults after controlling for demographic factors across various studies. A potential explanation for divergent results in the current study compared to studies reporting successful prediction even after confounder control may be differences in samples, ML approaches, features, and targets used.

Therefore, to further evaluate the relevance of demographic variables in the prediction setting we investigated the individual contributions of age, sex, and education to the prediction by including these as extra features to the ML model. We found that the addition of age, sex, and education to our brain models drastically increased predictability of cognitive targets, in line with prior studies [31, 32, 55, 106, 107]. For example, Dadi et al. showed that fluid intelligence and neuroticism were more successfully predicted when sociodemographic information was included into the model in a large sample from the UK Biobank (*N* = 11,175) [31]. Similarly, Rasero et al. found that multimodal brain features together with age, sex, and education led to a prediction performance increase from median *R*^2^ = 0.078 to median *R*^2^ = 0.197 for global cognition [55]. Dadi et al. even reported fluid intelligence prediction based on all sociodemographic measures to perform slightly better without (*R*^2^ = 0.17) than with brain imaging (*R*^2^ = 0.16) [31]. The high relevance of demographic features for prediction was also mirrored in the current study. Present findings showed that joined models of brain features and demographic variables perform similar or even worse than models based only on the demographic features. Age, sex, and education were thereby found to reliably rank in the top features in joined models of brain and demographic features. Thus, it appears that the brain features, i.e., region-wise GMV, RSFC, and SC estimates, did not add substantial information to the prediction of cognitive performance in our older sample. Jointly, current results from the confounder analyses particularly accentuate the high impact of age, sex, and education and the limited informational value of currently employed brain features in the prediction of different cognitive variables in a large sample of healthy older adults. Given that age, sex, and education may have a substantial influence on prediction performance, it appears highly important to consider the influence of demographic features on results in future prediction studies in healthy aging. Along the lines, results from ML prediction without control for demographic factors should be considered with caution as results may not show the true predictive power of respective input features.

### Methodological considerations and future outlook

In the current study, we employed both a concatenation and stacking approach to examine whether performance benefits may be observed for one over the other. Against initial predictions, the stacking approach did not reliably boost prediction accuracies [54–56, 58, 62]. Results from both approaches were found to be more or less comparable across a wide range of algorithms, feature sets, deconfounding strategies, and cognitive targets. Thus, current results provide further sustenance to prior work showing that a stacking benefit may not always be observed and different approaches should be compared to delineate, which one offers the best results for the question at stake [108].

Furthermore, it should be pointed out that a functionally derived cortical brain parcellation was used for all input modalities in the current study. The 400-nodes Schaefer parcellation was applied for RSFC, SC, and GMV to ensure comparability between modalities and to other prediction and lifespan studies [44, 50]. In future prediction studies, it might be valuable to explore the addition of subcortical regions, which are not covered by the current parcellation and have been shown to be highly relevant for distinct cognitive processes [109].

Another aspect to consider is that a significance-based threshold derived from null models based on randomization of time series information and permutation testing was included for resting-state connectivity matrices in the present study [11, 12, 51, 110]. While there are various studies that utilize resting-state connectivity matrices without a threshold, it was implemented here to reduce the amount of spurious correlations, which have been frequently encountered in RSFC [11, 12, 51, 53, 110–113]. Despite the potential of smaller correlations carrying meaningful information, no thresholding bears the risk of adding further noise into the analyses [11, 12]. As such, we have decided on a more conservative approach of using a threshold [53]. Furthermore, given that prediction performance appears generally low for FC based on the thresholded correlation matrices, we would anticipate that including those potentially smaller correlation values would not significantly impact ML prediction performance and boost the overall signal in the FC data, but rather add further noise to the ML models.

Additionally, it might become necessary in future studies to include other information about the aging process into prediction models for cognitive performance and prospective future cognitive decline. In the current study, we specifically investigated the use of RSFC and SC estimates due to the role of brain network patterns in aging and cognition. Nonetheless, their computation inherently includes a dimensionality reduction step and the loss of potentially relevant information. Similar to studies in younger cohorts, the use of raw connectivity measures (RSFC & SC) may be explored in future studies targeting the prediction of cognitive performance in older age. Moreover, one might consider adding FC dynamics and task-based fMRI information to prediction models of cognitive variables in older age [114–117]. Beyond brain features, it may also be interesting to integrate non-brain information that may be relevant in terms of cognitive aging into ML models, such as genetic information, health or environmental features, to further improve and stabilize models [118].

In addition, newest studies have revealed that samples > 1000 or larger may be necessary to reliably detect brain-behavior relations with small effect sizes [68, 119, 120]. In this realm, our sample of *N* = 594 may not be large enough to obtain robust findings and higher prediction accuracies.

Moreover, the current study focused solely on a cross-sectional examination of prediction potential of cognitive performance in older age. To develop a marker for prospective cognitive decline in the future, it becomes necessary to shift attention to the investigation of longitudinal data and whether specific brain patterns may relate to later cognitive performance of an individual [121, 122].

## Conclusions

The present study addressed the universality of cognitive performance prediction from imaging data in a large sample of healthy older adults using different ML approaches. Specifically, the benefit of integrating information across brain structure, i.e., region-wise GMV, and network organization, i.e., region-wise GMV, RSFC, and SC estimates, for the prediction of cognition compared to unimodal models as well as predictability differences between global cognition and two cognitive profiles were examined across a systematic analysis of different ML pipeline configurations. Present findings hint at moderate prediction performance of different cognitive targets from multimodal data in absence of confounder control. In this setting, we observed a small tendency for multimodal outperforming unimodal models in terms of prediction accuracy. Additionally, we observed higher predictability based on structural compared to functional brain features as well as better predictability of global cognition in comparison to distinct cognitive profiles. After controlling for age, sex, and education, previously described effects vanished stressing the intricate link between cognition and demographic factors at the brain level. Thus, present results emphasize the importance of considering these variables, i.e., age, sex, and education, in aging studies using a prediction framework. Furthermore, in future studies, it appears warranted to consider the usage of alternative input features in the search for a marker for age-related cognitive decline. Overall, present results suggest that although multimodal data may be beneficial for prediction of cognitive functioning in older cohorts, developing a marker for age-related cognitive decline may be aggravated by the influence of, e.g., demographic factors.

### Supplementary Information

Below is the link to the electronic supplementary material.

Supplementary file1 (PDF 4816 KB)

## Data Availability

Due to local regulations of data acquisition and usage, data of 1000BRAINS are available upon request from the responsible PI.
