# Supplementary material for: Prediction of cognitive performance differences in older age from multimodal neuroimaging data
Source: GeroScience. 2023 Jun 13;46(1):283–308. doi: 10.1007/s11357-023-00831-4 (PMC10828156; doi:10.1007/s11357-023-00831-4)
Supplement: Supplementary file 1 — Supplementary file1 (PDF 4816 KB) [file 11357_2023_831_MOESM1_ESM.pdf]

Supplementary Material

Supplementary Methods

Supplementary Table S1. Overview and description of neuropsychological tests employed in the current study to assess cognitive performance.

Name	Test	Description	Mean (raw ± SD)	Z-score			Function	Domain
				Min	Max	Range		
AKT	Alters-Konzentrations-Test (Gatterer, 2008)	Time (sec.) to detect and cross targets among distractors	34.13 ± 10.58	-1.62	8.31	9.93	Selective Attention	Atten-tion
TMT-A	Trail Making Test (part A) (taken from CERAD-Plus; Morris et al., 1989)	Time (sec.) to link numbers distributed randomly in ascending order (part A)	40.04 ± 13.89	-1.73	6.32	8.05	Processing speed	
Stroop	Farb-Wort-Interferenztest (Jülich version; similar to: Bäumler, 1985; Stroop, 1935)	Time difference (sec.) between naming the ink in which color words were printed (part 3) and naming the color of boxes (part 2)	41.83 ± 19.36	-1.97	5.98	7.95	Susceptibility to interference	Executive functions
FPT	Fünf-Punkte-Test (Jülich version; similar to: Regard et al., 1982)	Total number of unique patterns created in (3 minutes) by connecting 5 dots	26.59 ± 7.42	-3.05	3.43	6.47	Figural fluency	
LPS	Leistungsprüfungssystem 50+ (Subtest 3) (Sturm et al., 1993)	Total number of correctly identified irregularities in sequences of geometric figures (5 minutes)	20.77 ± 5.05	-2.53	2.82	5.35	Problem solving	
TMT-BA	Trail Making Test (part B-A) (taken from CERAD-Plus; Morris et al., 1989)	Time difference (sec.) between connecting numbers and letters in ascending order in an alternating fashion (part B) and (part A)	52.8 ± 36.28	-1.47	8.79	10.26	Concept shifting	
AWSTOP3	Wortschatztest (Schmidt & Metzler, 1992)	Total number of correctly identified real words among rows of pseudo words	31.07 ± 4.62	-4.56	2.15	6.71	Vocabulary	Language
RWT-S	Regensburger Wortflüssigkeitstest (Aschenbrenner et al., 2000)	Total number of produced words in the category "Berufe (job)" (2 minutes)	24.18 ± 6.80	-2.67	3.06	5.73	Semantic verbal fluency	
RWT-P	Regensburger Wortflüssigkeitstest (Aschenbrenner et al., 2000)	Total number of produced words beginning with the letter B (2 minutes)	18.64 ± 6.36	-2.30	3.20	5.50	Phonemic verbal fluency	
Benton	Benton-Test (Benton et al., 2009)	Total number of errors made during the free recall of 20 previously presented figures	16.28 ± 8.05	-1.9	3.57	5.47	Figural memory	Episodic memory
BKW	Verbaler Gedächtnistest (Lux et al., 2012)	Total number of free recalled words from a list of 15 words across 5 trials (sum score)	42.70 ± 10.35	-3.55	2.25	5.8	Verbal episodic memory	
CBT	Block-Tapping-Test (Schelling, 1997)	Total number of correctly repeated blocks given in a sequence (sum score backward and forward)	10.14 ± 1.58	-4.51	3.07	7.58	Visual spatial working memory	Working memory
VPT	Visual pattern (Jülich version; similar to: Della Sala et al., 1997)	Total number of correctly memorized matrix patterns of black and white squares with increasing complexity	7.82 ± 1.81	-2.11	2.31	4.43	Visual working memory	
ZNS	Zahlennachsprechen (from Nürnberger Alters-Inventar) (Oswald & Fleischmann, 1997)	Total number of correctly recalled digits given in a sequence (sum score backward and forward)	10.82 ± 1.8	-2.69	3.44	6.13	Verbal working memory	

Further descriptions are also found in Caspers et al., 2014 as well as Jockwitz et al., 2017.

Validation analyses

Sex classification

Supplementary Table S2. Demographic information for matched (for age, education & eTIV) sample used in validation analyses for sex classification.

	Extreme Sample		
	N	M_{age}	Education
Female	170	66.59 (6.67)	6.41 (1.96)
Male	170	66.56 (6.88)	6.55 (1.88)
Total	340	66.57 (6.77)	6.48 (1.92)

Note. Standard deviation (SD) appears in parentheses.

Classification of extreme cognitive groups (high vs. low cognitive performance group)

Supplementary Table S3. Demographic information for extreme cognitive sample used in validation analyses (matched for age, education, sex & eTIV).

	Extreme Sample		
	N	M_{age}	Education
Female	56	64.18 (5.25)	5.75 (1.61)
Male	60	67.49 (6.36)	6.53 (1.79)
Total	116	65.89 (6.06)	6.16 (1.74)

Note. Standard deviation (SD) appears in parentheses.

Supplementary Table S4. Demographic information for high and low performance groups in extreme sample used in validation.

	N	M_{age}	Education
High	58	65.33 (6.07)	6.12 (1.8)
Low	58	66.45 (5.68)	6.19 (1.7)

Note. Standard deviation (SD) appears in parentheses.

Supplementary Results

Supplementary Table S5. Explained variance and eigenvalues from PCA.

Component	Initial Eigenvalues			Rotation Sums of Squared Loadings		
	Total	% of Variance	Cumulative %	Total	% of Variance	Cumulative %
1	5.228	37.345	37.345	5.228	37.345	37.345
2	1.331	9.508	46.853	1.331	9.508	46.853
3	.954	6.814	53.667			
4	.863	6.163	59.830			
5	.769	5.490	65.320			
6	.713	5.091	70.411			
7	.680	4.860	75.271			
8	.608	4.340	79.611			
9	.569	4.066	83.677			
10	.542	3.870	87.547			
11	.486	3.474	91.021			
12	.451	3.223	94.244			
13	.417	2.982	97.226			
14	.388	2.774	100.000			

Supplementary Figure S7. Scree plot of initial eigenvalues derived from PCA.

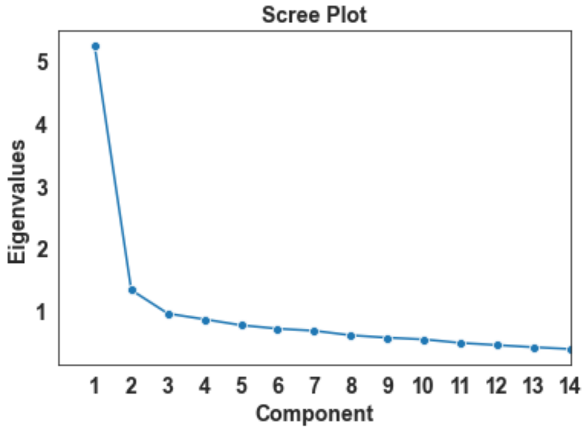

Supplementary Table S6. Factor loadings from PCA.

Cognitive Test	Factor Loadings		
	Global Cognition	Component 1: EF & Memory	Component 2: Language
FPT	0.666	0.509	0.431
LPS	0.761	0.694	0.344
AWSTO3P	0.607	0.266	0.651
RWT-P	0.519	0.072	0.760
RWT-S	0.561	0.126	0.759
BKW	0.545	0.258	0.56
CBT	0.497	0.705	-0.103
VPT	0.625	0.758	0.038
ZNS	0.572	0.497	0.291
TMT-A	0.588	0.546	0.253
AKT	0.606	0.554	0.274
TMT-BA	0.608	0.488	0.363
Stroop	0.563	0.491	0.284
Benton	0.769	0.698	0.354

Machine learning results

Abbreviations

FC	Resting-state functional connectivity estimates
SC	Structural connectivity estimates
GMV	Region-wise grey matter volume
CF	Confounders: age, sex and education
App.	Approach
Concat	Concatenation approach
Stack	Stacking approach
Cond.	Conditions
Feature Set (FSet) A	Within- and inter-NW connectivity for FC and SC + GMV
Feature Set (FSet) B	Ratio-score for FC and SC + GMV
nr	No confound regression, i.e. validation analyses
no-deconf.	No deconfounding except target controlled for eTIV
deconf.	Age, education, sex & eTIV regressed from target
Algo.	Algorithms
EN	Elastic Net Regression
RF	Random Forest Regression/Classification
SVR	Linear Support Vector Regression
Ridge	Ridge Regression/Classification
Lasso	LASSO Regression
Log	Logistic Regression
SVC	Linear Support Vector Classification
MAE	Mean Absolute Error
R^2	Coefficient of Determination
folds > ref.	Percentage of folds better than reference model (dummy) in terms of R^2 (regression) or accuracy (classification); Colour scheme: green = real model outperforms dummy in ≥ 80% of folds; orange = real model outperforms dummy in 50 - 80% of folds; red = real model outperforms dummy in ≤ 50% of folds.
B_{all}	Mean ML performance$_{\text{Multimodal model}}$ – Mean ML performance $_{\text{Unimodal models}}$; Colour scheme: green = benefit > 0; orange = same performance in multi- & unimodal model (0); red = benefit < 0
B_{best}	Mean ML performance$_{\text{Multimodal model}}$ – Mean ML performance$_{\text{Best unimodal models}}$; Colour scheme: green = benefit > 0; orange = same performance in multi- & unimodal model (0); red = benefit < 0

Supplementary Table S8. Regression performance for composite cognition in the concatenation approach in FSet A.

**FSet A**	**Global**	Unimodal			Multimodal			
	**Concat**	FC	SC	GMV	FC+ GMV	SC+ GMV	FC+ SC	FC+SC+ GMV
Cond.	Algo.	MAE	MAE	MAE	MAE	MAE	MAE	MAE
no-deconf.	EN	0.78 (0.03)	0.76 (0.04)	0.76 (0.04)	0.76 (0.04)	0.74 (0.05)	0.75 (0.04)	0.74 (0.04)
	RF	0.78 (0.03)	0.76 (0.04)	0.77 (0.03)	0.76 (0.03)	0.75 (0.04)	0.76 (0.04)	0.75 (0.03)
	SVR	0.77 (0.04)	0.75 (0.04)	0.77 (0.04)	0.75 (0.04)	0.74 (0.05)	0.75 (0.04)	0.74 (0.04)
	Ridge	0.78 (0.03)	0.76 (0.05)	0.76 (0.04)	0.76 (0.04)	0.74 (0.05)	0.76 (0.04)	0.74 (0.04)
	Lasso	0.78 (0.03)	0.76 (0.03)	0.78 (0.03)	0.77 (0.03)	0.75 (0.04)	0.75 (0.03)	0.75 (0.04)
deconf.	EN	0.79 (0.03)	0.79 (0.03)	0.79 (0.03)	0.79 (0.03)	0.79 (0.03)	0.79 (0.03)	0.79 (0.03)
	RF	0.80 (0.03)	0.80 (0.03)	0.79 (0.03)	0.79 (0.03)	0.79 (0.03)	0.79 (0.03)	0.79 (0.03)
	SVR	0.79 (0.03)	0.80 (0.03)	0.79 (0.03)	0.79 (0.03)	0.79 (0.03)	0.79 (0.03)	0.79 (0.04)
	Ridge	0.79 (0.03)	0.79 (0.03)	0.79 (0.03)	0.79 (0.03)	0.79 (0.03)	0.79 (0.03)	0.79 (0.03)
	Lasso	0.79 (0.03)	0.79 (0.03)	0.79 (0.03)	0.79 (0.03)	0.79 (0.03)	0.79 (0.03)	0.79 (0.03)

**FSet A**	**Global**	Unimodal						Multimodal							
	**Concat**	FC		SC		GMV		FC+ GMV		SC+ GMV		FC+ SC		FC+SC+GMV	
Cond.	Algo.	R^2	folds > ref.	R^2	folds > ref.	R^2	folds > ref	R^2	folds > ref	R^2	folds > ref	R^2	folds > ref	R^2	folds > ref
no-deconf.	EN	0.04 (0.06)	76%	0.11 (0.07)	93%	0.08 (0.06)	89%	0.09 (0.07)	91%	0.13 (0.08)	94%	0.11 (0.07)	93%	0.14 (0.07)	95%
	RF	0.04 (0.05)	79%	0.09 (0.05)	96%	0.06 (0.05)	89%	0.08 (0.05)	92%	0.11 (0.05)	98%	0.09 (0.06)	95%	0.11 (0.05)	98%
	SVR	0.04 (0.06)	74%	0.10 (0.07)	90%	0.06 (0.07)	86%	0.09 (0.07)	89%	0.13 (0.08)	90%	0.10 (0.07)	92%	0.12 (0.08)	92%
	Ridge	0.03 (0.06)	76%	0.10 (0.08)	90%	0.09 (0.05)	93%	0.08 (0.07)	91%	0.13 (0.08)	93%	0.09 (0.08)	86%	0.12 (0.08)	92%
	Lasso	0.03 (0.03)	83%	0.10 (0.05)	96%	0.05 (0.03)	94%	0.06 (0.04)	95%	0.12 (0.05)	99%	0.11 (0.05)	98%	0.12 (0.05)	99%
deconf.	EN	0.00 (0.02)	55%	0.00 (0.02)	30%	0.01 (0.02)	77%	0.00 (0.04)	61%	0.01 (0.02)	62%	0.00 (0.03)	55%	0.01 (0.03)	61%
	RF	-0.01 (0.04)	44%	-0.01 (0.04)	39%	0.01 (0.04)	57%	0.00 (0.04)	52%	0.00 (0.04)	52%	0.00 (0.03)	49%	0.00 (0.04)	58%
	SVR	-0.01 (0.04)	48%	0.00 (0.03)	46%	0.00 (0.03)	63%	0.00 (0.06)	58%	0.00 (0.03)	57%	-0.01 (0.05)	44%	0.00 (0.06)	51%
	Ridge	0.00 (0.03)	53%	0.00 (0.02)	67%	0.01 (0.02)	77%	0.00 (0.04)	66%	0.01 (0.02)	73%	0.00 (0.02)	63%	0.00 (0.04)	64%
	Lasso	0.00 (0.01)	6%	0.00 (0.00)	5%	0.00 (0.01)	7%	0.00 (0.01)	5%	0.00 (0.01)	4%	0.00 (0.01)	5%	0.00 (0.01)	4%

Supplementary Table S9. Regression performance (correlation) for composite cognition in the concatenation approach in FSet A.

**FSet A**	**Global**	Unimodal			Multimodal			
	**Concat**	FC	SC	GMV	FC+ GMV	SC+ GMV	FC+ SC	FC+SC+GMV
Cond.	Algo.	r	r	r	r	r	r	r
no-deconf.	EN	0.21 (0.04)	0.33 (0.01)	0.28 (0.02)	0.30 (0.01)	0.37 (0.01)	0.34 (0.02)	0.37 (0.03)
	RF	0.20 (0.03)	0.31 (0.01)	0.25 (0.01)	0.28 (0.01)	0.33 (0.01)	0.31 (0.02)	0.34 (0.01)
	SVR	0.22 (0.02)	0.33 (0.01)	0.27 (0.02)	0.31 (0.01)	0.37 (0.01)	0.33 (0.02)	0.36 (0.02)
	Ridge	0.19 (0.04)	0.33 (0.01)	0.30 (0.01)	0.30 (0.02)	0.36 (0.01)	0.31 (0.02)	0.35 (0.01)
	Lasso	0.18 (0.04)	0.33 (0.01)	0.24 (0.03)	0.26 (0.03)	0.35 (0.01)	0.35 (0.01)	0.36 (0.01)
deconf.	EN	0.04 (0.02)	0.02 (0.06)	0.11 (0.01)	0.08 (0.03)	0.08 (0.02)	0.05 (0.04)	0.10 (0.04)
	RF	0.05 (0.04)	0.04 (0.02)	0.10 (0.03)	0.09 (0.02)	0.09 (0.02)	0.07 (0.02)	0.08 (0.04)
	SVR	0.06 (0.02)	0.05 (0.01)	0.08 (0.03)	0.10 (0.02)	0.09 (0.02)	0.06 (0.01)	0.09 (0.02)
	Ridge	0.03 (0.03)	0.04 (0.02)	0.11 (0.02)	0.09 (0.04)	0.09 (0.01)	0.05 (0.02)	0.08 (0.02)
	Lasso	-0.05 (0.04)	-0.01 (0.01)	-0.02 (0.04)	-0.03 (0.04)	-0.01 (0.00)	-0.02 (0.04)	-0.02 (0.02)

Supplementary Figure S10. Regression performance (correlation) for composite cognition in the concatenation approach across feature sets in the concatenation approach.

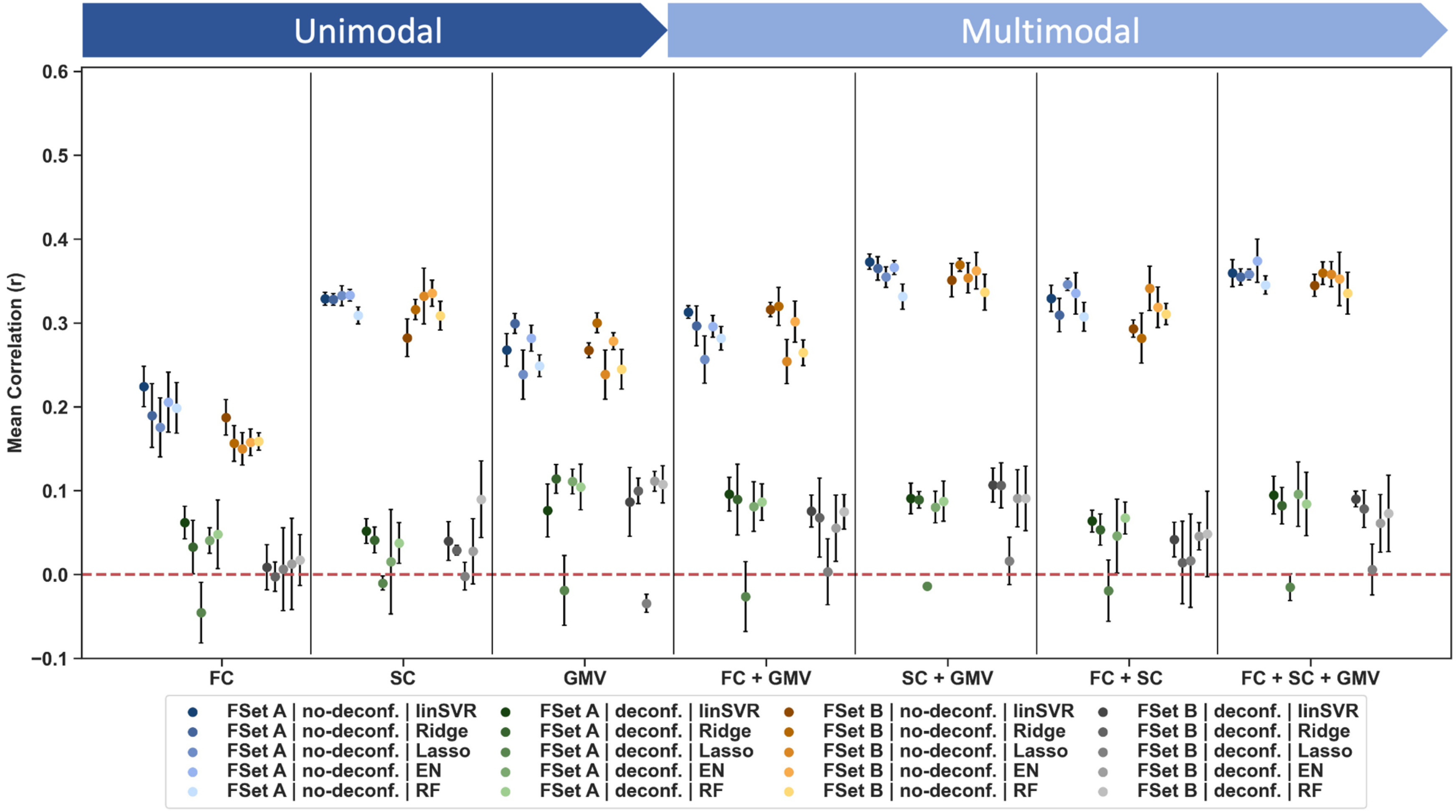

Note. no-deconf. = no deconfounding except for controlling for eTIV in target, deconf. = confound regression of age, sex, education & eTIV.

Supplementary Table S11. Regression performance for composite cognition in the stacking approach in FSet A.

**FSet A**	**Global**	Unimodal			Multimodal				Unimodal						Multimodal							
	**Stack**	FC	SC	GMV	FC+ GMV	SC+ GMV	FC+ SC	FC+SC +GMV	FC		SC		GMV		FC+ GMV		SC+ GMV		FC+ SC		FC+SC+GMV	
Cond.	Algo.	MAE	MAE	MAE	MAE	MAE	MAE	MAE	R^2	folds > ref.	R^2	folds > ref.	R^2	folds > ref.	R^2	folds > ref.	R^2	folds > ref.	R^2	folds > ref.	R^2	folds > ref.
no-deconf.	EN	0.78 (0.03)	0.76 (0.04)	0.76 (0.04)	0.76 (0.04)	0.75 (0.05)	0.76 (0.04)	0.75 (0.04)	0.04 (0.05)	78%	0.11 (0.07)	94%	0.08 (0.06)	89%	0.08 (0.07)	87%	0.10 (0.08)	88%	0.09 (0.08)	86%	0.12 (0.08)	92%
	RF	0.78 (0.03)	0.76 (0.04)	0.78 (0.04)	0.78 (0.04)	0.76 (0.05)	0.77 (0.04)	0.76 (0.04)	0.04 (0.05)	76%	0.09 (0.06)	95%	0.06 (0.06)	87%	0.04 (0.07)	73%	0.08 (0.08)	86%	0.07 (0.08)	82%	0.09 (0.08)	86%
	SVR	0.77 (0.04)	0.75 (0.04)	0.77 (0.04)	0.76 (0.04)	0.76 (0.05)	0.76 (0.04)	0.75 (0.04)	0.04 (0.06)	75%	0.10 (0.07)	90%	0.06 (0.07)	82%	0.09 (0.07)	91%	0.10 (0.08)	88%	0.10 (0.07)	90%	0.12 (0.08)	94%
	Ridge	0.78 (0.04)	0.76 (0.05)	0.76 (0.04)	0.76 (0.04)	0.75 (0.05)	0.76 (0.04)	0.75 (0.05)	0.03 (0.06)	74%	0.10 (0.08)	90%	0.09 (0.05)	93%	0.09 (0.07)	85%	0.11 (0.08)	91%	0.09 (0.08)	82%	0.12 (0.08)	92%
	Lasso	0.78 (0.03)	0.76 (0.03)	0.78 (0.03)	0.78 (0.03)	0.76 (0.04)	0.76 (0.04)	0.76 (0.04)	0.03 (0.03)	83%	0.10 (0.05)	96%	0.05 (0.03)	94%	0.04 (0.05)	82%	0.09 (0.08)	89%	0.09 (0.09)	83%	0.10 (0.07)	91%
deconf.	EN	0.79 (0.03)	0.80 (0.03)	0.79 (0.03)	0.80 (0.03)	0.80 (0.04)	0.80 (0.03)	0.80 (0.03)	0.00 (0.03)	56%	0.00 (0.02)	32%	0.01 (0.02)	74%	-0.01 (0.04)	42%	-0.01 (0.04)	47%	-0.02 (0.05)	40%	-0.01 (0.04)	45%
	RF	0.80 (0.03)	0.80 (0.03)	0.79 (0.03)	0.80 (0.03)	0.80 (0.04)	0.80 (0.04)	0.80 (0.04)	-0.01 (0.04)	38%	-0.01 (0.04)	39%	0.00 (0.04)	54%	-0.01 (0.04)	37%	-0.02 (0.05)	35%	-0.02 (0.04)	27%	-0.02 (0.05)	37%
	SVR	0.79 (0.03)	0.80 (0.03)	0.79 (0.03)	0.80 (0.03)	0.80 (0.03)	0.80 (0.03)	0.80 (0.03)	-0.01 (0.04)	48%	0.00 (0.02)	45%	0.00 (0.03)	67%	-0.02 (0.04)	40%	-0.02 (0.04)	30%	-0.02 (0.05)	35%	-0.01 (0.04)	41%
	Ridge	0.79 (0.03)	0.79 (0.03)	0.79 (0.03)	0.80 (0.03)	0.80 (0.03)	0.80 (0.03)	0.80 (0.03)	0.00 (0.02)	58%	0.00 (0.02)	66%	0.01 (0.03)	77%	-0.02 (0.04)	38%	-0.02 (0.05)	38%	-0.03 (0.04)	28%	-0.02 (0.04)	38%
	Lasso	0.79 (0.03)	0.79 (0.03)	0.79 (0.03)	0.80 (0.03)	0.80 (0.03)	0.80 (0.03)	0.80 (0.03)	0.00 (0.01)	7%	0.00 (0.01)	5%	0.00 (0.01)	10%	-0.01 (0.02)	7%	0.00 (0.02)	7%	-0.01 (0.01)	3%	-0.01 (0.02)	7%

Supplementary Table S12. Regression performance (correlation) for composite cognition in the stacking approach in FSet A.

**FSet A**	**Global**	Unimodal			Multimodal			
	**Stack**	FC	SC	GMV	FC+ GMV	SC+ GMV	FC+ SC	FC+SC+GMV
Cond.	Algo.	r	r	r	r	r	r	r
no-deconf.	EN	0.21 (0.02)	0.33 (0.01)	0.30 (0.01)	0.30 (0.02)	0.32 (0.02)	0.31 (0.02)	0.36 (0.02)
	RF	0.20 (0.02)	0.30 (0.01)	0.27 (0.02)	0.25 (0.03)	0.30 (0.02)	0.28 (0.01)	0.32 (0.03)
	SVR	0.23 (0.02)	0.33 (0.01)	0.29 (0.03)	0.32 (0.02)	0.33 (0.03)	0.33 (0.03)	0.37 (0.02)
	Ridge	0.19 (0.02)	0.33 (0.01)	0.33 (0.01)	0.32 (0.02)	0.34 (0.02)	0.31 (0.03)	0.36 (0.01)
	Lasso	0.18 (0.02)	0.33 (0.01)	0.27 (0.03)	0.24 (0.02)	0.31 (0.03)	0.30 (0.03)	0.33 (0.02)
deconf.	EN	0.15 (0.04)	0.13 (0.04)	0.22 (0.02)	0.14 (0.05)	0.14 (0.03)	0.08 (0.05)	0.16 (0.04)
	RF	0.13 (0.02)	0.13 (0.03)	0.17 (0.04)	0.11 (0.05)	0.10 (0.06)	0.07 (0.04)	0.12 (0.04)
	SVR	0.17 (0.01)	0.19 (0.03)	0.21 (0.03)	0.12 (0.03)	0.09 (0.04)	0.07 (0.03)	0.12 (0.05)
	Ridge	0.15 (0.03)	0.18 (0.03)	0.18 (0.01)	0.11 (0.04)	0.11 (0.05)	0.03 (0.06)	0.12 (0.04)
	Lasso	-0.01 (0.03)	0.00 (0.05)	0.03 (0.04)	0.00 (0.04)	0.00 (0.05)	-0.02 (0.03)	0.00 (0.03)

Supplementary Table S13. Regression performance for composite cognition in the concatenation approach in FSet B.

**FSet B**	**Global**	Unimodal			Multimodal				Unimodal						Multimodal							
	**Concat**	FC	SC	GMV	FC+ GMV	SC+ GMV	FC+ SC	FC+SC +GMV	FC		SC		GMV		FC+ GMV		SC+ GMV		FC+ SC		FC+SC+GMV	
Cond.	Algo.	MAE	MAE	MAE	MAE	MAE	MAE	MAE	R^2	folds > ref.	R^2	folds > ref.	R^2	folds > ref.	R^2	folds > ref.	R^2	folds > ref.	R^2	folds > ref.	R^2	folds > ref.
no-deconf.	EN	0.79 (0.03)	0.76 (0.04)	0.76 (0.04)	0.76 (0.04)	0.75 (0.04)	0.76 (0.04)	0.75 (0.04)	0.02 (0.04)	71%	0.11 (0.06)	97%	0.08 (0.06)	86%	0.09 (0.07)	91%	0.13 (0.06)	97%	0.10 (0.06)	93%	0.12 (0.07)	96%
	RF	0.79 (0.03)	0.77 (0.04)	0.78 (0.04)	0.77 (0.04)	0.75 (0.04)	0.76 (0.04)	0.75 (0.04)	0.02 (0.05)	68%	0.09 (0.06)	95%	0.06 (0.06)	87%	0.07 (0.06)	88%	0.11 (0.05)	96%	0.09 (0.06)	91%	0.11 (0.05)	97%
	SVR	0.78 (0.03)	0.77 (0.04)	0.77 (0.04)	0.76 (0.04)	0.75 (0.05)	0.77 (0.04)	0.76 (0.04)	0.03 (0.05)	74%	0.07 (0.06)	90%	0.06 (0.07)	81%	0.10 (0.07)	92%	0.12 (0.07)	91%	0.08 (0.06)	87%	0.11 (0.08)	92%
	Ridge	0.79 (0.04)	0.77 (0.04)	0.76 (0.04)	0.75 (0.04)	0.75 (0.04)	0.77 (0.04)	0.75 (0.05)	0.02 (0.06)	65%	0.10 (0.06)	94%	0.09 (0.05)	93%	0.10 (0.07)	91%	0.14 (0.07)	97%	0.07 (0.07)	89%	0.12 (0.08)	94%
	Lasso	0.79 (0.03)	0.77 (0.03)	0.78 (0.03)	0.77 (0.03)	0.75 (0.04)	0.76 (0.03)	0.75 (0.04)	0.02 (0.03)	75%	0.10 (0.04)	98%	0.05 (0.03)	94%	0.06 (0.04)	90%	0.11 (0.04)	99%	0.10 (0.04)	100%	0.12 (0.04)	100%
deconf.	EN	0.79 (0.03)	0.79 (0.03)	0.79 (0.03)	0.79 (0.03)	0.79 (0.03)	0.79 (0.03)	0.79 (0.03)	0.00 (0.01)	47%	0.00 (0.01)	43%	0.01 (0.02)	79%	0.00 (0.03)	58%	0.01 (0.02)	68%	0.00 (0.01)	54%	0.00 (0.02)	65%
	RF	0.80 (0.03)	0.79 (0.04)	0.79 (0.03)	0.79 (0.03)	0.79 (0.03)	0.80 (0.03)	0.79 (0.03)	-0.02 (0.04)	36%	0.00 (0.04)	60%	0.01 (0.04)	57%	0.00 (0.04)	46%	0.00 (0.04)	56%	-0.01 (0.04)	40%	0.00 (0.03)	57%
	SVR	0.79 (0.03)	0.79 (0.03)	0.79 (0.03)	0.79 (0.03)	0.79 (0.03)	0.79 (0.03)	0.79 (0.03)	-0.01 (0.02)	39%	0.00 (0.02)	41%	0.00 (0.04)	67%	0.00 (0.03)	58%	0.01 (0.03)	63%	0.00 (0.02)	45%	0.00 (0.03)	55%
	Ridge	0.79 (0.03)	0.79 (0.03)	0.79 (0.03)	0.79 (0.03)	0.79 (0.03)	0.79 (0.03)	0.79 (0.03)	0.00 (0.01)	55%	0.00 (0.01)	66%	0.01 (0.03)	75%	0.00 (0.03)	64%	0.01 (0.02)	77%	0.00 (0.02)	61%	0.01 (0.02)	68%
	Lasso	0.79 (0.03)	0.79 (0.03)	0.79 (0.03)	0.79 (0.03)	0.79 (0.03)	0.79 (0.03)	0.79 (0.03)	0.00 (0.01)	25%	0.00 (0.01)	9%	0.00 (0.01)	7%	0.00 (0.01)	14%	0.00 (0.00)	12%	0.00 (0.01)	12%	0.00 (0.01)	11%

Supplementary Table S14. Regression performance (correlation) for composite cognition in the concatenation approach in FSet B.

**FSet B**	**Global**	Unimodal			Multimodal			
	**Concat**	FC	SC	GMV	FC+ GMV	SC+ GMV	FC+ SC	FC+SC+GMV
Cond.	Algo.	r	r	r	r	r	r	r
no-deconf.	EN	0.16 (0.02)	0.34 (0.02)	0.28 (0.01)	0.30 (0.02)	0.36 (0.02)	0.32 (0.02)	0.35 (0.03)
	RF	0.16 (0.01)	0.31 (0.02)	0.24 (0.02)	0.26 (0.02)	0.34 (0.02)	0.31 (0.01)	0.34 (0.02)
	SVR	0.19 (0.02)	0.28 (0.02)	0.27 (0.01)	0.32 (0.01)	0.35 (0.02)	0.29 (0.01)	0.34 (0.01)
	Ridge	0.16 (0.02)	0.32 (0.01)	0.30 (0.01)	0.32 (0.02)	0.37 (0.01)	0.28 (0.03)	0.36 (0.01)
	Lasso	0.15 (0.02)	0.33 (0.03)	0.24 (0.03)	0.25 (0.03)	0.35 (0.02)	0.34 (0.03)	0.36 (0.02)
deconf.	EN	0.01 (0.05)	0.03 (0.04)	0.11 (0.01)	0.06 (0.04)	0.09 (0.03)	0.05 (0.02)	0.06 (0.03)
	RF	0.02 (0.03)	0.09 (0.05)	0.11 (0.02)	0.07 (0.02)	0.09 (0.04)	0.05 (0.05)	0.07 (0.05)
	SVR	0.01 (0.03)	0.04 (0.02)	0.09 (0.04)	0.08 (0.02)	0.11 (0.02)	0.04 (0.02)	0.09 (0.01)
	Ridge	0.00 (0.02)	0.03 (0.01)	0.10 (0.02)	0.07 (0.05)	0.11 (0.03)	0.01 (0.05)	0.08 (0.02)
	Lasso	0.01 (0.05)	0.00 (0.02)	-0.03 (0.01)	0.00 (0.04)	0.02 (0.03)	0.02 (0.06)	0.01 (0.03)

Supplementary Table S15. Regression performance for composite cognition in the stacking approach in FSet B.

**FSet B**	**Global**	Unimodal			Multimodal				Unimodal						Multimodal							
	**Stack**	FC	SC	GMV	FC+ GMV	SC+ GMV	FC+ SC	FC+SC +GMV	FC		SC		GMV		FC+ GMV		SC+ GMV		FC+ SC		FC+SC+GMV	
Cond.	Algo.	MAE	MAE	MAE	MAE	MAE	MAE	MAE	R^2	folds > ref.	R^2	folds > ref.	R^2	folds > ref.	R^2	folds > ref.	R^2	folds > ref.	R^2	folds > ref.	R^2	folds > ref.
no-deconf.	EN	0.79 (0.03)	0.76 (0.04)	0.76 (0.04)	0.77 (0.04)	0.75 (0.04)	0.77 (0.04)	0.75 (0.05)	0.02 (0.05)	70%	0.11 (0.06)	98%	0.08 (0.06)	88%	0.06 (0.07)	79%	0.11 (0.08)	89%	0.07 (0.07)	89%	0.10 (0.08)	89%
	RF	0.79 (0.03)	0.77 (0.04)	0.78 (0.04)	0.78 (0.04)	0.77 (0.04)	0.77 (0.04)	0.76 (0.04)	0.02 (0.05)	67%	0.09 (0.06)	95%	0.06 (0.05)	86%	0.03 (0.07)	68%	0.08 (0.07)	86%	0.07 (0.08)	81%	0.09 (0.08)	90%
	SVR	0.78 (0.03)	0.77 (0.04)	0.77 (0.04)	0.77 (0.04)	0.77 (0.05)	0.78 (0.05)	0.77 (0.05)	0.03 (0.05)	73%	0.07 (0.06)	90%	0.06 (0.06)	87%	0.06 (0.08)	77%	0.08 (0.08)	83%	0.06 (0.08)	79%	0.09 (0.08)	87%
	Ridge	0.79 (0.03)	0.77 (0.04)	0.76 (0.04)	0.77 (0.04)	0.76 (0.04)	0.78 (0.04)	0.76 (0.04)	0.02 (0.05)	66%	0.10 (0.06)	94%	0.09 (0.05)	93%	0.07 (0.07)	82%	0.10 (0.08)	91%	0.06 (0.08)	79%	0.10 (0.08)	94%
	Lasso	0.79 (0.03)	0.77 (0.03)	0.78 (0.03)	0.78 (0.04)	0.76 (0.05)	0.77 (0.04)	0.76 (0.04)	0.02 (0.03)	75%	0.10 (0.04)	98%	0.05 (0.03)	94%	0.04 (0.06)	73%	0.10 (0.07)	91%	0.08 (0.07)	89%	0.10 (0.08)	89%
deconf.	EN	0.79 (0.03)	0.79 (0.03)	0.79 (0.03)	0.80 (0.03)	0.80 (0.04)	0.80 (0.03)	0.80 (0.03)	0.00 (0.02)	54%	0.00 (0.01)	46%	0.01 (0.02)	71%	-0.01 (0.04)	44%	0.00 (0.04)	44%	-0.02 (0.04)	32%	-0.01 (0.04)	43%
	RF	0.80 (0.03)	0.79 (0.04)	0.79 (0.03)	0.80 (0.04)	0.80 (0.04)	0.80 (0.03)	0.80 (0.03)	-0.02 (0.04)	35%	0.00 (0.03)	60%	0.01 (0.04)	60%	-0.02 (0.04)	38%	-0.02 (0.05)	33%	-0.03 (0.04)	31%	-0.02 (0.04)	31%
	SVR	0.79 (0.03)	0.79 (0.03)	0.79 (0.03)	0.80 (0.03)	0.80 (0.03)	0.81 (0.03)	0.81 (0.03)	-0.01 (0.02)	39%	0.00 (0.02)	41%	0.00 (0.03)	61%	-0.02 (0.06)	35%	-0.02 (0.06)	28%	-0.04 (0.05)	21%	-0.03 (0.05)	31%
	Ridge	0.79 (0.03)	0.79 (0.03)	0.79 (0.03)	0.80 (0.04)	0.80 (0.03)	0.81 (0.03)	0.80 (0.03)	0.00 (0.01)	54%	0.00 (0.01)	66%	0.01 (0.03)	76%	-0.02 (0.05)	41%	-0.01 (0.05)	45%	-0.03 (0.04)	21%	-0.02 (0.05)	41%
	Lasso	0.79 (0.03)	0.79 (0.03)	0.79 (0.03)	0.80 (0.03)	0.80 (0.03)	0.80 (0.03)	0.80 (0.03)	0.00 (0.01)	24%	0.00 (0.01)	13%	0.00 (0.01)	4%	-0.02 (0.04)	16%	-0.01 (0.04)	9%	-0.02 (0.04)	16%	-0.02 (0.05)	16%

Supplementary Table S16. Regression performance (correlation) for composite cognition in the stacking approach in FSet B.

**FSet B**	**Global**	Unimodal			Multimodal			
	**Stack**	FC	SC	GMV	FC+ GMV	SC+ GMV	FC+ SC	FC+SC+GMV
Cond.	Algo.	r	r	r	r	r	r	r
no-deconf.	EN	0.16 (0.03)	0.33 (0.02)	0.30 (0.02)	0.27 (0.03)	0.33 (0.03)	0.28 (0.02)	0.34 (0.02)
	RF	0.15 (0.03)	0.31 (0.04)	0.27 (0.01)	0.22 (0.01)	0.28 (0.02)	0.27 (0.04)	0.31 (0.02)
	SVR	0.18 (0.02)	0.28 (0.01)	0.29 (0.01)	0.28 (0.03)	0.30 (0.06)	0.26 (0.03)	0.32 (0.04)
	Ridge	0.15 (0.02)	0.32 (0.01)	0.33 (0.01)	0.29 (0.02)	0.32 (0.02)	0.27 (0.03)	0.34 (0.02)
	Lasso	0.16 (0.03)	0.33 (0.03)	0.27 (0.03)	0.24 (0.04)	0.32 (0.06)	0.29 (0.03)	0.33 (0.05)
deconf.	EN	0.03 (0.03)	0.09 (0.03)	0.20 (0.03)	0.12 (0.05)	0.13 (0.04)	0.02 (0.06)	0.13 (0.03)
	RF	0.04 (0.03)	0.17 (0.03)	0.17 (0.03)	0.08 (0.07)	0.09 (0.08)	0.04 (0.03)	0.09 (0.08)
	SVR	0.13 (0.03)	0.10 (0.03)	0.19 (0.05)	0.06 (0.06)	0.07 (0.10)	-0.03 (0.03)	0.05 (0.06)
	Ridge	0.07 (0.03)	0.14 (0.02)	0.19 (0.03)	0.10 (0.03)	0.10 (0.06)	-0.01 (0.06)	0.10 (0.04)
	Lasso	0.02 (0.03)	0.04 (0.03)	0.03 (0.04)	0.01 (0.05)	0.00 (0.05)	0.01 (0.04)	0.01 (0.04)

Supplementary Table S17. Multimodal bonus in the concatenation approach for FSet A across targets.

**FSet A**	**Concat**	Global Cognition								Component 1								Component 2							
		FC+ GMV		SC+ GMV		FC+SC		FC+SC+GMV		FC+ GMV		SC+ GMV		FC+SC		FC+SC+GMV		FC+ GMV		SC+ GMV		FC+SC		FC+SC+GMV	
Cond.	Algo.	B_{all}	B_{best}	B_{all}	B_{best}	B_{all}	B_{best}	B_{all}	B_{best}	B_{all}	B_{best}	B_{all}	B_{best}	B_{all}	B_{best}	B_{all}	B_{best}	B_{all}	B_{best}	B_{all}	B_{best}	B_{all}	B_{best}	B_{all}	B_{best}
no-	EN	0.01	-0.02	0.06	0.02	0.04	0.00	0.06	0.03	0.02	0.01	0.03	0.02	0.05	0.01	0.03	0.04	-0.01	-0.04	0.02	0.00	0.01	-0.01	0.01	-0.01
deconf.	RF	0.01	-0.02	0.04	0.01	0.03	0.00	0.05	0.02	0.02	0.02	0.03	0.02	0.04	0.02	0.02	0.03	-0.02	-0.04	0.03	0.00	0.01	-0.01	0.02	-0.01
	SVR	0.02	-0.01	0.06	0.03	0.04	0.00	0.06	0.02	0.03	0.02	0.03	0.02	0.05	0.02	0.03	0.05	-0.01	-0.03	0.03	0.01	0.01	-0.01	0.01	-0.01
	Ridge	0.01	-0.02	0.05	0.02	0.02	-0.01	0.05	0.02	0.03	0.03	0.03	0.02	0.06	0.03	0.03	0.06	-0.01	-0.03	0.03	0.00	0.02	-0.01	0.02	0.00
	Lasso	0.00	-0.04	0.06	0.01	0.05	0.01	0.06	0.02	0.01	-0.01	0.03	0.02	0.03	0.01	0.02	0.02	-0.01	-0.04	0.02	0.00	0.02	-0.01	0.02	0.00
deconf.	EN	0.00	-0.01	0.00	-0.01	0.00	-0.01	0.00	0.00	0.00	-0.01	0.00	0.00	0.00	-0.01	0.00	0.00	0.00	-0.01	0.00	0.00	0.00	-0.01	0.00	-0.01
	RF	0.01	0.00	0.01	0.00	0.00	-0.01	0.01	0.00	0.01	0.00	-0.01	-0.02	0.01	-0.01	0.01	-0.01	-0.01	-0.01	0.00	0.00	0.00	-0.01	0.00	-0.01
	SVR	0.00	0.00	0.01	0.00	-0.01	-0.01	0.00	0.00	0.01	0.00	0.01	0.00	0.01	-0.01	-0.01	0.00	-0.01	-0.02	0.01	0.00	-0.01	-0.02	-0.01	-0.02
	Ridge	0.00	-0.01	0.00	0.00	0.00	-0.01	0.00	-0.01	0.00	0.00	0.00	0.00	0.01	0.00	0.00	0.00	0.00	-0.01	0.01	0.00	0.00	0.00	0.00	0.00
	Lasso	0.00	0.00	0.00	0.00	0.00	0.00	0.00	0.00	0.00	0.00	0.00	0.00	0.00	0.00	0.00	-0.01	0.00	0.00	0.00	0.00	0.00	0.00	0.00	0.00

Supplementary Table S18. Multimodal bonus in the concatenation approach for FSet B across targets.

**FSet B**	**Concat**	Global Cognition								Component 1								Component 2							
		FC+ GMV		SC+ GMV		FC+SC		FC+SC+GMV		FC+ GMV		SC+ GMV		FC+SC		FC+SC+GMV		FC+ GMV		SC+ GMV		FC+SC		FC+SC+GMV	
Cond.	Algo.	B_{all}	B_{best}	B_{all}	B_{best}	B_{all}	B_{best}	B_{all}	B_{best}	B_{all}	B_{best}	B_{all}	B_{best}	B_{all}	B_{best}	B_{all}	B_{best}	B_{all}	B_{best}	B_{all}	B_{best}	B_{all}	B_{best}	B_{all}	B_{best}
no-	EN	0.02	-0.02	0.06	0.02	0.03	-0.01	0.05	0.01	0.02	0.00	0.03	0.00	0.03	-0.01	0.01	0.01	-0.01	-0.03	0.03	0.01	0.00	-0.01	0.01	-0.01
deconf.	RF	0.01	-0.02	0.05	0.02	0.03	0.00	0.05	0.01	0.02	0.00	0.04	0.02	0.04	0.00	0.02	0.02	0.00	-0.02	0.03	0.01	0.02	0.00	0.02	0.00
	SVR	0.04	0.02	0.06	0.04	0.03	0.01	0.06	0.04	0.02	0.01	0.03	0.02	0.03	-0.01	0.00	0.02	-0.01	-0.02	0.02	0.01	0.01	-0.01	0.01	0.00
	Ridge	0.03	0.00	0.07	0.04	0.01	-0.03	0.06	0.03	0.02	0.00	0.03	0.01	0.03	-0.01	0.00	0.01	-0.01	-0.01	0.02	0.01	0.01	0.00	0.02	0.01
	Lasso	0.00	-0.04	0.06	0.02	0.05	0.01	0.06	0.02	0.00	-0.03	0.04	0.01	0.04	0.00	0.03	0.01	0.00	0.00	0.01	0.01	0.00	0.00	0.01	0.00
deconf.	EN	0.00	-0.01	0.00	0.00	0.00	-0.01	0.00	-0.01	0.01	0.00	0.01	0.00	0.01	-0.01	0.00	0.00	0.00	0.00	0.00	0.00	-0.01	-0.01	-0.01	-0.01
	RF	0.00	-0.01	0.01	0.00	-0.01	-0.01	0.00	-0.01	0.00	-0.01	0.01	0.01	0.01	0.00	0.01	0.00	0.00	-0.02	0.01	0.00	0.00	-0.01	0.00	-0.01
	SVR	0.00	0.00	0.01	0.01	0.00	-0.01	0.01	0.00	0.00	0.00	0.01	0.00	0.01	-0.01	0.00	0.00	0.00	-0.01	0.01	0.00	0.00	-0.01	0.00	0.00
	Ridge	0.00	-0.01	0.01	0.00	-0.01	-0.01	0.00	0.00	0.00	0.00	0.00	0.00	0.00	0.00	0.00	0.00	0.00	0.00	0.00	0.00	0.00	-0.01	0.00	0.00
	Lasso	0.00	0.00	0.00	0.00	0.00	0.00	0.00	0.00	0.00	0.00	0.00	0.00	0.00	0.00	0.00	0.00	0.00	0.00	0.00	0.00	0.00	0.00	0.00	0.00

Supplementary Table S19. Multimodal bonus in the stacking approach for FSet A across targets.

**FSet A**	**Stack**	Global Cognition								Component 1								Component 2							
		FC+ GMV		SC+ GMV		FC+SC		FC+SC+GMV		FC+ GMV		SC+ GMV		FC+SC		FC+SC+GMV		FC+ GMV		SC+ GMV		FC+SC		FC+SC+GMV	
Cond.	Algo.	B_{all}	B_{best}	B_{all}	B_{best}	B_{all}	B_{best}	B_{all}	B_{best}	B_{all}	B_{best}	B_{all}	B_{best}	B_{all}	B_{best}	B_{all}	B_{best}	B_{all}	B_{best}	B_{all}	B_{best}	B_{all}	B_{best}	B_{all}	B_{best}
no-	EN	0.00	-0.03	0.02	-0.01	0.01	-0.02	0.04	0.01	0.01	0.00	0.01	0.00	0.02	0.01	0.04	0.03	-0.03	-0.05	-0.01	-0.04	0.00	-0.02	0.00	-0.03
deconf.	RF	-0.02	-0.05	0.02	-0.01	0.01	-0.02	0.03	0.00	0.01	0.00	0.00	-0.01	0.00	0.00	0.02	0.01	-0.04	-0.05	-0.01	-0.04	-0.02	-0.05	-0.02	-0.04
	SVR	0.02	-0.01	0.03	0.00	0.03	0.00	0.05	0.02	0.01	0.01	0.02	0.01	0.02	0.02	0.04	0.04	-0.03	-0.05	-0.01	-0.03	-0.01	-0.03	-0.01	-0.03
	Ridge	0.01	-0.02	0.04	0.01	0.02	-0.01	0.05	0.02	0.02	0.02	0.01	0.01	0.03	0.03	0.04	0.04	-0.03	-0.06	0.00	-0.03	-0.01	-0.03	0.00	-0.03
	Lasso	-0.02	-0.06	0.03	-0.01	0.02	-0.02	0.04	-0.01	-0.01	-0.02	0.01	0.00	0.02	0.00	0.03	0.01	-0.02	-0.05	0.00	-0.03	0.00	-0.03	0.00	-0.02
deconf.	EN	-0.01	-0.02	-0.01	-0.02	-0.02	-0.03	-0.01	-0.02	-0.01	-0.02	-0.02	-0.02	-0.02	-0.03	-0.01	-0.02	-0.02	-0.02	-0.02	-0.03	-0.01	-0.01	-0.02	-0.02
	RF	-0.01	-0.02	-0.01	-0.02	-0.02	-0.03	-0.01	-0.02	-0.01	-0.03	-0.02	-0.03	-0.01	-0.03	-0.02	-0.03	-0.01	-0.02	-0.01	-0.02	-0.01	-0.02	-0.01	-0.02
	SVR	-0.02	-0.02	-0.02	-0.03	-0.02	-0.02	-0.01	-0.02	-0.02	-0.03	-0.02	-0.03	-0.02	-0.03	-0.02	-0.03	-0.01	-0.02	-0.02	-0.03	-0.02	-0.03	-0.01	-0.02
	Ridge	-0.02	-0.03	-0.02	-0.03	-0.03	-0.04	-0.02	-0.03	-0.02	-0.02	-0.02	-0.02	-0.01	-0.02	-0.01	-0.02	-0.02	-0.02	-0.03	-0.03	-0.01	-0.01	-0.01	-0.02
	Lasso	-0.01	-0.01	0.00	0.00	0.00	0.00	-0.01	-0.01	-0.01	-0.01	-0.01	-0.01	-0.01	-0.01	-0.01	-0.02	0.00	-0.01	0.00	0.00	0.00	-0.01	-0.01	-0.01

Supplementary Table S20. Multimodal bonus in the stacking approach for FSet B across targets.

**FSet B**	**Stack**	Global Cognition								Component 1								Component 2							
		FC+ GMV		SC+ GMV		FC+SC		FC+SC+GMV		FC+ GMV		SC+ GMV		FC+SC		FC+SC+GMV		FC+ GMV		SC+ GMV		FC+SC		FC+SC+GMV	
Cond.	Algo.	B_{all}	B_{best}	B_{all}	B_{best}	B_{all}	B_{best}	B_{all}	B_{best}	B_{all}	B_{best}	B_{all}	B_{best}	B_{all}	B_{best}	B_{all}	B_{best}	B_{all}	B_{best}	B_{all}	B_{best}	B_{all}	B_{best}	B_{all}	B_{best}
no-deconf.	EN	-0.01	-0.05	0.00	-0.01	0.03	0.00	0.04	-0.04	-0.01	-0.03	-0.01	-0.01	0.02	-0.01	0.02	-0.03	-0.02	-0.04	-0.01	-0.02	0.00	-0.02	0.00	-0.02
	RF	-0.03	-0.06	0.01	-0.01	0.03	-0.01	0.02	-0.02	-0.01	-0.03	0.01	0.01	0.02	0.00	0.02	-0.01	-0.04	-0.04	-0.02	-0.04	-0.02	-0.04	-0.02	-0.04
	SVR	0.00	-0.02	0.00	0.01	0.03	0.01	0.03	-0.02	0.00	-0.01	0.00	0.01	0.02	0.01	0.02	-0.01	-0.04	-0.05	-0.02	-0.02	-0.01	-0.02	-0.01	-0.03
	Ridge	0.00	-0.03	-0.01	0.00	0.03	0.00	0.03	-0.04	0.01	-0.01	-0.01	0.00	0.02	0.01	0.02	-0.03	-0.03	-0.04	-0.01	-0.02	-0.01	-0.02	-0.01	-0.02
	Lasso	-0.01	-0.05	0.03	0.00	0.04	0.00	0.04	-0.01	-0.03	-0.06	0.01	-0.01	0.02	-0.01	0.02	-0.02	-0.02	-0.03	-0.03	-0.03	-0.03	-0.02	-0.02	-0.03
deconf.	EN	-0.01	-0.02	-0.01	-0.02	-0.02	-0.03	-0.01	-0.02	-0.02	-0.02	-0.01	-0.02	-0.02	-0.03	-0.02	-0.03	-0.01	-0.02	-0.02	-0.02	-0.01	-0.01	-0.01	-0.02
	RF	-0.02	-0.02	-0.02	-0.03	-0.02	-0.03	-0.02	-0.03	-0.02	-0.02	-0.02	-0.02	-0.02	-0.02	-0.02	-0.02	-0.01	-0.03	-0.01	-0.03	-0.01	-0.02	-0.01	-0.02
	SVR	-0.02	-0.02	-0.02	-0.02	-0.03	-0.04	-0.02	-0.03	-0.02	-0.03	-0.02	-0.03	-0.03	-0.04	-0.02	-0.03	-0.03	-0.03	-0.03	-0.03	-0.02	-0.03	-0.03	-0.03
	Ridge	-0.02	-0.02	-0.02	-0.02	-0.03	-0.04	-0.02	-0.03	-0.02	-0.02	-0.03	-0.03	-0.03	-0.03	-0.03	-0.03	-0.02	-0.02	-0.03	-0.03	-0.01	-0.01	-0.01	-0.02
	Lasso	-0.02	-0.02	-0.01	-0.01	-0.01	-0.02	-0.02	-0.02	-0.01	-0.01	0.00	-0.01	-0.01	-0.01	-0.01	-0.01	0.00	0.00	-0.01	-0.01	-0.01	-0.01	-0.01	-0.01

Supplementary Table S21. Regression performance for composite cognition in the concatenation approach in FSet A including confounders.

**FSet A**	**Global**	EN	RF	SVR	Ridge	Lasso	EN		RF		SVR		Ridge		Lasso		EN	RF	SVR	Ridge	Lasso
Cond.	**Concat**	MAE	MAE	MAE	MAE	MAE	R^2	folds > ref.	R^2	folds > ref.	R^2	folds > ref.	R^2	folds > ref.	R^2	folds > ref.	r	r	r	r	r
no-deconf.	FC	0.78 (0.03)	0.78 (0.03)	0.77 (0.04)	0.78 (0.03)	0.78 (0.03)	0.04 (0.06)	76%	0.04 (0.05)	79%	0.04 (0.06)	74%	0.03 (0.06)	76%	0.03 (0.03)	83%	0.21 (0.04)	0.20 (0.03)	0.22 (0.02)	0.19 (0.04)	0.18 (0.04)
	SC	0.76 (0.04)	0.76 (0.04)	0.75 (0.04)	0.76 (0.05)	0.76 (0.03)	0.11 (0.07)	93%	0.09 (0.05)	96%	0.10 (0.07)	90%	0.10 (0.08)	90%	0.10 (0.05)	96%	0.33 (0.01)	0.31 (0.01)	0.33 (0.01)	0.33 (0.01)	0.33 (0.01)
	GMV	0.76 (0.04)	0.77 (0.03)	0.77 (0.04)	0.76 (0.04)	0.78 (0.03)	0.08 (0.06)	89%	0.06 (0.05)	89%	0.06 (0.07)	86%	0.09 (0.05)	93%	0.05 (0.03)	94%	0.28 (0.02)	0.25 (0.01)	0.27 (0.02)	0.30 (0.01)	0.24 (0.03)
	CF	0.65 (0.05)	0.65 (0.05)	0.64 (0.05)	0.64 (0.05)	0.65 (0.04)	0.34 (0.08)	100%	0.32 (0.10)	100%	0.34 (0.09)	100%	0.34 (0.09)	100%	0.32 (0.07)	100%	0.58 (0.00)	0.57 (0.01)	0.58 (0.01)	0.58 (0.00)	0.58 (0.00)
	CF+FC	0.66 (0.04)	0.67 (0.05)	0.74 (0.05)	0.74 (0.04)	0.66 (0.04)	0.32 (0.07)	100%	0.29 (0.09)	100%	0.12 (0.08)	94%	0.13 (0.07)	96%	0.32 (0.07)	100%	0.57 (0.01)	0.54 (0.01)	0.36 (0.04)	0.36 (0.04)	0.58 (0.01)
	CF+SC	0.66 (0.04)	0.67 (0.05)	0.73 (0.05)	0.72 (0.05)	0.66 (0.04)	0.32 (0.07)	100%	0.29 (0.09)	100%	0.15 (0.08)	97%	0.18 (0.07)	98%	0.32 (0.07)	100%	0.57 (0.00)	0.54 (0.01)	0.40 (0.01)	0.43 (0.01)	0.58 (0.00)
	CF+ GMV	0.65 (0.04)	0.67 (0.05)	0.70 (0.04)	0.69 (0.05)	0.66 (0.04)	0.32 (0.07)	100%	0.30 (0.08)	100%	0.22 (0.09)	98%	0.24 (0.11)	99%	0.32 (0.07)	100%	0.57 (0.01)	0.55 (0.01)	0.47 (0.01)	0.50 (0.02)	0.58 (0.01)
	CF+FC+ GMV	0.66 (0.04)	0.67 (0.05)	0.72 (0.04)	0.72 (0.04)	0.66 (0.04)	0.31 (0.07)	100%	0.30 (0.09)	100%	0.16 (0.09)	95%	0.16 (0.09)	97%	0.31 (0.07)	100%	0.56 (0.02)	0.55 (0.02)	0.41 (0.02)	0.41 (0.02)	0.57 (0.01)
	CF+SC+ GMV	0.66 (0.05)	0.67 (0.05)	0.72 (0.05)	0.71 (0.05)	0.66 (0.04)	0.31 (0.07)	100%	0.29 (0.08)	100%	0.18 (0.09)	96%	0.20 (0.08)	99%	0.31 (0.07)	100%	0.57 (0.01)	0.55 (0.00)	0.43 (0.01)	0.44 (0.01)	0.57 (0.01)
	CF+FC+ SC	0.66 (0.04)	0.67 (0.05)	0.73 (0.04)	0.73 (0.05	0.66 (0.04)	0.31 (0.07)	100%	0.29 (0.08)	100%	0.15 (0.08)	97%	0.15 (0.08)	94%	0.31 (0.07)	100%	0.57 (0.01)	0.54 (0.01)	0.39 (0.01)	0.39 (0.02)	0.57 (0.00)
	CF+FC+ SC+ GMV	0.66 (0.04)	0.67 (0.05)	0.72 (0.05)	0.72 (0.05)	0.66 (0.04)	0.31 (0.07)	100%	0.30 (0.08)	100%	0.17 (0.08)	98%	0.18 (0.09)	96%	0.31 (0.07)	100%	0.56 (0.01)	0.55 (0.01)	0.42 (0.01)	0.42 (0.02)	0.57 (0.01)

Supplementary Table S22. Regression performance for composite cognition in the stacking approach in FSet A with confounders.

**FSet A**	**Global**	EN	RF	SVR	Ridge	Lasso	EN		RF		SVR		Ridge		Lasso		EN	RF	SVR	Ridge	Lasso
Cond.	**Stack**	MAE	MAE	MAE	MAE	MAE	R^2	folds > ref.	R^2	folds > ref.	R^2	folds > ref.	R^2	folds > ref.	R^2	folds > ref.	r	r	r	r	r
no-deconf.	FC	0.78 (0.03)	0.78 (0.03)	0.77 (0.04)	0.78 (0.04)	0.78 (0.03)	0.04 (0.05)	78%	0.04 (0.05)	76%	0.04 (0.06)	75%	0.03 (0.06)	74%	0.03 (0.03)	83%	0.21 (0.02)	0.20 (0.02)	0.23 (0.02)	0.19 (0.02)	0.18 (0.02)
	SC	0.76 (0.04)	0.76 (0.04)	0.75 (0.04)	0.76 (0.05)	0.76 (0.03)	0.11 (0.07)	94%	0.09 (0.06)	95%	0.10 (0.07)	90%	0.10 (0.08)	90%	0.10 (0.05)	96%	0.33 (0.01)	0.30 (0.01)	0.33 (0.01)	0.33 (0.01)	0.33 (0.01)
	GMV	0.76 (0.04)	0.78 (0.04)	0.77 (0.04)	0.76 (0.04)	0.78 (0.03)	0.08 (0.06)	89%	0.06 (0.06)	87%	0.06 (0.07)	82%	0.09 (0.05)	93%	0.05 (0.03)	94%	0.30 (0.01)	0.27 (0.02)	0.29 (0.03)	0.33 (0.01)	0.27 (0.03)
	CF	0.65 (0.05)	0.65 (0.05)	0.64 (0.05)	0.64 (0.05)	0.65 (0.04)	0.34 (0.08)	100%	0.32 (0.10)	100%	0.34 (0.09)	100%	0.34 (0.09)	100%	0.32 (0.07)	100%	0.59 (0.00)	0.57 (0.00)	0.59 (0.00)	0.59 (0.00)	0.59 (0.00)
	CF+FC	0.66 (0.05)	0.67 (0.06)	0.66 (0.05)	0.66 (0.06)	0.66 (0.05)	0.31 (0.10)	100%	0.29 (0.10	99%	0.31 (0.10)	100%	0.31 (0.10)	100%	0.31 (0.10)	100%	0.57 (0.01)	0.55 (0.01)	0.57 (0.00)	0.57 (0.01)	0.57 (0.00)
	CF+SC	0.66 (0.06)	0.66 (0.05)	0.66 (0.06)	0.66 (0.06)	0.66 (0.06)	0.31 (0.10)	100%	0.29 (0.10)	100%	0.30 (0.11)	100%	0.31 (0.10)	100%	0.32 (0.10)	100%	0.56 (0.01)	0.55 (0.01)	0.56 (0.01)	0.57 (0.01)	0.57 (0.01)
	CF+ GMV	0.65 (0.06)	0.67 (0.06)	0.66 (0.06)	0.65 (0.05)	0.66 (0.06)	0.32 (0.10)	100%	0.29 (0.10)	99%	0.31 (0.10)	100%	0.32 (0.10)	100%	0.31 (0.10)	100%	0.58 (0.01)	0.55 (0.01)	0.57 (0.01)	0.57 (0.00)	0.57 (0.00)
	CF+FC+ GMV	0.65 (0.06)	0.67 (0.06)	0.65 (0.05)	0.65 (0.06)	0.66 (0.05)	0.32 (0.10)	100%	0.29 (0.10)	100%	0.32 (0.10)	100%	0.32 (0.10)	100%	0.31 (0.10)	100%	0.58 (0.01)	0.55 (0.01)	0.57 (0.01)	0.57 (0.01)	0.57 (0.01)
	CF+SC+ GMV	0.65 (0.06)	0.66 (0.05)	0.66 (0.06)	0.65 (0.06)	0.66 (0.05)	0.32 (0.10)	100%	0.29 (0.10)	100%	0.31 (0.10)	100%	0.32 (0.10)	100%	0.32 (0.10)	100%	0.57 (0.01)	0.55 (0.01)	0.56 (0.01)	0.57 (0.01)	0.57 (0.01)
	CF+FC+ SC	0.66 (0.05)	0.66 (0.06)	0.66 (0.05)	0.66 (0.06)	0.66 (0.05)	0.31 (0.10)	100%	0.29 (0.10)	100%	0.30 (0.10)	100%	0.31 (0.10)	100%	0.31 (0.10)	100%	0.57 (0.01	0.55 (0.01)	0.56 (0.01)	0.56 (0.01)	0.57 (0.01)
	CF+FC+SC +GMV	0.65 (0.05)	0.66 (0.06)	0.66 (0.06)	0.65 (0.06)	0.66 (0.06)	0.32 (0.10)	100%	0.29 (0.10)	100%	0.31 (0.10)	100%	0.32 (0.10)	100%	0.31 (0.10)	100%	0.57 (0.01)	0.55 (0.01)	0.57 (0.01)	0.57 (0.01)	0.57 (0.01)

Supplementary Table S23. Regression performance for composite cognition in the concatenation approach in FSet B with confounders.

**FSet B**	**Global**	EN	RF	SVR	Ridge	Lasso	EN		RF		SVR		Ridge		Lasso		EN	RF	SVR	Ridge	Lasso
Cond.	**Concat**	MAE	MAE	MAE	MAE	MAE	R^2	folds > ref.	R^2	folds > ref.	R^2	folds > ref.	R^2	folds > ref.	R^2	folds > ref.	r	r	r	r	r
no-deconf.	FC	0.79 (0.03)	0.79 (0.03)	0.78 (0.03)	0.79 (0.04)	0.79 (0.03)	0.02 (0.04)	71%	0.02 (0.05)	68%	0.03 (0.05)	74%	0.02 (0.06)	65%	0.02 (0.03)	75%	0.16 (0.02)	0.16 (0.01)	0.19 (0.02)	0.16 (0.02)	0.15 (0.02)
	SC	0.76 (0.04)	0.77 (0.04)	0.77 (0.04)	0.77 (0.04)	0.77 (0.03)	0.11 (0.06)	97%	0.09 (0.06)	95%	0.07 (0.06)	90%	0.10 (0.06)	94%	0.10 (0.04)	98%	0.34 (0.02)	0.31 (0.02)	0.28 (0.02)	0.32 (0.01)	0.33 (0.03)
	GMV	0.76 (0.04)	0.78 (0.04)	0.77 (0.04)	0.76 (0.04)	0.78 (0.03)	0.08 (0.06)	86%	0.06 (0.06)	87%	0.06 (0.07)	81%	0.09 (0.05)	93%	0.05 (0.03)	94%	0.28 (0.01)	0.24 (0.02)	0.27 (0.01)	0.30 (0.01)	0.24 (0.03)
	CF	0.65 (0.05)	0.65 (0.05)	0.64 (0.05)	0.64 (0.05)	0.65 (0.04)	0.34 (0.08)	100%	0.32 (0.10)	100%	0.34 (0.09)	100%	0.34 (0.09)	100%	0.32 (0.07)	100%	0.58 (0.01)	0.57 (0.01)	0.58 (0.00)	0.58 (0.00)	0.58 (0.00)
	CF+FC	0.65 (0.04)	0.67 (0.05)	0.72 (0.05)	0.74 (0.05)	0.65 (0.04)	0.32 (0.07)	100%	0.29 (0.09)	100%	0.16 (0.08)	97%	0.12 (0.10)	89%	0.32 (0.07)	100%	0.57 (0.01)	0.54 (0.01)	0.41 (0.02)	0.37 (0.03)	0.58 (0.01)
	CF+SC	0.66 (0.04)	0.67 (0.05)	0.73 (0.05)	0.73 (0.05)	0.66 (0.04)	0.32 (0.07)	100%	0.30 (0.09)	100%	0.18 (0.07)	100%	0.17 (0.07)	99%	0.32 (0.07)	100%	0.58 (0.01)	0.55 (0.01)	0.42 (0.01)	0.42 (0.01)	0.58 (0.01)
	CF+ GMV	0.66 (0.05)	0.67 (0.05)	0.70 (0.05)	0.69 (0.05)	0.66 (0.04)	0.32 (0.07)	100%	0.30 (0.08)	100%	0.22 (0.09)	97%	0.24 (0.11)	99%	0.32 (0.07)	100%	0.57 (0.02)	0.55 (0.01)	0.47 (0.01)	0.50 (0.02)	0.58 (0.01)
	CF+FC+ GMV	0.66 (0.04)	0.67 (0.05)	0.71 (0.05)	0.72 (0.04)	0.66 (0.04)	0.32 (0.07)	100%	0.29 (0.08)	100%	0.18 (0.09)	95%	0.18 (0.08)	98%	0.32 (0.07)	100%	0.57 (0.02)	0.54 (0.00)	0.44 (0.02)	0.42 (0.05)	0.58 (0.01)
	CF+SC+ GMV	0.66 (0.04)	0.67 (0.05)	0.73 (0.05)	0.72 (0.04)	0.66 (0.04)	0.32 (0.07)	100%	0.30 (0.09)	100%	0.17 (0.10)	93%	0.21 (0.07)	100%	0.32 (0.07)	100%	0.58 (0.01)	0.55 (0.01)	0.43 (0.01)	0.46 (0.01)	0.58 (0.01)
	CF+FC+ SC	0.65 (0.04)	0.68 (0.05)	0.75 (0.05)	0.73 (0.05)	0.65 (0.04)	0.32 (0.07)	100%	0.29 (0.09)	100%	0.13 (0.08)	92%	0.16 (0.07)	96%	0.32 (0.07)	100%	0.58 (0.01)	0.54 (0.01)	0.36 (0.01)	0.40 (0.01)	0.58 (0.00)
	CF+FC+SC +GMV	0.66 (0.04)	0.67 (0.05)	0.73 (0.05)	0.72 (0.05)	0.66 (0.04)	0.32 (0.07)	100%	0.30 (0.08)	100%	0.16 (0.09)	94%	0.19 (0.08)	97%	0.32 (0.07)	100%	0.58 (0.01)	0.55 (0.00)	0.41 (0.02)	0.43 (0.01)	0.58 (0.01)

Supplementary Table S24. Regression performance for composite cognition in the stacking approach in FSet B with confounders.

**FSet B**	**Global**	EN	RF	SVR	Ridge	Lasso	EN		RF		SVR		Ridge		Lasso		EN	RF	SVR	Ridge	Lasso
Cond.	**Stack**	MAE	MAE	MAE	MAE	MAE	R^2	folds > ref.	R^2	folds > ref.	R^2	folds > ref.	R^2	folds > ref.	R^2	folds > ref.	r	r	r	r	r
no-deconf.	FC	0.79 (0.03)	0.79 (0.03)	0.78 (0.03)	0.79 (0.03)	0.79 (0.03)	0.02 (0.05)	70%	0.02 (0.05)	67%	0.03 (0.05)	73%	0.02 (0.05)	66%	0.02 (0.03)	75%	0.16 (0.03)	0.15 (0.03)	0.18 (0.02)	0.15 (0.02)	0.16 (0.03)
	SC	0.76 (0.04)	0.77 (0.04)	0.77 (0.04)	0.77 (0.04)	0.77 (0.03)	0.11 (0.06)	98%	0.09 (0.06)	95%	0.07 (0.06)	90%	0.10 (0.06)	94%	0.10 (0.04)	98%	0.33 (0.02)	0.31 (0.04)	0.28 (0.01)	0.32 (0.01)	0.33 (0.03)
	GMV	0.76 (0.04)	0.78 (0.04)	0.77 (0.04)	0.76 (0.04)	0.78 (0.03)	0.08 (0.06)	88%	0.06 (0.05)	86%	0.06 (0.06)	87%	0.09 (0.05)	93%	0.05 (0.03)	94%	0.30 (0.02)	0.27 (0.01)	0.29 (0.01)	0.33 (0.01)	0.27 (0.03)
	CF	0.65 (0.05)	0.65 (0.05)	0.64 (0.05)	0.64 (0.05)	0.65 (0.04)	0.34 (0.08)	100%	0.32 (0.10)	100%	0.34 (0.09)	100%	0.34 (0.09)	100%	0.32 (0.07)	100%	0.59 (0.00)	0.58 (0.00)	0.59 (0.00)	0.59 (0.00)	0.59 (0.00)
	CF+FC	0.66 (0.05)	0.66 (0.05)	0.66 (0.05)	0.66 (0.05)	0.66 (0.05)	0.31 (0.10)	100%	0.29 (0.10)	100%	0.30 (0.10)	100%	0.30 (0.10)	100%	0.31 (0.10)	100%	0.57 (0.02)	0.55 (0.01)	0.56 (0.01)	0.56 (0.02)	0.56 (0.01)
	CF+SC	0.65 (0.05)	0.66 (0.06)	0.66 (0.05)	0.66 (0.05)	0.66 (0.05)	0.32 (0.10)	100%	0.30 (0.10)	100%	0.31 (0.10)	100%	0.31 (0.10)	100%	0.32 (0.10)	100%	0.58 (0.01)	0.56 (0.02)	0.56 (0.02)	0.57 (0.01)	0.57 (0.01)
	CF+ GMV	0.65 (0.06)	0.66 (0.06)	0.66 (0.05)	0.66 (0.06)	0.66 (0.05)	0.32 (0.10)	100%	0.29 (0.10)	98%	0.31 (0.10)	100%	0.31 (0.10)	100%	0.31 (0.10)	100%	0.58 (0.01)	0.55 (0.02)	0.57 (0.00)	0.57 (0.01)	0.57 (0.01)
	CF+FC+ GMV	0.65 (0.06)	0.67 (0.05)	0.66 (0.05)	0.66 (0.06)	0.66 (0.05)	0.32 (0.10)	100%	0.29 (0.11)	99%	0.31 (0.10)	100%	0.31 (0.10)	100%	0.31 (0.10)	100%	0.57 (0.01)	0.55 (0.01)	0.57 (0.01)	0.57 (0.01)	0.57 (0.01)
	CF+SC+ GMV	0.65 (0.05)	0.66 (0.05)	0.66 (0.05)	0.65 (0.06)	0.66 (0.05)	0.33 (0.10)	100%	0.30 (0.10)	100%	0.31 (0.10)	100%	0.32 (0.10)	100%	0.32 (0.09)	100%	0.58 (0.01)	0.56 (0.02)	0.57 (0.01)	0.57 (0.01)	0.57 (0.01)
	CF+FC+ SC	0.65 (0.05)	0.66 (0.05)	0.66 (0.05)	0.66 (0.05)	0.66 (0.05)	0.32 (0.10)	100%	0.30 (0.10)	100%	0.30 (0.10)	100%	0.31 (0.10)	100%	0.31 (0.09)	100%	0.57 (0.01)	0.56 (0.01)	0.56 (0.02)	0.56 (0.01)	0.57 (0.01)
	CF+FC+SC +GMV	0.65 (0.05)	0.66 (0.05)	0.66 (0.05)	0.66 (0.05)	0.66 (0.05)	0.32 (0.10)	100%	0.30 (0.10)	100%	0.31 (0.10)	100%	0.31 (0.10)	100%	0.31 (0.09)	100%	0.58 (0.01)	0.56 (0.01)	0.57 (0.02)	0.57 (0.01)	0.57 (0.01)

Supplementary Table S25. Regression performance for MEMORY & EXECUTIVE component in the concatenation approach for FSet A.

**FSet A**	**Comp 1**	Unimodal			Multimodal				Unimodal						Multimodal							
	**Concat**	FC	SC	GMV	FC+ GMV	SC+ GMV	FC+ SC	FC+SC +GMV	FC		SC		GMV		FC+ GMV		SC+ GMV		FC+ SC		FC+SC+GMV	
Cond.	Algo.	MAE	MAE	MAE	MAE	MAE	MAE	MAE	R^2	folds > ref.	R^2	folds > ref.	R^2	folds > ref.	R^2	folds > ref.	R^2	folds > ref.	R^2	folds > ref.	R^2	folds > ref.
no-deconf.	EN	0.77 (0.03)	0.76 (0.03)	0.77 (0.03)	0.75 (0.04)	0.75 (0.04)	0.75 (0.04)	0.75 (0.04)	0.04 (0.05)	81%	0.07 (0.05)	89%	0.05 (0.05)	83%	0.07 (0.06)	89%	0.08 (0.06)	90%	0.08 (0.06)	90%	0.10 (0.06)	95%
	RF	0.76 (0.03)	0.77 (0.03)	0.77 (0.04)	0.75 (0.03)	0.76 (0.03)	0.76 (0.03)	0.75 (0.03)	0.05 (0.06)	79%	0.06 (0.05)	87%	0.04 (0.06)	76%	0.07 (0.06)	88%	0.08 (0.05)	92%	0.07 (0.05)	89%	0.09 (0.05)	98%
	SVR	0.76 (0.03)	0.77 (0.03)	0.77 (0.03)	0.76 (0.04)	0.76 (0.04)	0.76 (0.03)	0.75 (0.04)	0.04 (0.05)	83%	0.05 (0.05)	85%	0.05 (0.05)	84%	0.07 (0.06)	90%	0.07 (0.06)	86%	0.07 (0.05)	90%	0.10 (0.06)	94%
	Ridge	0.76 (0.03)	0.77 (0.03)	0.77 (0.04)	0.75 (0.03)	0.76 (0.04)	0.75 (0.03)	0.74 (0.03)	0.04 (0.04)	85%	0.05 (0.04)	85%	0.05 (0.06)	81%	0.08 (0.05)	92%	0.07 (0.06)	88%	0.08 (0.05)	95%	0.11 (0.05)	96%
	Lasso	0.77 (0.03)	0.77 (0.03)	0.77 (0.03)	0.76 (0.03)	0.76 (0.03)	0.76 (0.03)	0.75 (0.03)	0.03 (0.04)	76%	0.05 (0.04)	90%	0.03 (0.04)	79%	0.04 (0.05)	83%	0.07 (0.04)	95%	0.06 (0.04)	90%	0.07 (0.05)	95%
deconf.	EN	0.79 (0.04)	0.80 (0.03)	0.79 (0.04)	0.79 (0.04)	0.79 (0.04)	0.79 (0.03)	0.79 (0.04)	0.00 (0.03)	59%	0.00 (0.02)	25%	0.01 (0.03)	65%	0.00 (0.04)	62%	0.00 (0.03)	49%	0.00 (0.02)	53%	0.00 (0.04)	63%
	RF	0.79 (0.04)	0.80 (0.04)	0.79 (0.04)	0.79 (0.04)	0.80 (0.04)	0.80 (0.04)	0.80 (0.04)	0.00 (0.04)	53%	-0.02 (0.03)	27%	-0.01 (0.04)	42%	0.00 (0.04)	52%	-0.02 (0.04)	34%	0.00 (0.04)	50%	0.00 (0.04)	50%
	SVR	0.79 (0.04)	0.79 (0.03)	0.79 (0.04)	0.79 (0.04)	0.79 (0.03)	0.79 (0.04)	0.79 (0.04)	0.00 (0.04)	51%	0.00 (0.02)	54%	0.01 (0.03)	62%	0.01 (0.05)	64%	0.01 (0.03)	66%	-0.01 (0.04)	46%	0.01 (0.05)	57%
	Ridge	0.79 (0.03)	0.79 (0.03)	0.79 (0.04)	0.79 (0.04)	0.79 (0.03)	0.79 (0.03)	0.79 (0.04)	0.00 (0.02)	67%	0.00 (0.02)	47%	0.00 (0.04)	64%	0.01 (0.04)	69%	0.00 (0.03)	55%	0.00 (0.02)	64%	0.01 (0.03)	68%
	Lasso	0.79 (0.03)	0.79 (0.03)	0.79 (0.03)	0.79 (0.03)	0.79 (0.03)	0.79 (0.04)	0.80 (0.03)	0.00 (0.02)	41%	0.00 (0.01)	12%	0.00 (0.01)	6%	0.00 (0.02)	17%	0.00 (0.01)	6%	0.00 (0.02)	27%	-0.01 (0.02)	10%

Supplementary Table S26. Regression performance (correlation) for MEMORY & EXECUTIVE component in the concatenation approach for FSet A.

**FSet A**	**Comp 1**	Unimodal			Multimodal			
	**Concat**	FC	SC	GMV	FC+ GMV	SC+ GMV	FC+ SC	FC+SC+GMV
Cond.	Algo.	r	r	r	r	r	r	r
no-deconf.	EN	0.20 (0.01)	0.26 (0.03)	0.22 (0.02)	0.27 (0.02)	0.29 (0.02)	0.28 (0.02)	0.32 (0.02)
	RF	0.22 (0.02)	0.24 (0.03)	0.21 (0.01)	0.27 (0.02)	0.29 (0.02)	0.27 (0.02)	0.31 (0.01)
	SVR	0.21 (0.01)	0.22 (0.01)	0.23 (0.01)	0.27 (0.02)	0.28 (0.02)	0.27 (0.01)	0.31 (0.01)
	Ridge	0.21 (0.01)	0.22 (0.01)	0.23 (0.02)	0.28 (0.02)	0.28 (0.03)	0.29 (0.01)	0.33 (0.01)
	Lasso	0.17 (0.02)	0.24 (0.02)	0.17 (0.02)	0.20 (0.01)	0.27 (0.02)	0.25 (0.02)	0.27 (0.01)
deconf.	EN	0.06 (0.02)	0.00 (0.06)	0.09 (0.02)	0.07 (0.04)	0.08 (0.03)	0.05 (0.04)	0.07 (0.02)
	RF	0.09 (0.02)	-0.02 (0.02)	0.05 (0.03)	0.08 (0.02)	0.01 (0.03)	0.05 (0.04)	0.06 (0.02)
	SVR	0.06 (0.02)	0.05 (0.02)	0.09 (0.02)	0.11 (0.01)	0.10 (0.04)	0.07 (0.02)	0.11 (0.01)
	Ridge	0.06 (0.02)	0.01 (0.02)	0.08 (0.03)	0.08 (0.02)	0.08 (0.03)	0.06 (0.02)	0.10 (0.03)
	Lasso	0.04 (0.05)	-0.03 (0.03)	-0.04 (0.07)	0.00 (0.03)	-0.03 (0.04)	0.02 (0.05)	-0.03 (0.04)

Supplementary Table S27. Regression performance for MEMORY & EXECUTIVE component in the stacking approach for FSet A.

**FSet A**	**Comp 1**	Unimodal			Multimodal				Unimodal						Multimodal							
	**Stack**	FC	SC	GMV	FC+ GMV	SC+ GMV	FC+ SC	FC+SC +GMV	FC		SC		GMV		FC+ GMV		SC+ GMV		FC+ SC		FC+SC+GMV	
Cond.	Algo.	MAE	MAE	MAE	MAE	MAE	MAE	MAE	R^2	folds > ref.	R^2	folds > ref.	R^2	folds > ref.	R^2	folds > ref.	R^2	folds > ref.	R^2	folds > ref.	R^2	folds > ref.
no-deconf.	EN	0.77 (0.03)	0.77 (0.03)	0.77 (0.03)	0.76 (0.04)	0.76 (0.04)	0.76 (0.04)	0.75 (0.04)	0.04 (0.04)	82%	0.06 (0.05)	91%	0.05 (0.05)	85%	0.06 (0.07)	83%	0.06 (0.07)	81%	0.07 (0.07)	81%	0.09 (0.07)	88%
	RF	0.76 (0.03)	0.77 (0.03)	0.77 (0.03)	0.76 (0.03)	0.77 (0.04)	0.76 (0.04)	0.76 (0.04)	0.05 (0.05)	83%	0.06 (0.05)	91%	0.04 (0.05)	85%	0.06 (0.06)	86%	0.05 (0.07)	74%	0.05 (0.08)	80%	0.07 (0.08)	84%
	SVR	0.76 (0.03)	0.77 (0.03)	0.77 (0.04)	0.76 (0.04)	0.76 (0.04)	0.76 (0.04)	0.75 (0.04)	0.05 (0.05)	85%	0.05 (0.05)	86%	0.05 (0.05)	85%	0.06 (0.08)	76%	0.06 (0.08)	84%	0.07 (0.08)	83%	0.09 (0.07)	88%
	Ridge	0.76 (0.03)	0.77 (0.03)	0.77 (0.04)	0.76 (0.04)	0.77 (0.04)	0.75 (0.04)	0.75 (0.04)	0.04 (0.04)	85%	0.05 (0.05)	85%	0.05 (0.06)	80%	0.07 (0.07)	84%	0.06 (0.08)	79%	0.08 (0.07)	84%	0.09 (0.07)	92%
	Lasso	0.77 (0.03)	0.77 (0.03)	0.77 (0.03)	0.77 (0.04)	0.76 (0.04)	0.76 (0.04)	0.76 (0.04)	0.03 (0.04)	76%	0.05 (0.04)	90%	0.03 (0.04)	79%	0.03 (0.06)	67%	0.05 (0.06)	82%	0.05 (0.06)	83%	0.06 (0.06)	88%
deconf.	EN	0.79 (0.04)	0.80 (0.03)	0.79 (0.04)	0.80 (0.04)	0.80 (0.04)	0.80 (0.04)	0.80 (0.04)	0.00 (0.03)	60%	-0.01 (0.02)	19%	0.01 (0.03)	66%	-0.02 (0.05)	40%	-0.02 (0.05)	36%	-0.02 (0.04)	35%	-0.02 (0.04)	33%
	RF	0.79 (0.04)	0.80 (0.04)	0.79 (0.04)	0.80 (0.04)	0.80 (0.04)	0.80 (0.04)	0.80 (0.04)	0.00 (0.04)	52%	-0.02 (0.03)	19%	-0.01 (0.04)	66%	-0.02 (0.05)	35%	-0.03 (0.05)	29%	-0.02 (0.05)	31%	-0.03 (0.06)	36%
	SVR	0.79 (0.04)	0.79 (0.03)	0.79 (0.03)	0.80 (0.04)	0.80 (0.04)	0.80 (0.04)	0.80 (0.04)	0.00 (0.04)	51%	0.00 (0.02)	55%	0.01 (0.03)	64%	-0.02 (0.06)	35%	-0.02 (0.04)	31%	-0.02 (0.04)	32%	-0.02 (0.05)	34%
	Ridge	0.79 (0.04)	0.79 (0.04)	0.79 (0.04)	0.80 (0.04)	0.80 (0.04)	0.80 (0.04)	0.80 (0.04)	0.00 (0.02)	64%	0.00 (0.02)	49%	0.00 (0.04)	65%	-0.02 (0.05)	40%	-0.02 (0.05)	38%	-0.01 (0.06)	44%	-0.01 (0.06)	40%
	Lasso	0.79 (0.03)	0.79 (0.03)	0.79 (0.03)	0.80 (0.04)	0.80 (0.03)	0.80 (0.04)	0.80 (0.04)	0.00 (0.02)	42%	0.00 (0.01)	6%	0.00 (0.01)	6%	-0.01 (0.04)	33%	-0.01 (0.03)	7%	-0.01 (0.04)	32%	-0.02 (0.04)	31%

Supplementary Table S28. Regression performance (correlation) for MEMORY & EXECUTIVE component in the stacking approach for FSet A.

**FSet A**	**Comp 1**	Unimodal			Multimodal			
	**Stack**	FC	SC	GMV	FC+ GMV	SC+ GMV	FC+ SC	FC+SC+GMV
Cond.	Algo.	r	r	r	r	r	r	r
no-deconf.	EN	0.21 (0.01)	0.24 (0.02)	0.28 (0.01)	0.30 (0.02)	0.28 (0.02)	0.27 (0.03)	0.32 (0.02)
	RF	0.24 (0.01)	0.23 (0.02)	0.25 (0.02)	0.28 (0.02)	0.24 (0.02)	0.25 (0.04)	0.29 (0.04)
	SVR	0.23 (0.01)	0.23 (0.02)	0.29 (0.01)	0.30 (0.02)	0.29 (0.03)	0.28 (0.03)	0.33 (0.02)
	Ridge	0.23 (0.01)	0.22 (0.02)	0.28 (0.01)	0.30 (0.03)	0.27 (0.03)	0.29 (0.03)	0.32 (0.02)
	Lasso	0.18 (0.02)	0.23 (0.01)	0.23 (0.01)	0.22 (0.03)	0.24 (0.02)	0.24 (0.05)	0.28 (0.04)
deconf.	EN	0.12 (0.04)	0.00 (0.02)	0.15 (0.03)	0.11 (0.04)	0.06 (0.04)	0.07 (0.04)	0.10 (0.03)
	RF	0.16 (0.02)	0.01 (0.02)	0.09 (0.04)	0.08 (0.05)	0.04 (0.06)	0.07 (0.04)	0.08 (0.04)
	SVR	0.14 (0.01)	0.07 (0.02)	0.15 (0.03)	0.07 (0.04)	0.06 (0.03)	0.04 (0.04)	0.07 (0.06)
	Ridge	0.15 (0.03)	0.03 (0.02)	0.12 (0.03)	0.09 (0.06)	0.07 (0.05)	0.07 (0.04)	0.09 (0.06)
	Lasso	0.10 (0.01)	-0.02 (0.04)	0.02 (0.01)	0.07 (0.04)	-0.01 (0.08)	0.07 (0.07)	0.06 (0.05)

Supplementary Table S29. Regression performance for MEMORY & EXECUTIVE component in the concatenation approach for FSet B.

**FSet B**	**Comp 1**	Unimodal			Multimodal				Unimodal						Multimodal							
	**Concat**	FC	SC	GMV	FC+ GMV	SC+ GMV	FC+ SC	FC+SC +GMV	FC		SC		GMV		FC+ GMV		SC+ GMV		FC+ SC		FC+SC+GMV	
Cond.	Algo.	MAE	MAE	MAE	MAE	MAE	MAE	MAE	R^2	Folds > ref	R^2	folds > ref	R^2	folds > ref	R^2	folds > ref	R^2	folds > ref	R^2	folds > ref	R^2	folds > ref
no-deconf.	EN	0.78 (0.03)	0.76 (0.03)	0.77 (0.04)	0.76 (0.03)	0.76 (0.03)	0.76 (0.03)	0.75 (0.04)	0.02 (0.03)	71%	0.07 (0.05)	93%	0.05 (0.05)	85%	0.07 (0.05)	89%	0.07 (0.06)	93%	0.06 (0.05)	86%	0.08 (0.06)	93%
	RF	0.78 (0.03)	0.77 (0.03)	0.77 (0.04)	0.76 (0.03)	0.75 (0.03)	0.77 (0.03)	0.76 (0.03)	0.01 (0.04)	61%	0.06 (0.06)	84%	0.04 (0.06)	76%	0.05 (0.05)	82%	0.08 (0.05)	92%	0.06 (0.05)	87%	0.08 (0.05)	95%
	SVR	0.77 (0.03)	0.78 (0.03)	0.77 (0.03)	0.76 (0.03)	0.76 (0.04)	0.77 (0.03)	0.76 (0.04)	0.03 (0.05)	76%	0.04 (0.04)	87%	0.05 (0.05)	85%	0.06 (0.06)	85%	0.07 (0.06)	87%	0.04 (0.05)	88%	0.07 (0.07)	87%
	Ridge	0.78 (0.03)	0.77 (0.04)	0.77 (0.04)	0.76 (0.03)	0.76 (0.04)	0.77 (0.03)	0.76 (0.04)	0.00 (0.04)	64%	0.05 (0.06)	85%	0.05 (0.06)	81%	0.05 (0.06)	83%	0.06 (0.07)	86%	0.04 (0.05)	81%	0.07 (0.07)	88%
	Lasso	0.78 (0.03)	0.76 (0.03)	0.77 (0.03)	0.77 (0.03)	0.76 (0.03)	0.76 (0.03)	0.76 (0.03)	0.01 (0.03)	72%	0.06 (0.04)	93%	0.03 (0.04)	79%	0.03 (0.04)	76%	0.07 (0.04)	96%	0.06 (0.04)	95%	0.07 (0.04)	95%
deconf.	EN	0.80 (0.03)	0.79 (0.03)	0.79 (0.03)	0.79 (0.03)	0.79 (0.04)	0.79 (0.03)	0.79 (0.04)	-0.01 (0.01)	35%	0.00 (0.02)	44%	0.01 (0.03)	65%	0.01 (0.03)	61%	0.01 (0.03)	64%	0.00 (0.02)	48%	0.01 (0.03)	70%
	RF	0.80 (0.04)	0.80 (0.04)	0.79 (0.04)	0.80 (0.04)	0.79 (0.03)	0.80 (0.04)	0.79 (0.04)	-0.01 (0.03)	38%	0.00 (0.04)	56%	-0.01 (0.04)	42%	-0.01 (0.03)	43%	0.00 (0.03)	54%	0.00 (0.03)	53%	0.00 (0.04)	60%
	SVR	0.79 (0.03)	0.79 (0.03)	0.79 (0.04)	0.79 (0.04)	0.79 (0.04)	0.79 (0.04)	0.79 (0.04)	0.00 (0.02)	52%	0.00 (0.02)	59%	0.01 (0.03)	64%	0.01 (0.04)	60%	0.01 (0.03)	68%	0.00 (0.03)	52%	0.01 (0.04)	70%
	Ridge	0.79 (0.03)	0.79 (0.03)	0.79 (0.04)	0.79 (0.04)	0.79 (0.04)	0.79 (0.03)	0.79 (0.04)	0.00 (0.01)	59%	0.00 (0.02)	62%	0.00 (0.04)	65%	0.00 (0.03)	64%	0.00 (0.04)	61%	0.00 (0.02)	68%	0.00 (0.04)	72%
	Lasso	0.79 (0.03)	0.79 (0.03)	0.79 (0.03)	0.79 (0.03)	0.79 (0.03)	0.79 (0.03)	0.79 (0.03)	0.00 (0.01)	13%	0.00 (0.01)	6%	0.00 (0.01)	5%	0.00 (0.01)	7%	0.00 (0.00)	5%	0.00 (0.01)	6%	0.00 (0.01)	5%

Supplementary Table S30. Regression performance (correlation) for MEMORY & EXECUTIVE component in the concatenation approach for FSet B.

**FSet B**	**Comp 1**	Unimodal			Multimodal			
	**Concat**	FC	SC	GMV	FC+ GMV	SC+ GMV	FC+ SC	FC+SC+GMV
Cond.	Algo.	r	r	r	r	r	r	r
no-deconf.	EN	0.13 (0.02)	0.27 (0.02)	0.22 (0.01)	0.26 (0.01)	0.27 (0.01)	0.24 (0.02)	0.28 (0.02)
	RF	0.12 (0.03)	0.24 (0.03)	0.21 (0.02)	0.23 (0.02)	0.29 (0.01)	0.25 (0.03)	0.28 (0.02)
	SVR	0.16 (0.01)	0.20 (0.02)	0.23 (0.01)	0.25 (0.01)	0.27 (0.03)	0.21 (0.01)	0.27 (0.02)
	Ridge	0.10 (0.02)	0.22 (0.02)	0.23 (0.02)	0.24 (0.02)	0.25 (0.02)	0.20 (0.02)	0.26 (0.02)
	Lasso	0.12 (0.01)	0.27 (0.02)	0.17 (0.02)	0.17 (0.01)	0.28 (0.02)	0.26 (0.01)	0.27 (0.01)
deconf.	EN	-0.02 (0.04)	0.01 (0.05)	0.09 (0.01)	0.10 (0.04)	0.08 (0.03)	0.03 (0.03)	0.09 (0.04)
	RF	0.03 (0.02)	0.07 (0.03)	0.06 (0.02)	0.05 (0.03)	0.08 (0.05)	0.07 (0.03)	0.06 (0.04)
	SVR	0.02 (0.04)	0.05 (0.03)	0.09 (0.02)	0.11 (0.03)	0.10 (0.04)	0.05 (0.02)	0.12 (0.02)
	Ridge	0.01 (0.02)	0.02 (0.05)	0.09 (0.02)	0.08 (0.03)	0.07 (0.05)	0.04 (0.02)	0.08 (0.04)
	Lasso	-0.02 (0.06)	-0.02 (0.05)	-0.05 (0.04)	-0.03 (0.05)	-0.01 (0.00)	-0.01 (0.02)	-0.01 (0.03)

Supplementary Table S31. Regression performance for MEMORY & EXECUTIVE component in the stacking approach for FSet B.

**FSet B**	**Comp 1**	Unimodal			Multimodal				Unimodal						Multimodal							
	**Stack**	FC	SC	GMV	FC+ GMV	SC+ GMV	FC+ SC	FC+SC +GMV	FC		SC		GMV		FC+ GMV		SC+ GMV		FC+ SC		FC+SC+GMV	
Cond.	Algo.	MAE	MAE	MAE	MAE	MAE	MAE	MAE	R^2	folds > ref.	R^2	folds > ref.	R^2	folds > ref.	R^2	folds > ref.	R^2	folds > ref.	R^2	folds > ref.	R^2	folds > ref.
no-deconf.	EN	0.78 (0.03)	0.76 (0.03)	0.77 (0.03)	0.77 (0.04)	0.76 (0.04)	0.77 (0.04)	0.76 (0.03)	0.02 (0.03)	72%	0.07 (0.05)	91%	0.05 (0.05)	84%	0.04 (0.07)	73%	0.06 (0.07)	85%	0.04 (0.07)	74%	0.07 (0.06)	84%
	RF	0.78 (0.03)	0.77 (0.03)	0.77 (0.03)	0.78 (0.03)	0.77 (0.04)	0.77 (0.04)	0.76 (0.04)	0.01 (0.04)	61%	0.05 (0.05)	91%	0.04 (0.06)	84%	0.03 (0.07)	71%	0.05 (0.07)	82%	0.04 (0.07)	76%	0.06 (0.07)	81%
	SVR	0.77 (0.03)	0.77 (0.03)	0.77 (0.03)	0.77 (0.04)	0.76 (0.04)	0.77 (0.04)	0.76 (0.04)	0.03 (0.05)	77%	0.04 (0.04)	86%	0.05 (0.05)	86%	0.03 (0.07)	67%	0.06 (0.07)	80%	0.04 (0.07)	78%	0.06 (0.07)	81%
	Ridge	0.78 (0.03)	0.77 (0.04)	0.77 (0.04)	0.77 (0.04)	0.76 (0.04)	0.78 (0.04)	0.76 (0.04)	0.00 (0.05)	64%	0.04 (0.05)	85%	0.05 (0.06)	81%	0.04 (0.07)	77%	0.06 (0.07)	85%	0.02 (0.06)	60%	0.05 (0.07)	82%
	Lasso	0.78 (0.03)	0.76 (0.03)	0.77 (0.03)	0.78 (0.04)	0.76 (0.03)	0.77 (0.04)	0.76 (0.04)	0.01 (0.03)	71%	0.06 (0.04)	93%	0.03 (0.04)	79%	0.00 (0.05)	55%	0.05 (0.06)	83%	0.04 (0.06)	77%	0.05 (0.06)	79%
deconf.	EN	0.80 (0.03)	0.79 (0.03)	0.79 (0.04)	0.80 (0.04)	0.80 (0.04)	0.80 (0.04)	0.80 (0.04)	-0.01 (0.01)	34%	0.00 (0.02)	43%	0.01 (0.03)	68%	-0.02 (0.04)	38%	-0.01 (0.04)	46%	-0.02 (0.04)	28%	-0.02 (0.04)	34%
	RF	0.80 (0.04)	0.79 (0.04)	0.79 (0.04)	0.80 (0.04)	0.80 (0.04)	0.80 (0.04)	0.80 (0.04)	-0.01 (0.03)	36%	0.00 (0.04)	43%	-0.01 (0.04)	68%	-0.02 (0.05)	24%	-0.02 (0.05)	37%	-0.02 (0.05)	34%	-0.02 (0.05)	31%
	SVR	0.79 (0.03)	0.79 (0.03)	0.79 (0.03)	0.80 (0.04)	0.80 (0.04)	0.81 (0.04)	0.80 (0.04)	-0.01 (0.02)	52%	0.00 (0.02)	59%	0.01 (0.03)	64%	-0.02 (0.06)	38%	-0.02 (0.04)	31%	-0.03 (0.04)	27%	-0.02 (0.04)	31%
	Ridge	0.79 (0.03)	0.79 (0.03)	0.79 (0.04)	0.80 (0.04)	0.80 (0.04)	0.80 (0.04)	0.80 (0.04)	0.00 (0.01)	58%	0.00 (0.01)	63%	0.00 (0.04)	66%	-0.02 (0.06)	35%	-0.03 (0.05)	34%	-0.03 (0.04)	20%	-0.03 (0.05)	31%
	Lasso	0.79 (0.03)	0.79 (0.03)	0.79 (0.03)	0.80 (0.03)	0.80 (0.03)	0.80 (0.03)	0.80 (0.03)	0.00 (0.01)	8%	0.00 (0.01)	5%	0.00 (0.01)	8%	-0.01 (0.02)	5%	-0.01 (0.02)	5%	-0.01 (0.03)	3%	-0.01 (0.03)	5%

Supplementary Table S32. Regression performance (correlation) for MEMORY & EXECUTIVE component in the stacking approach for FSet B.

**FSet B**	**Comp 1**	Unimodal			Multimodal			
	**Stack**	FC	SC	GMV	FC+ GMV	SC+ GMV	FC+ SC	FC+SC+GMV
Cond.	Algo.	r	r	r	r	r	r	r
no-deconf.	EN	0.13 (0.01)	0.26 (0.02)	0.28 (0.02)	0.25 (0.04)	0.26 (0.02)	0.21 (0.03)	0.28 (0.01)
	RF	0.13 (0.02)	0.22 (0.02)	0.26 (0.03)	0.23 (0.03)	0.25 (0.04)	0.22 (0.03)	0.27 (0.04)
	SVR	0.17 (0.01)	0.19 (0.01)	0.29 (0.01)	0.25 (0.02)	0.27 (0.02)	0.21 (0.03)	0.29 (0.02)
	Ridge	0.11 (0.02)	0.21 (0.01)	0.28 (0.02)	0.26 (0.06)	0.25 (0.04)	0.18 (0.03)	0.27 (0.04)
	Lasso	0.12 (0.03)	0.25 (0.01)	0.23 (0.01)	0.17 (0.05)	0.24 (0.03)	0.21 (0.03)	0.26 (0.01)
deconf.	EN	0.02 (0.07)	0.02 (0.03)	0.14 (0.01)	0.06 (0.01)	0.06 (0.02)	-0.01 (0.06)	0.04 (0.07)
	RF	0.06 (0.01)	0.09 (0.03)	0.11 (0.04)	0.04 (0.05)	0.06 (0.03)	0.05 (0.04)	0.06 (0.05)
	SVR	0.05 (0.04)	0.05 (0.01)	0.15 (0.02)	0.08 (0.04)	0.05 (0.06)	0.00 (0.06)	0.06 (0.06)
	Ridge	0.06 (0.02)	0.04 (0.02)	0.13 (0.03)	0.04 (0.03)	0.04 (0.04)	-0.02 (0.02)	0.02 (0.03)
	Lasso	-0.02 (0.05)	-0.03 (0.03)	0.01 (0.05)	0.00 (0.07)	0.00 (0.01)	0.00 (0.04)	0.00 (0.06)

Supplementary Table S33. Regression performance for VERBAL MEMORY & LANGUAGE component in the concatenation approach in FSet A.

**FSet A**	**Comp 2**	Unimodal			Multimodal				Unimodal						Multimodal							
	**Concat**	FC	SC	GMV	FC+ GMV	SC+ GMV	FC+ SC	FC+SC +GMV	FC		SC		GMV		FC+ GMV		SC+ GMV		FC+ SC		FC+SC+GMV	
Cond.	Algo.	MAE	MAE	MAE	MAE	MAE	MAE	MAE	R^2	folds > ref.	R^2	folds > ref.	R^2	folds > ref	R^2	folds > ref.	R^2	folds > ref.	R^2	folds > ref.	R^2	folds > ref
no-deconf.	EN	0.81 (0.03)	0.79 (0.03)	0.80 (0.03)	0.81 (0.03)	0.79 (0.03)	0.80 (0.03)	0.80 (0.03)	0.00 (0.02)	50%	0.04 (0.05)	81%	0.02 (0.04)	72%	0.01 (0.03)	59%	0.04 (0.05)	78%	0.03 (0.04)	75%	0.03 (0.04)	78%
	RF	0.82 (0.03)	0.79 (0.03)	0.80 (0.03)	0.81 (0.03)	0.79 (0.03)	0.80 (0.03)	0.80 (0.03)	-0.02 (0.03)	33%	0.04 (0.05)	75%	0.02 (0.04)	69%	0.00 (0.04)	54%	0.04 (0.05)	78%	0.02 (0.05)	68%	0.03 (0.05)	72%
	SVR	0.81 (0.03)	0.79 (0.03)	0.81 (0.03)	0.81 (0.03)	0.79 (0.03)	0.80 (0.04)	0.80 (0.04)	0.00 (0.03)	57%	0.04 (0.06)	81%	0.01 (0.03)	75%	0.01 (0.05)	66%	0.05 (0.05)	85%	0.03 (0.06)	74%	0.03 (0.06)	72%
	Ridge	0.81 (0.03)	0.79 (0.03)	0.81 (0.03)	0.80 (0.03)	0.79 (0.03)	0.79 (0.03)	0.79 (0.03)	0.00 (0.02)	60%	0.05 (0.04)	85%	0.02 (0.04)	74%	0.01 (0.03)	66%	0.05 (0.05)	86%	0.04 (0.05)	79%	0.04 (0.05)	84%
	Lasso	0.81 (0.03)	0.79 (0.03)	0.80 (0.03)	0.81 (0.03)	0.79 (0.03)	0.80 (0.03)	0.79 (0.03)	0.00 (0.01)	14%	0.04 (0.04)	82%	0.01 (0.02)	60%	0.00 (0.02)	47%	0.04 (0.04)	79%	0.03 (0.04)	79%	0.03 (0.04)	79%
deconf.	EN	0.81 (0.03)	0.81 (0.03)	0.82 (0.03)	0.81 (0.03)	0.81 (0.03)	0.81 (0.03)	0.82 (0.03)	0.00 (0.01)	22%	0.00 (0.02)	61%	-0.01 (0.02)	22%	0.00 (0.01)	10%	0.00 (0.02)	56%	0.00 (0.02)	38%	0.00 (0.01)	37%
	RF	0.82 (0.03)	0.82 (0.03)	0.81 (0.03)	0.82 (0.03)	0.82 (0.03)	0.82 (0.03)	0.82 (0.03)	-0.02 (0.03)	17%	0.00 (0.03)	48%	0.00 (0.03)	52%	-0.02 (0.03)	31%	-0.01 (0.04)	54%	-0.01 (0.03)	48%	-0.01 (0.03)	42%
	SVR	0.82 (0.03)	0.81 (0.03)	0.82 (0.03)	0.82 (0.03)	0.81 (0.03)	0.82 (0.03)	0.82 (0.03)	-0.02 (0.02)	20%	0.00 (0.03)	59%	0.00 (0.02)	54%	-0.01 (0.03)	32%	0.00 (0.04)	55%	-0.01 (0.04)	43%	-0.01 (0.04)	43%
	Ridge	0.81 (0.03)	0.81 (0.03)	0.81 (0.03)	0.81 (0.03)	0.81 (0.03)	0.81 (0.03)	0.81 (0.03)	0.00 (0.01)	33%	0.01 (0.02)	70%	0.00 (0.02)	58%	0.00 (0.01)	47%	0.00 (0.02)	68%	0.00 (0.02)	69%	0.00 (0.02)	68%
	Lasso	0.81 (0.03)	0.81 (0.03)	0.81 (0.03)	0.81 (0.03)	0.81 (0.03)	0.81 (0.03)	0.81 (0.03)	0.00 (0.00)	4%	0.00 (0.01)	20%	0.00 (0.00)	8%	0.00 (0.00)	5%	0.00 (0.01)	11%	0.00 (0.01)	5%	0.00 (0.01)	6%

Supplementary Table S34. Regression performance (correlation) for VERBAL MEMORY & LANGUAGE component in the concatenation approach in FSet A.

**FSet A**	**Comp 2**	Unimodal			Multimodal			
	**Concat**	FC	SC	GMV	FC+ GMV	SC+ GMV	FC+ SC	FC+SC+GMV
Cond.	Algo.	r	r	r	r	r	r	r
no-deconf.	EN	0.03 (0.03)	0.21 (0.02)	0.13 (0.05)	0.09 (0.03)	0.21 (0.03)	0.17 (0.01)	0.18 (0.02)
	RF	0.00 (0.02)	0.20 (0.03)	0.13 (0.02)	0.06 (0.05)	0.20 (0.02)	0.16 (0.02)	0.17 (0.02)
	SVR	0.08 (0.03)	0.20 (0.04)	0.12 (0.02)	0.11 (0.03)	0.22 (0.02)	0.17 (0.02)	0.19 (0.02)
	Ridge	0.04 (0.03)	0.22 (0.01)	0.13 (0.01)	0.11 (0.04)	0.23 (0.03)	0.19 (0.01)	0.21 (0.02)
	Lasso	-0.01 (0.03)	0.20 (0.02)	0.09 (0.04)	0.06 (0.04)	0.19 (0.03)	0.18 (0.01)	0.18 (0.02)
deconf.	EN	-0.01 (0.07)	0.07 (0.02)	0.00 (0.03)	-0.03 (0.05)	0.06 (0.06)	0.03 (0.05)	0.01 (0.03)
	RF	-0.06 (0.04)	0.06 (0.02)	0.06 (0.03)	0.00 (0.03)	0.05 (0.03)	0.04 (0.02)	0.04 (0.03)
	SVR	-0.07 (0.02)	0.09 (0.02)	0.05 (0.04)	-0.02 (0.02)	0.08 (0.03)	0.05 (0.02)	0.04 (0.03)
	Ridge	-0.07 (0.03)	0.08 (0.02)	0.02 (0.05)	-0.03 (0.04)	0.07 (0.02)	0.05 (0.04)	0.06 (0.04)
	Lasso	-0.02 (0.03)	0.02 (0.01)	-0.02 (0.01)	0.00 (0.01)	0.01 (0.03)	-0.01 (0.04)	-0.02 (0.03)

Supplementary Table S35. Regression performance for VERBAL MEMORY & LANGUAGE component in the stacking approach in FSet A.

**FSet A**	**Comp 2**	Unimodal			Multimodal				Unimodal						Multimodal							
	**Stack**	FC	SC	GMV	FC+ GMV	SC+ GMV	FC+ SC	FC+SC +GMV	FC		SC		GMV		FC+ GMV		SC+ GMV		FC+ SC		FC+SC+GMV	
Cond.	Algo.	MAE	MAE	MAE	MAE	MAE	MAE	MAE	R^2	folds > ref.	R^2	folds > ref.	R^2	folds > ref.	R^2	folds > ref.	R^2	folds > ref.	R^2	folds > ref.	R^2	folds > ref.
no-deconf.	EN	0.81 (0.03)	0.79 (0.03)	0.81 (0.03)	0.81 (0.03)	0.81 (0.03)	0.80 (0.03)	0.80 (0.03)	0.00 (0.02)	46%	0.04 (0.05)	83%	0.01 (0.03)	74%	-0.01 (0.05)	49%	0.01 (0.06)	58%	0.02 (0.06)	61%	0.02 (0.05)	63%
	RF	0.81 (0.03)	0.79 (0.03)	0.80 (0.03)	0.82 (0.03)	0.81 (0.04)	0.81 (0.03)	0.82 (0.04)	-0.02 (0.04)	31%	0.04 (0.05)	75%	0.01 (0.04)	67%	-0.03 (0.05)	32%	0.00 (0.07)	53%	-0.01 (0.06)	52%	-0.01 (0.07)	53%
	SVR	0.81 (0.03)	0.79 (0.03)	0.81 (0.03)	0.81 (0.03)	0.81 (0.03)	0.80 (0.03)	0.81 (0.03)	0.00 (0.03)	57%	0.04 (0.05)	85%	0.01 (0.03)	73%	-0.01 (0.05)	47%	0.01 (0.06)	60%	0.01 (0.07)	57%	0.01 (0.06)	66%
	Ridge	0.81 (0.03)	0.79 (0.03)	0.80 (0.03)	0.81 (0.03)	0.80 (0.04)	0.80 (0.04)	0.80 (0.03)	0.00 (0.02)	60%	0.05 (0.04)	87%	0.02 (0.04)	73%	-0.01 (0.06)	47%	0.02 (0.07)	61%	0.02 (0.07)	59%	0.02 (0.06)	63%
	Lasso	0.81 (0.03)	0.79 (0.03)	0.80 (0.03)	0.81 (0.03)	0.80 (0.03)	0.81 (0.03)	0.80 (0.03)	0.00 (0.01)	13%	0.04 (0.04)	82%	0.01 (0.02)	65%	-0.01 (0.04)	39%	0.01 (0.05)	57%	0.01 (0.05)	66%	0.01 (0.05)	65%
deconf.	EN	0.81 (0.03)	0.81 (0.03)	0.81 (0.03)	0.82 (0.03)	0.82 (0.03)	0.82 (0.03)	0.82 (0.03)	0.00 (0.00)	20%	0.00 (0.02)	61%	0.00 (0.02)	26%	-0.02 (0.04)	18%	-0.02 (0.05)	31%	-0.01 (0.04)	37%	-0.02 (0.04)	33%
	RF	0.82 (0.03)	0.82 (0.03)	0.81 (0.03)	0.82 (0.03)	0.82 (0.03)	0.82 (0.03)	0.82 (0.03)	-0.02 (0.03)	18%	0.00 (0.04)	47%	0.00 (0.03)	58%	-0.02 (0.05)	33%	-0.02 (0.05)	33%	-0.02 (0.05)	39%	-0.02 (0.05)	34%
	SVR	0.82 (0.03)	0.81 (0.03)	0.82 (0.03)	0.82 (0.03)	0.82 (0.03)	0.82 (0.03)	0.82 (0.03)	-0.02 (0.02)	20%	0.00 (0.03)	59%	0.00 (0.03)	53%	-0.02 (0.04)	33%	-0.03 (0.05)	27%	-0.02 (0.05)	36%	-0.02 (0.05)	34%
	Ridge	0.81 (0.03)	0.81 (0.03)	0.82 (0.03)	0.82 (0.03)	0.82 (0.04)	0.81 (0.03)	0.82 (0.03)	0.00 (0.01)	33%	0.00 (0.02)	69%	0.00 (0.02)	56%	-0.02 (0.04)	33%	-0.03 (0.05)	30%	-0.01 (0.05)	44%	-0.01 (0.05)	43%
	Lasso	0.81 (0.03)	0.81 (0.03)	0.81 (0.03)	0.81 (0.03)	0.82 (0.03)	0.82 (0.03)	0.82 (0.03)	0.00 (0.00)	6%	0.00 (0.01)	18%	0.00 (0.01)	10%	-0.01 (0.02)	4%	0.00 (0.02)	18%	-0.01 (0.03)	11%	-0.01 (0.03)	14%

Supplementary Table S36. Regression performance (correlation) for VERBAL MEMORY & LANGUAGE component in the stacking approach in FSet A.

**FSet A**	**Comp 2**	Unimodal			Multimodal			
	**Stack**	FC	SC	GMV	FC+ GMV	SC+ GMV	FC+ SC	FC+SC+GMV
Cond.	Algo.	r	r	r	r	r	r	r
no-deconf.	EN	0.02 (0.04)	0.21 (0.01)	0.11 (0.01)	0.07 (0.04)	0.13 (0.03)	0.17 (0.04)	0.16 (0.04)
	RF	0.00 (0.05)	0.19 (0.01)	0.10 (0.01)	0.00 (0.05)	0.11 (0.05)	0.11 (0.04)	0.11 (0.07)
	SVR	0.08 (0.01)	0.21 (0.02)	0.10 (0.02)	0.07 (0.03)	0.15 (0.03)	0.16 (0.04)	0.16 (0.02)
	Ridge	0.04 (0.01)	0.23 (0.01)	0.13 (0.01)	0.08 (0.04)	0.16 (0.02)	0.17 (0.05)	0.18 (0.03)
	Lasso	-0.02 (0.06)	0.20 (0.02)	0.08 (0.03)	0.03 (0.05)	0.13 (0.04)	0.14 (0.02)	0.14 (0.04)
deconf.	EN	-0.02 (0.05)	0.13 (0.02)	0.03 (0.04)	-0.01 (0.06)	0.05 (0.03)	0.09 (0.04)	0.06 (0.04)
	RF	-0.04 (0.03)	0.12 (0.01)	0.07 (0.03)	0.01 (0.05)	0.03 (0.05)	0.05 (0.04)	0.04 (0.06)
	SVR	-0.03 (0.02)	0.15 (0.02)	0.07 (0.04)	0.03 (0.05)	0.02 (0.06)	0.05 (0.05)	0.06 (0.05)
	Ridge	-0.05 (0.02)	0.13 (0.03)	0.04 (0.05)	0.00 (0.01)	0.04 (0.06)	0.10 (0.05)	0.08 (0.05)
	Lasso	0.00 (0.02)	0.04 (0.03)	0.01 (0.05)	0.00 (0.02)	0.03 (0.05)	0.02 (0.05)	0.02 (0.06)

Supplementary Table S37. Regression performance for VERBAL MEMORY & LANGUAGE component in the concatenation approach in FSet B.

**FSet B**	**Comp 2**	Unimodal			Multimodal				Unimodal						Multimodal							
	**Concat**	FC	SC	GMV	FC+ GMV	SC+ GMV	FC+ SC	FC+SC +GMV	FC		SC		GMV		FC+ GMV		SC+ GMV		FC+ SC		FC+SC+GMV	
Cond.	Algo.	MAE	MAE	MAE	MAE	MAE	MAE	MAE	R^2	folds > ref.	R^2	folds > ref.	R^2	Folds > ref.	R^2	folds > ref.	R^2	folds > ref.	R^2	folds > ref.	R^2	folds > ref.
no-deconf.	EN	0.80 (0.03)	0.80 (0.03)	0.81 (0.03)	0.80 (0.03)	0.79 (0.03)	0.80 (0.03)	0.80 (0.03)	0.00 (0.03)	56%	0.03 (0.04)	87%	0.01 (0.04)	68%	0.01 (0.03)	66%	0.05 (0.05)	87%	0.02 (0.03)	81%	0.03 (0.05)	76%
	RF	0.81 (0.03)	0.80 (0.03)	0.81 (0.03)	0.80 (0.03)	0.80 (0.03)	0.80 (0.03)	0.80 (0.03)	-0.01 (0.04)	47%	0.03 (0.04)	79%	0.01 (0.04)	66%	0.02 (0.04)	66%	0.04 (0.05)	83%	0.03 (0.04)	76%	0.03 (0.05)	80%
	SVR	0.80 (0.03)	0.80 (0.03)	0.81 (0.03)	0.80 (0.03)	0.80 (0.03)	0.80 (0.03)	0.80 (0.04)	0.01 (0.03)	60%	0.03 (0.04)	79%	0.02 (0.03)	74%	0.01 (0.04)	63%	0.04 (0.05)	77%	0.02 (0.04)	74%	0.03 (0.06)	75%
	Ridge	0.80 (0.03)	0.80 (0.03)	0.80 (0.03)	0.80 (0.03)	0.80 (0.03)	0.80 (0.03)	0.80 (0.03)	0.01 (0.02)	66%	0.03 (0.04)	78%	0.02 (0.04)	72%	0.01 (0.04)	70%	0.04 (0.05)	83%	0.03 (0.04)	86%	0.04 (0.05)	80%
	Lasso	0.80 (0.03)	0.81 (0.03)	0.80 (0.03)	0.80 (0.03)	0.80 (0.03)	0.81 (0.03)	0.80 (0.03)	0.00 (0.02)	52%	0.01 (0.02)	49%	0.01 (0.02)	60%	0.01 (0.02)	57%	0.02 (0.03)	66%	0.01 (0.03)	58%	0.01 (0.03)	65%
deconf.	EN	0.81 (0.03)	0.81 (0.03)	0.82 (0.03)	0.81 (0.03)	0.82 (0.03)	0.82 (0.03)	0.82 (0.03)	-0.01 (0.01)	4%	0.00 (0.02)	54%	0.00 (0.02)	35%	0.00 (0.02)	5%	0.00 (0.02)	43%	-0.01 (0.03)	31%	-0.01 (0.03)	29%
	RF	0.82 (0.03)	0.81 (0.03)	0.81 (0.03)	0.82 (0.03)	0.81 (0.03)	0.82 (0.03)	0.81 (0.03)	-0.03 (0.03)	17%	0.00 (0.04)	56%	0.00 (0.04)	48%	-0.01 (0.03)	35%	0.00 (0.03)	52%	-0.01 (0.04)	52%	0.00 (0.04)	49%
	SVR	0.82 (0.03)	0.82 (0.03)	0.82 (0.03)	0.82 (0.03)	0.81 (0.03)	0.82 (0.03)	0.82 (0.03)	-0.01 (0.02)	30%	0.00 (0.04)	59%	0.00 (0.02)	54%	-0.01 (0.03)	40%	0.00 (0.03)	52%	-0.01 (0.03)	47%	-0.01 (0.03)	47%
	Ridge	0.81 (0.03)	0.81 (0.03)	0.82 (0.03)	0.81 (0.03)	0.81 (0.03)	0.81 (0.03)	0.81 (0.03)	0.00 (0.01)	35%	0.00 (0.02)	72%	0.00 (0.02)	56%	0.00 (0.01)	51%	0.00 (0.02)	70%	0.00 (0.01)	58%	0.00 (0.02)	59%
	Lasso	0.81 (0.03)	0.81 (0.03)	0.81 (0.03)	0.81 (0.03)	0.81 (0.03)	0.81 (0.03)	0.81 (0.03)	0.00 (0.00)	9%	0.00 (0.01)	27%	0.00 (0.00)	6%	0.00 (0.01)	3%	0.00 (0.01)	11%	0.00 (0.01)	12%	0.00 (0.01)	8%

Supplementary Table S38. Regression performance (correlation) for VERBAL MEMORY & LANGUAGE component in the concatenation approach in FSet B.

**FSet B**	**Comp 2**	Unimodal			Multimodal			
	**Concat**	FC	SC	GMV	FC+ GMV	SC+ GMV	FC+ SC	FC+SC+GMV
Cond.	Algo.	r	r	r	r	r	r	r
no-deconf.	EN	0.06 (0.02)	0.18 (0.02)	0.12 (0.03)	0.10 (0.03)	0.22 (0.02)	0.14 (0.02)	0.17 (0.05)
	RF	0.06 (0.02)	0.18 (0.01)	0.12 (0.03)	0.13 (0.02)	0.20 (0.04)	0.16 (0.02)	0.18 (0.02)
	SVR	0.09 (0.02)	0.17 (0.02)	0.13 (0.04)	0.12 (0.01)	0.19 (0.01)	0.15 (0.02)	0.18 (0.04)
	Ridge	0.07 (0.02)	0.17 (0.04)	0.14 (0.01)	0.11 (0.04)	0.20 (0.02)	0.17 (0.02)	0.19 (0.02)
	Lasso	0.05 (0.04)	0.08 (0.04)	0.09 (0.02)	0.08 (0.05)	0.13 (0.02)	0.09 (0.07)	0.11 (0.03)
deconf.	EN	-0.06 (0.04)	0.03 (0.03)	0.02 (0.05)	-0.04 (0.03)	0.04 (0.03)	-0.03 (0.03)	-0.02 (0.03)
	RF	-0.06 (0.04)	0.09 (0.02)	0.06 (0.01)	0.01 (0.05)	0.07 (0.02)	0.04 (0.04)	0.06 (0.04)
	SVR	-0.03 (0.02)	0.05 (0.03)	0.04 (0.01)	0.01 (0.02)	0.07 (0.03)	0.02 (0.02)	0.04 (0.02)
	Ridge	-0.06 (0.04)	0.06 (0.04)	0.02 (0.03)	-0.01 (0.02)	0.07 (0.05)	0.00 (0.03)	0.03 (0.03)
	Lasso	-0.02 (0.03)	0.01 (0.02)	-0.02 (0.03)	-0.03 (0.02)	-0.01 (0.02)	-0.02 (0.02)	-0.02 (0.03)

Supplementary Table S39. Regression performance for VERBAL MEMORY & LANGUAGE component in the stacking approach in FSet B.

**FSet B**	**Comp 2**	Unimodal			Multimodal				Unimodal						Multimodal							
	**Stack**	FC	SC	GMV	FC+ GMV	SC+ GMV	FC+ SC	FC+SC +GMV	FC		SC		GMV		FC+ GMV		SC+ GMV		FC+ SC		FC+SC+GMV	
Cond.	Algo.	MAE	MAE	MAE	MAE	MAE	MAE	MAE	R^2	folds > ref.	R^2	folds > ref.	R^2	folds > ref.	R^2	folds > ref.	R^2	folds > ref.	R^2	folds > ref.	R^2	folds > ref.
no-deconf.	EN	0.80 (0.03)	0.80 (0.03)	0.80 (0.03)	0.81 (0.03)	0.81 (0.04)	0.81 (0.04)	0.81 (0.04)	0.00 (0.03)	58%	0.03 (0.04)	82%	0.02 (0.04)	73%	-0.01 (0.05)	50%	0.02 (0.06)	68%	0.01 (0.06)	62%	0.02 (0.06)	66%
	RF	0.81 (0.03)	0.80 (0.03)	0.80 (0.03)	0.82 (0.04)	0.81 (0.04)	0.81 (0.03)	0.81 (0.03)	0.00 (0.04)	50%	0.03 (0.04)	85%	0.01 (0.04)	60%	-0.02 (0.06)	31%	0.00 (0.06)	50%	-0.01 (0.06)	53%	0.00 (0.06)	53%
	SVR	0.80 (0.03)	0.80 (0.03)	0.81 (0.03)	0.82 (0.03)	0.81 (0.04)	0.81 (0.03)	0.81 (0.03)	0.01 (0.03)	60%	0.03 (0.04)	79%	0.01 (0.04)	74%	-0.02 (0.05)	34%	0.01 (0.06)	51%	0.00 (0.06)	55%	0.00 (0.06)	56%
	Ridge	0.80 (0.03)	0.80 (0.03)	0.80 (0.03)	0.82 (0.03)	0.81 (0.04)	0.81 (0.03)	0.81 (0.04)	0.00 (0.02)	65%	0.03 (0.04)	76%	0.02 (0.04)	73%	-0.02 (0.06)	42%	0.01 (0.06)	58%	0.00 (0.05)	55%	0.01 (0.06)	59%
	Lasso	0.80 (0.03)	0.81 (0.03)	0.80 (0.03)	0.81 (0.03)	0.82 (0.03)	0.82 (0.03)	0.82 (0.03)	0.00 (0.02)	61%	0.00 (0.02)	47%	0.01 (0.02)	65%	-0.02 (0.04)	36%	-0.02 (0.04)	40%	-0.02 (0.05)	35%	-0.02 (0.05)	32%
deconf.	EN	0.82 (0.03)	0.82 (0.03)	0.82 (0.03)	0.82 (0.03)	0.82 (0.03)	0.82 (0.03)	0.82 (0.03)	-0.01 (0.02)	10%	0.00 (0.02)	58%	0.00 (0.02)	28%	-0.02 (0.04)	22%	-0.02 (0.03)	23%	-0.02 (0.04)	30%	-0.02 (0.03)	24%
	RF	0.82 (0.03)	0.81 (0.03)	0.81 (0.03)	0.82 (0.03)	0.82 (0.03)	0.82 (0.03)	0.82 (0.03)	-0.03 (0.03)	13%	0.00 (0.04)	58%	0.00 (0.03)	51%	-0.02 (0.05)	38%	-0.02 (0.05)	34%	-0.02 (0.05)	36%	-0.02 (0.04)	38%
	SVR	0.82 (0.03)	0.82 (0.03)	0.82 (0.03)	0.83 (0.03)	0.83 (0.03)	0.82 (0.03)	0.83 (0.03)	-0.01 (0.02)	29%	0.00 (0.04)	59%	0.00 (0.02)	54%	-0.03 (0.04)	16%	-0.03 (0.04)	21%	-0.03 (0.04)	27%	-0.03 (0.04)	26%
	Ridge	0.81 (0.03)	0.81 (0.03)	0.82 (0.03)	0.82 (0.03)	0.82 (0.03)	0.82 (0.03)	0.82 (0.03)	0.00 (0.01)	34%	0.00 (0.02)	73%	0.00 (0.02)	56%	-0.02 (0.04)	32%	-0.03 (0.05)	30%	-0.01 (0.04)	44%	-0.02 (0.04)	42%
	Lasso	0.81 (0.03)	0.81 (0.03)	0.81 (0.03)	0.81 (0.03)	0.82 (0.03)	0.82 (0.03)	0.82 (0.03)	0.00 (0.01)	7%	0.00 (0.01)	27%	0.00 (0.00)	9%	0.00 (0.02)	18%	-0.01 (0.02)	19%	-0.01 (0.03)	18%	-0.01 (0.03)	22%

Supplementary Table S40. Regression performance (correlation) for VERBAL MEMORY & LANGUAGE component in the stacking approach in FSet B.

**FSet B**	**Comp 2**	Unimodal			Multimodal			
	**Stack**	FC	SC	GMV	FC+ GMV	SC+ GMV	FC+ SC	FC+SC+GMV
Cond.	Algo.	r	r	r	r	r	r	r
no-deconf.	EN	0.08 (0.01)	0.19 (0.01)	0.12 (0.03)	0.07 (0.03)	0.15 (0.05)	0.14 (0.04)	0.15 (0.04)
	RF	0.07 (0.04)	0.19 (0.01)	0.11 (0.03)	0.03 (0.03)	0.10 (0.05)	0.11 (0.03)	0.11 (0.03)
	SVR	0.09 (0.02)	0.17 (0.03)	0.11 (0.02)	0.04 (0.08)	0.13 (0.05)	0.12 (0.03)	0.13 (0.05)
	Ridge	0.07 (0.02)	0.17 (0.02)	0.13 (0.04)	0.07 (0.04)	0.14 (0.03)	0.13 (0.03)	0.15 (0.02)
	Lasso	0.06 (0.03)	0.08 (0.03)	0.08 (0.02)	0.01 (0.06)	0.04 (0.04)	0.02 (0.06)	0.02 (0.05)
deconf.	EN	-0.02 (0.03)	0.06 (0.04)	0.02 (0.04)	-0.01 (0.04)	0.02 (0.05)	0.03 (0.05)	0.02 (0.04)
	RF	-0.02 (0.02)	0.11 (0.02)	0.07 (0.03)	0.00 (0.05)	0.02 (0.04)	0.04 (0.04)	0.03 (0.04)
	SVR	0.03 (0.04)	0.09 (0.02)	0.05 (0.02)	-0.04 (0.03)	-0.01 (0.06)	-0.01 (0.03)	-0.02 (0.03)
	Ridge	0.00 (0.04)	0.09 (0.02)	0.04 (0.02)	-0.01 (0.01)	0.01 (0.05)	0.04 (0.02)	0.03 (0.03)
	Lasso	-0.01 (0.02)	0.02 (0.04)	0.00 (0.03)	0.02 (0.04)	0.01 (0.05)	-0.02 (0.07)	0.01 (0.07)

Supplementary Table S41. Regression performance for MEMORY & EXECUTIVE component in the concatenation approach for FSet A with confounders.

**FSet A**	**Comp 1**	EN	RF	SVR	Ridge	Lasso	EN		RF		SVR		Ridge		Lasso		EN	RF	SVR	Ridge	Lasso
Cond.	**Concat**	MAE	MAE	MAE	MAE	MAE	R^2	folds > ref.	R^2	folds > ref.	R^2	folds > ref.	R^2	folds > ref.	R^2	folds > ref.	r	r	r	r	r
nr - eTIV	FC	0.77 (0.03)	0.76 (0.03)	0.76 (0.03)	0.76 (0.03)	0.77 (0.03)	0.04 (0.05)	81%	0.05 (0.06)	79%	0.04 (0.05)	83%	0.04 (0.05)	85%	0.03 (0.04)	76%	0.20 (0.01)	0.22 (0.02)	0.21 (0.01)	0.21 (0.01)	0.17 (0.02)
	SC	0.76 (0.03)	0.77 (0.03)	0.77 (0.03)	0.77 (0.03)	0.77 (0.03)	0.07 (0.05)	89%	0.06 (0.05)	87%	0.05 (0.05)	85%	0.05 (0.04)	85%	0.05 (0.04)	90%	0.26 (0.03)	0.24 (0.03)	0.22 (0.01)	0.22 (0.01)	0.24 (0.02)
	GMV	0.77 (0.03)	0.77 (0.04)	0.77 (0.03)	0.77 (0.04)	0.77 (0.03)	0.05 (0.05)	83%	0.04 (0.06)	76%	0.05 (0.05)	84%	0.05 (0.06)	81%	0.03 (0.04)	79%	0.22 (0.02)	0.21 (0.01)	0.23 (0.01)	0.23 (0.02)	0.17 (0.02)
	CF	0.70 (0.04)	0.71 (0.04)	0.70 (0.04)	0.70 (0.04)	0.71 (0.03)	0.21 (0.07)	99%	0.18 (0.08)	97%	0.21 (0.08)	98%	0.21 (0.08)	98%	0.19 (0.06)	100%	0.46 (0.00)	0.43 (0.01)	0.46 (0.01)	0.46 (0.01)	0.45 (0.01)
	CF+FC	0.70 (0.04)	0.73 (0.04)	0.76 (0.04)	0.76 (0.03)	0.70 (0.03)	0.21 (0.06)	100%	0.14 (0.08)	98%	0.06 (0.06)	86%	0.07 (0.05)	89%	0.20 (0.06)	100%	0.46 (0.01)	0.38 (0.02)	0.26 (0.02)	0.27 (0.01)	0.46 (0.01)
	CF+SC	0.71 (0.04)	0.73 (0.04)	0.76 (0.04)	0.75 (0.04)	0.71 (0.03)	0.19 (0.06)	100%	0.14 (0.09)	94%	0.08 (0.06)	90%	0.10 (0.06)	94%	0.19 (0.06)	100%	0.45 (0.02)	0.37 (0.01)	0.28 (0.01)	0.32 (0.02)	0.45 (0.01)
	CF+ GMV	0.71 (0.04)	0.73 (0.04)	0.76 (0.04)	0.74 (0.04)	0.71 (0.03)	0.19 (0.06)	100%	0.14 (0.08)	94%	0.09 (0.07)	90%	0.11 (0.06)	96%	0.19 (0.06)	100%	0.44 (0.01)	0.38 (0.01)	0.30 (0.02)	0.34 (0.01)	0.45 (0.01)
	CF+FC+ GMV	0.71 (0.04)	0.73 (0.04)	0.75 (0.04)	0.75 (0.04)	0.71 (0.04)	0.20 (0.07)	100%	0.15 (0.08)	95%	0.09 (0.07)	89%	0.08 (0.06)	90%	0.20 (0.06)	100%	0.45 (0.01)	0.39 (0.02)	0.30 (0.02)	0.29 (0.01)	0.46 (0.01)
	CF+SC+ GMV	0.71 (0.04)	0.73 (0.04)	0.75 (0.04)	0.75 (0.04)	0.71 (0.03)	0.19 (0.06)	100%	0.15 (0.09)	95%	0.10 (0.07)	90%	0.10 (0.07)	92%	0.19 (0.06)	100%	0.44 (0.01)	0.38 (0.01)	0.32 (0.01)	0.32 (0.02)	0.45 (0.00)
	CF+FC+ SC	0.70 (0.04)	0.73 (0.04)	0.76 (0.04)	0.75 (0.03)	0.71 (0.04)	0.20 (0.07)	99%	0.15 (0.08)	96%	0.08 (0.06)	91%	0.08 (0.06)	91%	0.20 (0.06)	100%	0.46 (0.01)	0.39 (0.02)	0.28 (0.02)	0.29 (0.02)	0.46 (0.01)
	CF+FC+SC +GMV	0.70 (0.04)	0.72 (0.04)	0.74 (0.04)	0.74 (0.03)	0.71 (0.04)	0.20 (0.07)	100%	0.16 (0.09)	97%	0.11 (0.06)	95%	0.11 (0.06)	96%	0.19 (0.06)	100%	0.45 (0.01)	0.40 (0.01)	0.33 (0.01)	0.34 (0.02)	0.45 (0.01)

Supplementary Table S42. Regression performance for MEMORY & EXECUTIVE component in the stacking approach for FSet A with confounders.

**FSet A**	**Comp 1**	EN	RF	SVR	Ridge	Lasso	EN		RF		SVR		Ridge		Lasso		EN	RF	SVR	Ridge	Lasso
Cond.	**Stack**	MAE	MAE	MAE	MAE	MAE	R^2	folds > ref.	R^2	folds > ref.	R^2	folds > ref.	R^2	folds > ref.	R^2	folds > ref.	r	r	r	r	r
eTIV-nr	FC	0.77 (0.03)	0.76 (0.03)	0.76 (0.03)	0.76 (0.03)	0.77 (0.03)	0.04 (0.04)	82%	0.05 (0.05)	83%	0.05 (0.05)	85%	0.04 (0.04)	85%	0.03 (0.04)	76%	0.21 (0.01)	0.24 (0.01)	0.23 (0.01)	0.23 (0.01)	0.18 (0.02)
	SC	0.77 (0.03)	0.77 (0.03)	0.77 (0.03)	0.77 (0.03)	0.77 (0.03)	0.06 (0.05)	91%	0.06 (0.05)	91%	0.05 (0.05)	86%	0.05 (0.05)	85%	0.05 (0.04)	90%	0.24 (0.02)	0.23 (0.02)	0.23 (0.02)	0.22 (0.02)	0.23 (0.01)
	GMV	0.77 (0.03)	0.77 (0.03)	0.77 (0.03)	0.77 (0.03)	0.77 (0.03)	0.05 (0.05)	85%	0.04 (0.05)	85%	0.05 (0.05)	85%	0.05 (0.06)	80%	0.03 (0.04)	79%	0.28 (0.01)	0.25 (0.02)	0.29 (0.01)	0.28 (0.01)	0.23 (0.01)
	CF	0.70 (0.04)	0.71 (0.04)	0.70 (0.04)	0.70 (0.04)	0.71 (0.03)	0.21 (0.07)	99%	0.18 (0.08)	97%	0.21 (0.08)	98%	0.21 (0.08)	99%	0.19 (0.06)	100%	0.48 (0.00)	0.44 (0.01)	0.48 (0.00)	0.48 (0.00)	0.46 (0.00)
	CF+FC	0.71 (0.05)	0.72 (0.04)	0.71 (0.05)	0.71 (0.05)	0.72 (0.05)	0.18 (0.10)	96%	0.16 (0.08)	96%	0.19 (0.09)	95%	0.19 (0.09)	95%	0.18 (0.09)	97%	0.45 (0.00)	0.41 (0.03)	0.46 (0.01)	0.46 (0.02)	0.43 (0.01)
	CF+SC	0.72 (0.05)	0.73 (0.04)	0.72 (0.05)	0.71 (0.05)	0.72 (0.04)	0.18 (0.09)	96%	0.15 (0.08)	96%	0.18 (0.10)	95%	0.18 (0.09)	96%	0.17 (0.09)	97%	0.44 (0.01)	0.40 (0.02)	0.44 (0.02)	0.44 (0.01)	0.42 (0.02)
	CF+ GMV	0.72 (0.05)	0.73 (0.04)	0.72 (0.04)	0.71 (0.05)	0.72 (0.05)	0.18 (0.09)	95%	0.15 (0.09)	93%	0.17 (0.09)	94%	0.18 (0.10)	95%	0.17 (0.09)	97%	0.44 (0.02)	0.41 (0.01)	0.44 (0.01)	0.45 (0.02)	0.42 (0.01)
	CF+FC+ GMV	0.71 (0.05)	0.72 (0.04)	0.71 (0.05)	0.71 (0.05)	0.71 (0.05)	0.19 (0.09)	95%	0.16 (0.09)	95%	0.19 (0.09)	96%	0.19 (0.09)	95%	0.18 (0.09)	97%	0.45 (0.01)	0.42 (0.02)	0.46 (0.01)	0.46 (0.02	0.43 (0.01)
	CF+SC+ GMV	0.71 (0.04)	0.73 (0.04)	0.72 (0.04)	0.71 (0.05)	0.71 (0.04)	0.18 (0.09)	96%	0.15 (0.09)	94%	0.18 (0.09)	95%	0.18 (0.10)	95%	0.18 (0.09)	97%	0.44 (0.01)	0.40 (0.02)	0.44 (0.01)	0.45 (0.02)	0.42 (0.02)
	CF+FC+ SC	0.71 (0.05)	0.72 (0.04)	0.71 (0.05)	0.71 (0.04)	0.71 (0.04)	0.19 (0.09)	97%	0.16 (0.08)	96%	0.20 (0.10)	94%	0.20 (0.08)	99%	0.18 (0.09)	97%	0.45 (0.00)	0.41 (0.01)	0.46 (0.01)	0.46 (0.02)	0.43 (0.02)
	CF+FC+SC +GMV	0.71 (0.05)	0.72 (0.04)	0.71 (0.05)	0.71 (0.05)	0.71 (0.04)	0.19 (0.09)	96%	0.16 (0.09)	96%	0.19 (0.09)	95%	0.20 (0.09)	95%	0.18 (0.09)	98%	0.45 (0.01)	0.42 (0.01)	0.46 (0.01)	0.46 (0.02)	0.43 (0.01)

Supplementary Table S43. Regression performance for MEMORY & EXECUTIVE component in the concatenation approach for FSet B with confounders

**FSet B**	**Comp 1**	EN	RF	SVR	Ridge	Lasso	EN		RF		SVR		Ridge		Lasso		EN	RF	SVR	Ridge	Lasso
Cond.	**Concat**	MAE	MAE	MAE	MAE	MAE	R^2	folds > ref.	R^2	folds > ref.	R^2	folds > ref.	R^2	folds > ref.	R^2	folds > ref.	r	r	r	r	r
no-deconf.	FC	0.78 (0.03)	0.78 (0.03)	0.77 (0.03)	0.78 (0.03)	0.78 (0.03)	0.02 (0.03)	71%	0.01 (0.04)	61%	0.03 (0.05)	76%	0.00 (0.04)	64%	0.01 (0.03)	72%	0.13 (0.02)	0.12 (0.03)	0.16 (0.01)	0.10 (0.02)	0.12 (0.01)
	SC	0.76 (0.03)	0.77 (0.03	0.78 (0.03)	0.77 (0.04)	0.76 (0.03)	0.07 (0.05)	93%	0.06 (0.06)	84%	0.04 (0.04)	87%	0.05 (0.06)	85%	0.06 (0.04)	93%	0.27 (0.02)	0.24 (0.03)	0.20 (0.02)	0.22 (0.02)	0.27 (0.02)
	GMV	0.77 (0.04)	0.77 (0.04)	0.77 (0.03)	0.77 (0.04)	0.77 (0.03)	0.05 (0.05)	85%	0.04 (0.06)	76%	0.05 (0.05)	85%	0.05 (0.06)	81%	0.03 (0.04)	79%	0.22 (0.01)	0.21 (0.02)	0.23 (0.01)	0.23 (0.02)	0.17 (0.02)
	CF	0.70 (0.04)	0.71 (0.04)	0.70 (0.04)	0.73 (0.04)	0.71 (0.03)	0.21 (0.07)	99%	0.18 (0.09)	97%	0.21 (0.08)	99%	0.21 (0.08)	99%	0.19 (0.06)	100%	0.46 (0.00)	0.43 (0.01)	0.46 (0.00)	0.46 (0.00)	0.45 (0.01)
	CF+FC	0.71 (0.04)	0.73 (0.04)	0.75 (0.04)	0.74 (0.03)	0.71 (0.03)	0.19 (0.06)	100%	0.14 (0.08)	92%	0.08 (0.07)	89%	0.08 (0.05)	95%	0.19 (0.06)	100%	0.44 (0.01)	0.37 (0.01)	0.29 (0.02)	0.29 (0.01)	0.45 (0.01
	CF+SC	0.71 (0.03)	0.73 (0.04)	0.76 (0.04)	0.74 (0.04)	0.71 (0.03)	0.19 (0.06)	100%	0.14 (0.09)	94%	0.08 (0.07)	93%	0.10 (0.06)	96%	0.19 (0.06)	100%	0.44 (0.01)	0.38 (0.01)	0.29 (0.02)	0.32 (0.01)	0.45 (0.01)
	CF+ GMV	0.71 (0.04)	0.73 (0.04)	0.75 (0.04)	0.74 (0.04)	0.71 (0.03)	0.19 (0.06)	100%	0.14 (0.09)	93%	0.09 (0.07)	89%	0.11 (0.06)	96%	0.19 (0.06)	100%	0.44 (0.01)	0.38 (0.01)	0.30 (0.01)	0.34 (0.01)	0.45 (0.01)
	CF+FC+ GMV	0.71 (0.04)	0.73 (0.04)	0.74 (0.04	0.74 (0.04)	0.71 (0.03	0.18 (0.06)	100%	0.14 (0.08)	94%	0.10 (0.07)	91%	0.11 (0.07)	94%	0.19 (0.06)	100%	0.44 (0.01)	0.37 (0.01)	0.31 (0.02)	0.33 (0.01)	0.45 (0.01)
	CF+SC+ GMV	0.71 (0.03)	0.72 (0.04	0.75 (0.04)	0.74 (0.04)	0.71 (0.03)	0.19 (0.06	100%	0.15 (0.09)	95%	0.10 (0.07)	89%	0.10 (0.08)	91%	0.19 (0.06)	100%	0.44 (0.01)	0.39 (0.01)	0.32 (0.01)	0.33 (0.02)	0.45 (0.00)
	CF+FC+ SC	0.71 (0.03)	0.73 (0.04)	0.75 (0.03)	0.74 (0.04)	0.71 (0.03)	0.18 (0.06)	100%	0.14 (0.09)	95%	0.08 (0.06)	91%	0.08 (0.06)	91%	0.19 (0.06)	100%	0.44 (0.01)	0.38 (0.02)	0.29 (0.02)	0.29 (0.01)	0.44 (0.01)
	CF+FC+SC +GMV	0.71 (0.03)	0.72 (0.04)	0.75 (0.04)	0.74 (0.04)	0.71 (0.03)	0.19 (0.06)	100%	0.15 (0.08)	95%	0.10 (0.07)	94%	0.09 (0.08)	91%	0.19 (0.06)	100%	0.44 (0.01)	0.39 (0.01)	0.32 (0.01)	0.31 (0.02)	0.44 (0.01)

Supplementary Table S44. Regression performance for MEMORY & EXECUTIVE component in the stacking approach for FSet B with confounders.

**FSet B**	**Comp 1**	EN	RF	SVR	Ridge	Lasso	EN		RF		SVR		Ridge		Lasso		EN	RF	SVR	Ridge	Lasso
Cond.	**Stack**	MAE	MAE	MAE	MAE	MAE	R^2	folds > ref.	R^2	folds > ref.	R^2	folds > ref.	R^2	folds > ref.	R^2	folds > ref.	r	r	r	r	r
no-deconf.	FC	0.78 (0.03)	0.78 (0.03)	0.77 (0.03)	0.78 (0.03)	0.78 (0.03)	0.02 (0.03)	72%	0.01 (0.04)	61%	0.03 (0.05)	77%	0.00 (0.05)	64%	0.01 (0.03)	71%	0.13 (0.01)	0.13 (0.02)	0.17 (0.01)	0.11 (0.02)	0.12 (0.03)
	SC	0.76 (0.03)	0.77 (0.03)	0.77 (0.03)	0.77 (0.03)	0.76 (0.03)	0.07 (0.05)	91%	0.05 (0.05)	91%	0.04 (0.04)	86%	0.04 (0.05)	85%	0.06 (0.04)	93%	0.26 (0.02	0.22 (0.02)	0.19 (0.01)	0.21 (0.01)	0.25 (0.01)
	GMV	0.77 (0.03)	0.77 (0.03)	0.77 (0.03)	0.77 (0.04)	0.77 (0.03)	0.05 (0.05)	84%	0.04 (0.06)	84%	0.05 (0.05)	86%	0.05 (0.06)	81%	0.03 (0.04)	79%	0.28 (0.02)	0.26 (0.03)	0.29 (0.02)	0.28 (0.02)	0.23 (0.01)
	CF	0.70 (0.04)	0.71 (0.04)	0.70 (0.04)	0.70 (0.04)	0.71 (0.04)	0.21 (0.07)	99%	0.18 (0.09)	97%	0.21 (0.08)	98%	0.21 (0.08)	98%	0.19 (0.06	100%	0.48 (0.00)	0.44 (0.01)	0.48 (0.00)	0.48 (0.00)	0.46 (0.00)
	CF+FC	0.71 (0.05)	0.73 (0.04)	0.71 (0.05)	0.72 (0.05	0.72 (0.04)	0.18 (0.09)	96%	0.15 (0.08)	97%	0.18 (0.09)	96%	0.17 (0.10)	93%	0.16 (0.09)	94%	0.44 (0.01)	0.39 (0.02)	0.45 (0.01)	0.43 (0.02)	0.41 (0.01)
	CF+SC	0.71 (0.04)	0.73 (0.04)	0.71 (0.05)	0.72 (0.05)	0.72 (0.04)	0.18 (0.09)	96%	0.15 (0.09)	95%	0.18 (0.09)	94%	0.17 (0.10)	94%	0.16 (0.09)	95%	0.44 (0.01)	0.40 (0.02)	0.45 (0.01)	0.44 (0.02)	0.41 (0.02)
	CF+ GMV	0.72 (0.05)	0.73 (0.04	0.72 (0.05)	0.72 (0.05)	0.72 (0.05)	0.18 (0.09)	96%	0.15 (0.09)	96%	0.17 (0.09)	96%	0.17 (0.10)	93%	0.17 (0.09)	93%	0.44 (0.02)	0.41 (0.01)	0.44 (0.01)	0.44 (0.01)	0.42 (0.02)
	CF+FC+ GMV	0.72 (0.05)	0.73 (0.04)	0.71 (0.05)	0.72 (0.05)	0.72 (0.05)	0.17 (0.09)	96%	0.15 (0.08)	95%	0.18 (0.09	97%	0.17 (0.10)	95%	0.17 (0.09)	95%	0.44 (0.00)	0.41 (0.01)	0.44 (0.01)	0.44 (0.02)	0.41 (0.02)
	CF+SC+ GMV	0.71 (0.04)	0.73 (0.04)	0.71 (0.05)	0.72 (0.05)	0.72 (0.05)	0.18 (0.09)	96%	0.15 (0.09)	95%	0.18 (0.09)	94%	0.18 (0.10)	94%	0.16 (0.09)	94%	0.44 (0.01)	0.41 (0.01)	0.45 (0.01)	0.44 (0.01)	0.41 (0.01)
	CF+FC+ SC	0.71 (0.04)	0.73 (0.04)	0.71 (0.05)	0.72 (0.05)	0.72 (0.04)	0.18 (0.09)	96%	0.15 (0.09)	95%	0.19 (0.09)	95%	0.17 (0.09)	94%	0.16 (0.09)	94%	0.44 (0.00)	0.40 (0.02)	0.45 (0.01)	0.43 (0.02)	0.41 (0.01)
	CF+FC+SC +GMV	0.72 (0.04)	0.73 (0.04)	0.71 (0.04)	0.72 (0.05)	0.72 (0.04)	0.18 (0.09)	96%	0.16 (0.09)	97%	0.18 (0.09)	95%	0.17 (0.10)	95%	0.16 (0.09)	95%	0.44 (0.01)	0.41 (0.02)	0.45 (0.01)	0.44 (0.02)	0.41 (0.02)

Supplementary Table S45. Regression performance for VERBAL MEMORY & LANGUAGE component in the concatenation approach in FSet A with confounders.

**FSet A**	**Comp 2**	EN	RF	SVR	Ridge	Lasso	EN		RF		SVR		Ridge		Lasso		EN	RF	SVR	Ridge	Lasso
Cond.	**Concat**	MAE	MAE	MAE	MAE	MAE	R^2	folds > ref.	R^2	folds > ref.	R^2	folds > ref.	R^2	folds > ref.	R^2	folds > ref.	r	r	r	r	r
nr - eTIV	FC	0.81 (0.03)	0.82 (0.03)	0.81 (0.03)	0.81 (0.03)	0.81 (0.03)	0.00 (0.02)	50%	-0.02 (0.03)	33%	0.00 (0.03)	57%	0.00 (0.02)	60%	0.00 (0.01)	14%	0.03 (0.03)	0.00 (0.02)	0.08 (0.03)	0.04 (0.03)	-0.01 (0.03)
	SC	0.79 (0.03)	0.79 (0.03)	0.79 (0.03)	0.79 (0.03)	0.79 (0.03)	0.04 (0.05)	81%	0.04 (0.05)	75%	0.04 (0.06)	81%	0.05 (0.04)	85%	0.04 (0.04)	82%	0.21 (0.02)	0.20 (0.03)	0.20 (0.04)	0.22 (0.01)	0.20 (0.02)
	GMV	0.80 (0.03)	0.80 (0.03)	0.81 (0.03)	0.81 (0.03)	0.80 (0.03)	0.02 (0.04)	72%	0.02 (0.04)	69%	0.01 (0.03)	75%	0.02 (0.04)	74%	0.01 (0.02)	60%	0.13 (0.05)	0.13 (0.02)	0.12 (0.02)	0.13 (0.01)	0.09 (0.04)
	CF	0.73 (0.04)	0.74 (0.05)	0.73 (0.05)	0.73 (0.05)	0.74 (0.04)	0.19 (0.08)	98%	0.17 (0.09)	95%	0.19 (0.09)	96%	0.19 (0.09)	98%	0.16 (0.06)	99%	0.44 (0.01)	0.42 (0.01)	0.43 (0.01)	0.44 (0.01)	0.42 (0.01)
	CF+FC	0.75 (0.04)	0.76 (0.04)	0.81 (0.04)	0.80 (0.03)	0.74 (0.04)	0.16 (0.06)	98%	0.12 (0.07)	94%	0.00 (0.06)	62%	0.01 (0.04)	66%	0.16 (0.06)	99%	0.42 (0.01)	0.35 (0.02)	0.11 (0.03)	0.14 (0.01)	0.42 (0.00)
	CF+SC	0.74 (0.04)	0.74 (0.04)	0.79 (0.03)	0.78 (0.03)	0.74 (0.03)	0.18 (0.06)	99%	0.17 (0.06)	100%	0.05 (0.06)	83%	0.06 (0.06)	84%	0.17 (0.05)	99%	0.43 (0.01)	0.41 (0.02)	0.23 (0.02)	0.26 (0.02)	0.43 (0.01)
	CF+ GMV	0.74 (0.04)	0.76 (0.04)	0.79 (0.04)	0.78 (0.03)	0.74 (0.03)	0.17 (0.07)	97%	0.14 (0.07)	97%	0.06 (0.06)	86%	0.09 (0.05)	91%	0.16 (0.06)	99%	0.42 (0.01)	0.38 (0.02)	0.25 (0.03)	0.30 (0.02)	0.43 (0.01)
	CF+FC+ GMV	0.74 (0.04)	0.76 (0.04)	0.80 (0.04)	0.80 (0.04)	0.74 (0.03)	0.16 (0.06)	99%	0.13 (0.07)	96%	0.02 (0.07)	69%	0.03 (0.06)	72%	0.17 (0.06)	99%	0.42 (0.02)	0.36 (0.02)	0.19 (0.03)	0.19 (0.03)	0.43 (0.00)
	CF+SC+ GMV	0.74 (0.04)	0.75 (0.04)	0.79 (0.03)	0.79 (0.03)	0.74 (0.03)	0.18 (0.06)	99%	0.16 (0.07)	99%	0.05 (0.06)	83%	0.06 (0.06)	78%	0.17 (0.06)	99%	0.43 (0.01)	0.40 (0.01)	0.23 (0.01)	0.24 (0.02)	0.43 (0.01)
	CF+FC+ SC	0.74 (0.03)	0.75 (0.04)	0.79 (0.04)	0.79 (0.03)	0.74 (0.03)	0.17 (0.06)	99%	0.15 (0.07)	96%	0.03 (0.07)	69%	0.04 (0.06)	79%	0.17 (0.05)	99%	0.43 (0.01)	0.39 (0.02)	0.20 (0.01)	0.20 (0.02)	0.43 (0.01)
	CF+FC+SC +GMV	0.74 (0.03)	0.76 (0.04)	0.79 (0.04)	0.79 (0.03)	0.74 (0.03)	0.17 (0.06)	99%	0.14 (0.07)	96%	0.03 (0.07)	73%	0.05 (0.05)	82%	0.17 (0.05)	99%	0.43 (0.01)	0.38 (0.02)	0.20 (0.02)	0.22 (0.01)	0.43 (0.01)

Supplementary Table S46. Regression performance for VERBAL MEMORY & LANGUAGE component in the stacking approach in FSet A with confounders.

**FSet A**	**Comp 2**	EN	RF	SVR	Ridge	Lasso	EN		RF		SVR		Ridge		Lasso		EN	RF	SVR	Ridge	Lasso
Cond.	**Stack**	MAE	MAE	MAE	MAE	MAE	R^2	folds > ref.	R^2	folds > ref.	R^2	folds > ref.	R^2	folds > ref.	R^2	folds > ref.	r	r	r	r	r
no-deconf.	FC	0.77 (0.03)	0.76 (0.03)	0.76 (0.03)	0.76 (0.03)	0.77 (0.03)	0.04 (0.04)	82%	0.05 (0.05)	83%	0.05 (0.05)	85%	0.04 (0.04)	85%	0.03 (0.04)	76%	0.21 (0.01)	0.24 (0.01)	0.23 (0.01)	0.23 (0.01)	0.18 (0.02)
	SC	0.77 (0.03)	0.77 (0.03)	0.77 (0.03)	0.77 (0.03)	0.77 (0.03)	0.06 (0.05)	91%	0.06 (0.05)	91%	0.05 (0.05)	86%	0.05 (0.05)	85%	0.05 (0.04)	90%	0.24 (0.02)	0.23 (0.02)	0.23 (0.02)	0.22 (0.02)	0.23 (0.01)
	GMV	0.77 (0.03)	0.77 (0.03)	0.77 (0.04)	0.77 (0.04)	0.77 (0.03)	0.05 (0.05)	85%	0.04 (0.05)	85%	0.05 (0.05)	85%	0.05 (0.06)	80%	0.03 (0.04)	79%	0.28 (0.01)	0.25 (0.02)	0.29 (0.01)	0.28 (0.01)	0.23 (0.01)
	CF	0.70 (0.04)	0.71 (0.04)	0.70 (0.04)	0.70 (0.04)	0.71 (0.04)	0.21 (0.07)	99%	0.18 (0.08)	97%	0.21 (0.08)	98%	0.21 (0.08)	99%	0.19 (0.06)	100%	0.48 (0.00)	0.44 (0.01)	0.48 (0.00)	0.48 (0.00)	0.46 (0.00)
	CF+FC	0.71 (0.05)	0.72 (0.04)	0.71 (0.05)	0.71 (0.05)	0.72 (0.05)	0.18 (0.10)	96%	0.16 (0.08)	96%	0.19 (0.09)	95%	0.19 (0.09)	95%	0.18 (0.09)	97%	0.45 (0.00)	0.41 (0.03)	0.46 (0.01)	0.46 (0.02)	0.43 (0.01)
	CF+SC	0.72 (0.05)	0.73 (0.04)	0.72 (0.05)	0.71 (0.05)	0.72 (0.04)	0.18 (0.09)	96%	0.15 (0.08)	96%	0.18 (0.10)	95%	0.18 (0.09)	96%	0.17 (0.09)	97%	0.44 (0.01)	0.40 (0.02)	0.44 (0.02)	0.44 (0.01)	0.42 (0.02)
	CF+ GMV	0.72 (0.05)	0.73 (0.04)	0.72 (0.04)	0.71 (0.05)	0.72 (0.05)	0.18 (0.09)	95%	0.15 (0.09)	93%	0.17 (0.09)	94%	0.18 (0.10)	95%	0.17 (0.09)	97%	0.44 (0.02)	0.41 (0.01)	0.44 (0.01)	0.45 (0.02)	0.42 (0.01)
	CF+FC+ GMV	0.71 (0.05)	0.72 (0.04)	0.71 (0.05)	0.71 (0.05)	0.71 (0.05)	0.19 (0.09)	95%	0.16 (0.09)	95%	0.19 (0.09)	96%	0.19 (0.09)	95%	0.18 (0.09)	97%	0.45 (0.01)	0.42 (0.02)	0.46 (0.01)	0.46 (0.02)	0.43 (0.01)
	CF+SC+ GMV	0.71 (0.04)	0.73 (0.04)	0.72 (0.04)	0.71 (0.05)	0.71 (0.04)	0.18 (0.09)	96%	0.15 (0.09)	94%	0.18 (0.09)	95%	0.18 (0.10)	95%	0.18 (0.09)	97%	0.44 (0.01)	0.40 (0.02)	0.44 (0.01)	0.45 (0.02)	0.42 (0.02)
	CF+FC+ SC	0.71 (0.05)	0.72 (0.04)	0.71 (0.05)	0.71 (0.04)	0.71 (0.04)	0.19 (0.09)	97%	0.16 (0.08)	96%	0.20 (0.10)	94%	0.20 (0.08)	99%	0.18 (0.09)	97%	0.45 (0.00)	0.41 (0.01)	0.46 (0.01)	0.46 (0.02)	0.43 (0.02)
	CF+FC+SC +GMV	0.71 (0.05)	0.72 (0.04)	0.71 (0.05)	0.71 (0.05)	0.71 (0.04)	0.19 (0.09)	96%	0.16 (0.09)	96%	0.19 (0.09)	95%	0.20 (0.09)	95%	0.18 (0.09)	98%	0.45 (0.01)	0.42 (0.01)	0.46 (0.01)	0.46 (0.02)	0.43 (0.01)

Supplementary Table S47. Regression performance for VERBAL MEMORY & LANGUAGE component in the concatenation approach in FSet B with confounders.

**FSet B**	**Comp 2**	EN	RF	SVR	Ridge	Lasso	EN		RF		SVR		Ridge		Lasso		EN	RF	SVR	Ridge	Lasso
Cond.	**Concat**	MAE	MAE	MAE	MAE	MAE	R^2	folds > ref.	R^2	folds > ref.	R^2	folds > ref.	R^2	folds > ref.	R^2	folds > ref.	r	r	r	r	r
no-deconf.	FC	0.80 (0.03)	0.81 (0.03)	0.80 (0.03)	0.80 (0.03)	0.80 (0.03)	0.00 (0.03)	56%	-0.01 (0.04)	47%	0.01 (0.03)	60%	0.01 (0.02)	66%	0.00 (0.02)	52%	0.06 (0.02)	0.06 (0.02)	0.09 (0.02)	0.07 (0.02)	0.05 (0.04)
	SC	0.80 (0.03)	0.80 (0.03)	0.80 (0.03)	0.80 (0.03)	0.81 (0.03)	0.03 (0.04)	87%	0.03 (0.04)	79%	0.03 (0.04)	79%	0.03 (0.04)	78%	0.01 (0.02)	49%	0.18 (0.02)	0.18 (0.01)	0.17 (0.02)	0.17 (0.04)	0.08 (0.04)
	GMV	0.81 (0.03)	0.81 (0.03)	0.81 (0.03)	0.80 (0.03)	0.80 (0.03)	0.01 (0.04)	68%	0.01 (0.04)	66%	0.02 (0.03)	74%	0.02 (0.04)	72%	0.01 (0.02)	60%	0.12 (0.03)	0.12 (0.03)	0.13 (0.04)	0.14 (0.01)	0.09 (0.02)
	CF	0.73 (0.04)	0.74 (0.05)	0.73 (0.05)	0.73 (0.05)	0.74 (0.04)	0.19 (0.08)	98%	0.17 (0.09)	95%	0.19 (0.09)	97%	0.19 (0.09)	98%	0.16 (0.06)	99%	0.44 (0.01)	0.42 (0.01)	0.43 (0.01)	0.44 (0.01)	0.42 (0.01)
	CF+FC	0.74 (0.04)	0.76 (0.04)	0.80 (0.03)	0.79 (0.03)	0.74 (0.04)	0.15 (0.07)	98%	0.13 (0.07)	98%	0.01 (0.05)	73%	0.04 (0.05)	79%	0.16 (0.06)	99%	0.40 (0.01)	0.36 (0.01)	0.13 (0.04)	0.20 (0.04)	0.42 (0.01)
	CF+SC	0.74 (0.04)	0.74 (0.04)	0.79 (0.04)	0.78 (0.03)	0.74 (0.04)	0.17 (0.07)	99%	0.17 (0.07)	99%	0.06 (0.07)	82%	0.09 (0.05)	95%	0.16 (0.06)	99%	0.42 (0.01)	0.41 (0.03)	0.25 (0.03)	0.30 (0.02)	0.42 (0.01)
	CF+ GMV	0.74 (0.04)	0.75 (0.04	0.79 (0.03)	0.78 (0.03)	0.74 (0.03)	0.17 (0.07)	98%	0.15 (0.07)	98%	0.06 (0.06)	85%	0.08 (0.06)	90%	0.16 (0.06)	99%	0.42 (0.01)	0.38 (0.02)	0.25 (0.02)	0.30 (0.04)	0.43 (0.01)
	CF+FC+ GMV	0.74 (0.04)	0.75 (0.04)	0.79 (0.04)	0.79 (0.04)	0.74 (0.04)	0.16 (0.07)	98%	0.14 (0.07)	98%	0.03 (0.06)	73%	0.04 (0.06)	73%	0.16 (0.06)	99%	0.40 (0.01)	0.38 (0.01)	0.19 (0.02)	0.22 (0.02)	0.42 (0.01)
	CF+SC+ GMV	0.74 (0.04)	0.75 (0.04)	0.78 (0.04)	0.77 (0.04)	0.74 (0.03)	0.17 (0.07)	99%	0.16 (0.07)	99%	0.06 (0.06)	90%	0.10 (0.07)	96%	0.17 (0.06)	99%	0.42 (0.01)	0.40 (0.02)	0.26 (0.01)	0.31 (0.03)	0.43 (0.01)
	CF+FC+ SC	0.74 (0.04)	0.75 (0.04)	0.79 (0.03)	0.79 (0.03	0.74 (0.04)	0.16 (0.07)	98%	0.16 (0.07)	98%	0.03 (0.06)	81%	0.04 (0.06)	83%	0.16 (0.06)	99%	0.40 (0.01)	0.40 (0.01)	0.19 (0.02)	0.20 (0.02)	0.42 (0.01)
	CF+FC+SC +GMV	0.74 (0.04)	0.75 (0.04)	0.79 (0.04)	0.79 (0.04)	0.74 (0.03)	0.16 (0.07)	97%	0.16 (0.07)	98%	0.03 (0.08)	77%	0.05 (0.06)	84%	0.16 (0.06)	99%	0.40 (0.03)	0.40 (0.03)	0.20 (0.01)	0.23 (0.03)	0.42 (0.01)

Supplementary Table S48. Regression performance for VERBAL MEMORY & LANGUAGE component in the stacking approach in FSet B with confounders.

**FSet B**	**Comp 2**	EN	RF	SVR	Ridge	Lasso	EN		RF		SVR		Ridge		Lasso		EN	RF	SVR	Ridge	Lasso
Cond.	**Stack**	MAE	MAE	MAE	MAE	MAE	R^2	folds > ref.	R^2	folds > ref.	R^2	folds > ref.	R^2	folds > ref.	R^2	folds > ref.	r	r	r	r	r
no-deconf.	FC	0.80 (0.03)	0.81 (0.03)	0.80 (0.03)	0.80 (0.03)	0.80 (0.03)	0.00 (0.03)	58%	0.00 (0.04)	50%	0.01 (0.03)	60%	0.00 (0.02)	65%	0.00 (0.02)	61%	0.08 (0.01)	0.07 (0.04)	0.09 (0.02)	0.07 (0.02)	0.06 (0.03)
	SC	0.80 (0.03)	0.80 (0.03)	0.80 (0.03)	0.80 (0.03)	0.81 (0.03)	0.03 (0.04)	82%	0.03 (0.04)	85%	0.03 (0.04)	79%	0.03 (0.04)	76%	0.00 (0.02)	47%	0.19 (0.01)	0.19 (0.01)	0.17 (0.03)	0.17 (0.02)	0.08 (0.03)
	GMV	0.80 (0.03)	0.80 (0.03)	0.81 (0.03)	0.80 (0.03)	0.80 (0.03)	0.02 (0.04)	73%	0.01 (0.04)	60%	0.01 (0.04)	74%	0.02 (0.04)	73%	0.01 (0.02)	65%	0.12 (0.03)	0.11 (0.03)	0.11 (0.02)	0.13 (0.04)	0.08 (0.02)
	CF	0.73 (0.04)	0.74 (0.05)	0.73 (0.05)	0.74 (0.05)	0.74 (0.04)	0.19 (0.08)	98%	0.17 (0.09)	95%	0.19 (0.09)	96%	0.19 (0.09)	98%	0.16 (0.06)	99%	0.45 (0.00)	0.43 (0.01)	0.45 (0.01)	0.45 (0.00)	0.43 (0.01)
	CF+FC	0.74 (0.05)	0.75 (0.05)	0.75 (0.05)	0.75 (0.05)	0.74 (0.05)	0.15 (0.10)	94%	0.13 (0.09)	93%	0.15 (0.10)	93%	0.15 (0.09)	95%	0.15 (0.09)	95%	0.41 (0.01)	0.38 (0.02)	0.40 (0.01)	0.41 (0.01)	0.40 (0.01)
	CF+SC	0.74 (0.05)	0.75 (0.05)	0.74 (0.05)	0.74 (0.05)	0.75 (0.05)	0.16 (0.10)	96%	0.16 (0.09)	95%	0.16 (0.09)	97%	0.17 (0.09)	96%	0.15 (0.09)	96%	0.42 (0.01)	0.41 (0.02)	0.42 (0.01)	0.43 (0.01)	0.40 (0.01)
	CF+ GMV	0.74 (0.05)	0.75 (0.05)	0.74 (0.05)	0.74 (0.05)	0.74 (0.05)	0.16 (0.09)	97%	0.14 (0.08)	97%	0.16 (0.10)	94%	0.17 (0.09)	94%	0.16 (0.09)	95%	0.41 (0.01)	0.38 (0.02)	0.41 (0.02)	0.42 (0.01)	0.40 (0.01)
	CF+FC+ GMV	0.74 (0.05)	0.75 (0.05)	0.75 (0.05)	0.75 (0.05)	0.74 (0.05)	0.16 (0.09)	96%	0.16 (0.10)	92%	0.16 (0.09)	94%	0.16 (0.09)	95%	0.16 (0.09)	94%	0.41 (0.01)	0.38 (0.01)	0.41 (0.01)	0.41 (0.02)	0.40 (0.01)
	CF+SC+ GMV	0.74 (0.05)	0.75 (0.05)	0.74 (0.05)	0.74 (0.05)	0.75 (0.04)	0.17 (0.09)	97%	0.15 (0.09)	95%	0.17 (0.09)	97%	0.17 (0.09)	95%	0.15 (0.09)	95%	0.42 (0.01)	0.40 (0.01)	0.42 (0.02)	0.42 (0.01)	0.40 (0.01)
	CF+FC+ SC	0.74 (0.05)	0.75 (0.05)	0.75 (0.05)	0.75 (0.05)	0.75 (0.05)	0.16 (0.10)	92%	0.15 (0.09)	93%	0.16 (0.09)	96%	0.16 (0.09	95%	0.15 (0.09)	95%	0.41 (0.01)	0.40 (0.01)	0.41 (0.01)	0.42 (0.01)	0.40 (0.01)
	CF+FC+SC +GMV	0.74 (0.05)	0.75 (0.05)	0.74 (0.05)	0.75 (0.05)	0.74 (0.05)	0.17 (0.09)	96%	0.15 (0.09)	94%	0.16 (0.09)	96%	0.17 (0.09)	95%	0.15 (0.09)	95%	0.42 (0.01)	0.39 (0.02)	0.42 (0.01)	0.42 (0.02)	0.40 (0.01)

Supplementary Table S49. Ranking of modality-level feature importance derived from second-level of stacking approach for the combination FC+SC+GMV and CF+FC+SC+GMV.

	**Without extra features**						**With extra features**							
Target	FC		SC		GMV		CF		FC		SC		GMV	
**Global**	1	100%	3	90%	2	90%	4	100%	1	95%	2	70%	3	75%
**Comp 1**	1	70%	2	50%	3	60%	4	100%	1	45%	2	45%	3	55%
**Comp 2**	1	90%	3	100%	2	90%	4	100%	1	70%	3	65%	2	55%

Note. Without extra features: 1 = lowest ranking (least important modality), 2 = second ranking (medium important modality), 3 = highest ranking (most important modality); with extra features: 1 = lowest ranking (least important modality), 2 = second ranking (less important modality), 3 = third ranking (more important modality), 4 = highest ranking (most important modality). Percentage of cases across analytic choices, in which modality scored the specific ranking, indicated next to the ranking.

Validation analyses

Supplementary Table S50. Regression performance for theoretic composite cognition in the concatenation approach in FSet A.

**FSet A**	**Global TH**	Unimodal			Multimodal				Unimodal						Multimodal							
	**Concat**	FC	SC	GMV	FC+ GMV	SC+ GMV	FC+ SC	FC+SC +GMV	FC		SC		GMV		FC+ GMV		SC+ GMV		FC+ SC		FC+SC+GMV	
Cond.	Algo.	MAE	MAE	MAE	MAE	MAE	MAE	MAE	R^2	folds > ref.	R^2	folds > ref.	R^2	folds > ref.	R^2	folds > ref.	R^2	folds > ref.	R^2	folds > ref.	R^2	folds > ref.
no-deconf.	EN	0.78 (0.03)	0.76 (0.04)	0.76 (0.04)	0.75 (0.04)	0.74 (0.04)	0.75 (0.04)	0.74 (0.04)	0.04 (0.06)	78%	0.11 (0.07)	92%	0.08 (0.07)	88%	0.09 (0.06)	93%	0.13 (0.07)	93%	0.11 (0.07)	92%	0.14 (0.07)	96%
	RF	0.78 (0.03)	0.76 (0.04)	0.78 (0.04)	0.76 (0.04)	0.75 (0.04)	0.76 (0.04)	0.75 (0.04)	0.03 (0.05)	73%	0.09 (0.06)	95%	0.06 (0.06)	86%	0.08 (0.05)	92%	0.11 (0.05)	98%	0.09 (0.06)	95%	0.11 (0.05)	98%
	SVR	0.77 (0.04)	0.75 (0.04)	0.78 (0.04)	0.75 (0.04)	0.74 (0.05)	0.75 (0.04)	0.74 (0.04)	0.04 (0.06)	78%	0.10 (0.07)	90%	0.05 (0.07)	82%	0.10 (0.07)	93%	0.13 (0.08)	91%	0.10 (0.07)	95%	0.12 (0.08)	94%
	Ridge	0.78 (0.03)	0.76 (0.05)	0.76 (0.04)	0.76 (0.04)	0.74 (0.05)	0.76 (0.04)	0.74 (0.04)	0.03 (0.06)	75%	0.10 (0.08)	90%	0.09 (0.05)	93%	0.08 (0.07)	89%	0.13 (0.08)	92%	0.10 (0.07)	90%	0.12 (0.08)	93%
	Lasso	0.78 (0.03)	0.76 (0.03)	0.78 (0.03)	0.77 (0.03)	0.75 (0.04)	0.75 (0.03)	0.75 (0.04)	0.03 (0.03)	81%	0.10 (0.05)	96%	0.05 (0.03)	95%	0.06 (0.04)	94%	0.12 (0.05)	99%	0.11 (0.05)	98%	0.12 (0.05)	99%
deconf.	EN	0.79 (0.03)	0.80 (0.03)	0.79 (0.03)	0.79 (0.03)	0.79 (0.03)	0.79 (0.03)	0.79 (0.03)	0.00 (0.02)	59%	0.00 (0.01)	26%	0.01 (0.02)	70%	0.01 (0.04)	69%	0.01 (0.02)	66%	0.00 (0.03)	61%	0.01 (0.03)	65%
	RF	0.80 (0.03)	0.80 (0.03)	0.79 (0.03)	0.79 (0.03)	0.79 (0.03)	0.80 (0.03)	0.79 (0.03)	-0.01 (0.04)	43%	-0.01 (0.04)	48%	0.01 (0.04)	56%	0.00 (0.04)	53%	0.00 (0.04)	60%	-0.01 (0.04)	48%	0.01 (0.04)	58%
	SVR	0.79 (0.03)	0.80 (0.03)	0.80 (0.03)	0.79 (0.04)	0.80 (0.03)	0.79 (0.03)	0.79 (0.03)	0.00 (0.04)	46%	0.00 (0.03)	46%	0.00 (0.03)	65%	0.00 (0.05)	57%	0.00 (0.03)	55%	-0.01 (0.05)	48%	0.00 (0.05)	53%
	Ridge	0.79 (0.03)	0.80 (0.03)	0.79 (0.03)	0.79 (0.03)	0.79 (0.03)	0.79 (0.03)	0.79 (0.03)	0.00 (0.02)	54%	0.00 (0.02)	63%	0.01 (0.03)	75%	0.00 (0.04)	62%	0.01 (0.02)	69%	0.00 (0.03)	64%	0.01 (0.03)	66%
	Lasso	0.80 (0.03)	0.79 (0.03)	0.79 (0.03)	0.79 (0.03)	0.79 (0.03)	0.79 (0.03)	0.79 (0.03)	0.00 (0.01)	6%	0.00 (0.01)	8%	0.00 (0.01)	11%	0.00 (0.01)	7%	0.00 (0.00)	8%	0.00 (0.00)	8%	0.00 (0.00)	8%

Supplementary Table S51. Regression performance (correlation) for theoretic composite cognition in the concatenation approach in FSet A.

**FSet A**	**Global TH**	Unimodal			Multimodal			
	**Concat**	FC	SC	GMV	FC+ GMV	SC+ GMV	FC+ SC	FC+SC+GMV
Cond.	Algo.	r	r	r	r	r	r	r
no-deconf.	EN	0.21 (0.02)	0.33 (0.01)	0.28 (0.02)	0.30 (0.03)	0.37 (0.02)	0.33 (0.01)	0.37 (0.01)
	RF	0.19 (0.02)	0.31 (0.01)	0.25 (0.01)	0.28 (0.01)	0.34 (0.02)	0.31 (0.01)	0.34 (0.02)
	SVR	0.22 (0.02)	0.33 (0.01)	0.26 (0.01)	0.32 (0.01)	0.37 (0.01)	0.33 (0.02)	0.36 (0.02)
	Ridge	0.19 (0.02)	0.33 (0.01)	0.30 (0.01)	0.29 (0.02)	0.36 (0.02)	0.32 (0.02)	0.36 (0.02)
	Lasso	0.18 (0.04)	0.33 (0.01)	0.24 (0.03)	0.26 (0.03)	0.35 (0.01)	0.35 (0.01)	0.36 (0.01)
deconf.	EN	0.04 (0.03)	0.01 (0.05)	0.10 (0.01)	0.08 (0.02)	0.08 (0.01)	0.05 (0.03)	0.09 (0.03)
	RF	0.05 (0.04)	0.05 (0.04)	0.11 (0.04)	0.09 (0.02)	0.08 (0.02)	0.05 (0.04)	0.10 (0.02)
	SVR	0.07 (0.01)	0.04 (0.01)	0.07 (0.04)	0.09 (0.03)	0.09 (0.01)	0.08 (0.01)	0.11 (0.01)
	Ridge	0.05 (0.04)	0.03 (0.03)	0.10 (0.02)	-0.03 (0.06)	0.09 (0.03)	0.05 (0.02)	0.09 (0.02)
	Lasso	-0.03 (0.03)	-0.02 (0.00)	-0.02 (0.03)	0.08 (0.02)	-0.01 (0.00)	-0.01 (0.00)	0.00 (0.00)

Supplementary Table S52. Regression performance for theoretic composite cognition in the stacking approach in FSet A.

**FSet A**	**Global TH**	Unimodal			Multimodal				Unimodal						Multimodal							
	**Stack**	FC	SC	GMV	FC+ GMV	SC+ GMV	FC+ SC	FC+SC +GMV	FC		SC		GMV		FC+ GMV		SC+ GMV		FC+ SC		FC+SC+GMV	
Cond.	Algo.	MAE	MAE	MAE	MAE	MAE	MAE	MAE	R^2	folds > ref.	R^2	folds > ref.	R^2	folds > ref.	R^2	folds > ref.	R^2	folds > ref.	R^2	folds > ref.	R^2	folds > ref.
no-deconf.	EN	0.78 (0.03)	0.76 (0.04)	0.76 (0.03)	0.76 (0.04)	0.75 (0.04)	0.76 (0.04)	0.74 (0.04)	0.04 (0.05)	81%	0.11 (0.07)	94%	0.08 (0.06)	90%	0.07 (0.07)	85%	0.12 (0.07)	92%	0.10 (0.08)	90%	0.12 (0.07)	97%
	RF	0.78 (0.03)	0.76 (0.04)	0.78 (0.04)	0.78 (0.04)	0.77 (0.05)	0.77 (0.04)	0.76 (0.05)	0.04 (0.05)	74%	0.08 (0.06)	92%	0.06 (0.06)	88%	0.05 (0.08)	72%	0.07 (0.08)	84%	0.06 (0.08)	80%	0.08 (0.08)	84%
	SVR	0.77 (0.03)	0.76 (0.04)	0.77 (0.04)	0.76 (0.04)	0.76 (0.05)	0.76 (0.04)	0.75 (0.04)	0.04 (0.06)	76%	0.10 (0.07)	90%	0.06 (0.07)	83%	0.09 (0.08)	83%	0.10 (0.09)	86%	0.09 (0.08)	87%	0.12 (0.09)	89%
	Ridge	0.78 (0.04)	0.76 (0.05)	0.76 (0.04)	0.76 (0.04)	0.75 (0.05)	0.76 (0.04)	0.75 (0.05)	0.03 (0.06)	70%	0.10 (0.08)	90%	0.09 (0.05)	93%	0.08 (0.07)	82%	0.10 (0.08)	88%	0.09 (0.08)	87%	0.12 (0.08)	92%
	Lasso	0.78 (0.03)	0.76 (0.03)	0.78 (0.03)	0.78 (0.04)	0.76 (0.04)	0.76 (0.04)	0.75 (0.04)	0.03 (0.03)	81%	0.10 (0.05)	96%	0.05 (0.03)	95%	0.04 (0.06)	75%	0.10 (0.07)	93%	0.09 (0.07)	89%	0.11 (0.07)	95%
deconf.	EN	0.79 (0.03)	0.80 (0.03)	0.79 (0.03)	0.80 (0.03)	0.80 (0.03)	0.80 (0.03)	0.80 (0.03)	0.00 (0.02)	57%	0.00 (0.01)	16%	0.01 (0.02)	69%	-0.02 (0.05)	35%	0.00 (0.04)	50%	-0.02 (0.04)	28%	-0.01 (0.04)	46%
	RF	0.80 (0.03)	0.80 (0.03)	0.79 (0.03)	0.80 (0.03)	0.80 (0.03)	0.80 (0.03)	0.80 (0.03)	-0.01 (0.04)	42%	-0.01 (0.04)	45%	0.00 (0.05)	54%	-0.03 (0.04)	23%	-0.02 (0.05)	32%	-0.02 (0.04)	28%	-0.02 (0.05)	30%
	SVR	0.79 (0.03)	0.80 (0.03)	0.79 (0.03)	0.80 (0.03)	0.80 (0.03)	0.80 (0.03)	0.80 (0.03)	0.00 (0.04)	46%	-0.01 (0.03)	46%	0.00 (0.02)	66%	-0.01 (0.05)	45%	-0.02 (0.05)	36%	-0.02 (0.04)	31%	-0.01 (0.05)	39%
	Ridge	0.79 (0.03)	0.79 (0.03)	0.79 (0.03)	0.80 (0.03)	0.80 (0.03)	0.81 (0.03)	0.81 (0.03)	0.00 (0.03)	54%	0.00 (0.02)	66%	0.01 (0.03)	76%	-0.02 (0.05)	35%	-0.02 (0.05)	29%	-0.03 (0.05)	28%	-0.03 (0.05)	29%
	Lasso	0.80 (0.03)	0.79 (0.03)	0.80 (0.03)	0.80 (0.03)	0.80 (0.03)	0.80 (0.03)	0.80 (0.03)	0.00 (0.01)	13%	0.00 (0.01)	8%	0.00 (0.01)	13%	0.00 (0.02)	13%	0.00 (0.02)	10%	0.00 (0.01)	8%	-0.01 (0.02)	11%

Supplementary Table S53. Regression performance (correlation) for theoretic composite cognition in the stacking approach in FSet A.

**FSet A**	**Global TH**	Unimodal			Multimodal			
	**Stack**	FC	SC	GMV	FC+ GMV	SC+ GMV	FC+ SC	FC+SC+GMV
Cond.	Algo.	r	r	r	r	r	r	r
no-deconf.	EN	0.21 (0.03)	0.33 (0.02)	0.30 (0.01)	0.30 (0.03)	0.34 (0.04)	0.32 (0.01)	0.37 (0.02)
	RF	0.20 (0.01)	0.29 (0.01)	0.27 (0.01)	0.25 (0.04)	0.28 (0.05)	0.27 (0.03)	0.31 (0.02)
	SVR	0.23 (0.02)	0.33 (0.01)	0.28 (0.01)	0.32 (0.03)	0.33 (0.02)	0.32 (0.02)	0.36 (0.01)
	Ridge	0.19 (0.03)	0.33 (0.01)	0.32 (0.01)	0.31 (0.03)	0.32 (0.04)	0.31 (0.02)	0.36 (0.04)
	Lasso	0.18 (0.03)	0.33 (0.01)	0.27 (0.03)	0.23 (0.02)	0.31 (0.02)	0.31 (0.02)	0.34 (0.02)
deconf.	EN	0.15 (0.03)	0.08 (0.05)	0.19 (0.03)	0.12 (0.03)	0.14 (0.03)	0.06 (0.07)	0.15 (0.02)
	RF	0.14 (0.01)	0.14 (0.02)	0.16 (0.02)	0.08 (0.03)	0.11 (0.03)	0.07 (0.03)	0.11 (0.02)
	SVR	0.18 (0.02)	0.17 (0.03)	0.20 (0.04)	0.13 (0.04)	0.07 (0.07)	0.05 (0.05)	0.11 (0.08)
	Ridge	0.13 (0.02)	0.19 (0.04)	0.18 (0.03)	0.11 (0.05)	0.10 (0.06)	0.02 (0.03)	0.10 (0.06)
	Lasso	0.00 (0.06)	-0.01 (0.06)	0.04 (0.04)	0.01 (0.03)	0.01 (0.05)	0.01 (0.05)	0.01 (0.06)

Supplementary Table S54. Regression performance for theoretic composite cognition in the concatenation approach in FSet B.

**FSet B**	**Global TH**	Unimodal			Multimodal				Unimodal						Multimodal							
	**Concat**	FC	SC	GMV	FC+ GMV	SC+ GMV	FC+ SC	FC+SC +GMV	FC		SC		GMV		FC+ GMV		SC+ GMV		FC+ SC		FC+SC+GMV	
Cond.	Algo.	MAE	MAE	MAE	MAE	MAE	MAE	MAE	R^2	folds > ref.	R^2	folds > ref.	R^2	folds > ref.	R^2	folds > ref.	R^2	folds > ref.	R^2	folds > ref.	R^2	folds > ref.
no-deconf.	EN	0.79 (0.03)	0.76 (0.04)	0.76 (0.04)	0.76 (0.04)	0.75 (0.04)	0.76 (0.04)	0.75 (0.05)	0.02 (0.05)	73%	0.11 (0.05)	99%	0.08 (0.06)	87%	0.09 (0.07)	87%	0.13 (0.06)	97%	0.10 (0.06)	93%	0.13 (0.07)	96%
	RF	0.79 (0.04)	0.77 (0.04)	0.77 (0.04)	0.77 (0.04)	0.76 (0.04)	0.77 (0.04)	0.76 (0.04)	0.02 (0.05)	62%	0.09 (0.06)	94%	0.06 (0.06)	86%	0.07 (0.05)	91%	0.11 (0.05)	96%	0.09 (0.06)	93%	0.11 (0.05)	97%
	SVR	0.79 (0.03)	0.77 (0.04)	0.77 (0.04)	0.76 (0.04)	0.75 (0.05)	0.77 (0.04)	0.75 (0.04)	0.03 (0.05)	75%	0.08 (0.05)	92%	0.06 (0.07)	83%	0.10 (0.07)	91%	0.11 (0.08)	92%	0.08 (0.06)	89%	0.11 (0.08)	92%
	Ridge	0.79 (0.03)	0.76 (0.04)	0.76 (0.04)	0.75 (0.04)	0.75 (0.04)	0.77 (0.04)	0.75 (0.05)	0.02 (0.05)	67%	0.10 (0.06)	94%	0.09 (0.05)	93%	0.10 (0.07)	91%	0.14 (0.07)	97%	0.07 (0.07)	89%	0.12 (0.08)	94%
	Lasso	0.79 (0.03)	0.77 (0.03)	0.78 (0.03)	0.77 (0.03)	0.76 (0.04)	0.76 (0.03)	0.75 (0.04)	0.02 (0.03)	74%	0.10 (0.04)	99%	0.05 (0.03)	95%	0.06 (0.04)	91%	0.11 (0.04)	100%	0.10 (0.04)	100%	0.12 (0.04)	98%
deconf.	EN	0.80 (0.03)	0.80 (0.03)	0.79 (0.03)	0.79 (0.03)	0.79 (0.03)	0.80 (0.03)	0.79 (0.03)	0.00 (0.01)	51%	0.00 (0.01)	44%	0.01 (0.02)	76%	0.00 (0.03)	58%	0.01 (0.02)	73%	0.00 (0.01)	51%	0.00 (0.02)	65%
	RF	0.80 (0.03)	0.79 (0.03)	0.79 (0.03)	0.79 (0.03)	0.79 (0.03)	0.80 (0.03)	0.79 (0.03)	-0.01 (0.04)	40%	0.01 (0.03)	63%	0.01 (0.04)	56%	0.00 (0.04)	47%	0.00 (0.04)	60%	-0.01 (0.04)	50%	0.00 (0.03)	53%
	SVR	0.79 (0.03)	0.80 (0.03)	0.80 (0.03)	0.79 (0.03)	0.80 (0.04)	0.80 (0.03)	0.79 (0.03)	-0.01 (0.02)	35%	0.00 (0.02)	43%	0.00 (0.03)	65%	0.00 (0.03)	62%	0.00 (0.04)	61%	0.00 (0.02)	44%	0.01 (0.03)	58%
	Ridge	0.80 (0.03)	0.79 (0.03)	0.79 (0.03)	0.79 (0.03)	0.79 (0.03)	0.79 (0.03)	0.79 (0.03)	0.00 (0.01)	51%	0.00 (0.01)	66%	0.01 (0.02)	79%	0.01 (0.02)	63%	0.01 (0.02)	79%	0.00 (0.03)	61%	0.01 (0.02)	73%
	Lasso	0.80 (0.03)	0.80 (0.03)	0.79 (0.03)	0.80 (0.03)	0.80 (0.03)	0.80 (0.03)	0.80 (0.03)	0.00 (0.01)	33%	0.00 (0.01)	15%	0.00 (0.01)	12%	0.00 (0.01)	13%	0.00 (0.01)	9%	0.00 (0.00)	17%	0.00 (0.01)	10%

Supplementary Table S55. Regression performance (correlation) for theoretic composite cognition in the concatenation approach in FSet B.

**FSet B**	**Global TH**	Unimodal			Multimodal			
	**Concat**	FC	SC	GMV	FC+ GMV	SC+ GMV	FC+ SC	FC+SC+GMV
Cond.	Algo.	r	r	r	r	r	r	r
no-deconf.	EN	0.15 (0.04)	0.34 (0.01)	0.28 (0.01)	0.30 (0.03)	0.36 (0.01)	0.32 (0.02)	0.36 (0.02)
	RF	0.16 (0.03)	0.31 (0.02)	0.25 (0.01)	0.26 (0.02)	0.33 (0.02)	0.31 (0.02)	0.33 (0.03)
	SVR	0.18 (0.02)	0.29 (0.01)	0.26 (0.01)	0.32 (0.01)	0.34 (0.02)	0.29 (0.01)	0.34 (0.01)
	Ridge	0.15 (0.03)	0.32 (0.01)	0.30 (0.01)	0.32 (0.02)	0.37 (0.01)	0.28 (0.02)	0.36 (0.01)
	Lasso	0.15 (0.02)	0.33 (0.03)	0.24 (0.03)	0.25 (0.03)	0.35 (0.02)	0.34 (0.02)	0.35 (0.02)
deconf.	EN	0.01 (0.04)	0.02 (0.04)	0.10 (0.03)	0.06 (0.03)	0.10 (0.02)	0.04 (0.07)	0.07 (0.02)
	RF	0.02 (0.02)	0.09 (0.05)	0.11 (0.04)	0.08 (0.03)	0.09 (0.05)	0.05 (0.04)	0.08 (0.04)
	SVR	0.01 (0.02)	0.04 (0.02)	0.08 (0.04)	0.08 (0.03)	0.08 (0.04)	0.04 (0.02)	0.10 (0.01)
	Ridge	-0.02 (0.02)	0.05 (0.03)	0.10 (0.02)	0.08 (0.02)	0.11 (0.02)	0.05 (0.02)	0.09 (0.05)
	Lasso	0.02 (0.05)	0.01 (0.04)	-0.01 (0.02)	-0.01 (0.03)	-0.01 (0.03)	-0.01 (0.00)	0.00 (0.01)

Supplementary Table S56. Regression performance for theoretic composite cognition in the stacking approach in FSet B.

**FSet B**	**Global TH**	Unimodal			Multimodal				Unimodal						Multimodal							
	**Stack**	FC	SC	GMV	FC+ GMV	SC+ GMV	FC+ SC	FC+SC +GMV	FC		SC		GMV		FC+ GMV		SC+ GMV		FC+ SC		FC+SC+GMV	
Cond.	Algo.	MAE	MAE	MAE	MAE	MAE	MAE	MAE	R^2	folds > ref.	R^2	folds > ref.	R^2	folds > ref.	R^2	folds > ref.	R^2	folds > ref.	R^2	folds > ref.	R^2	folds > ref.
no-deconf.	EN	0.79 (0.03)	0.76 (0.04)	0.76 (0.04)	0.77 (0.04)	0.75 (0.05)	0.77 (0.05)	0.75 (0.05)	0.02 (0.05)	73%	0.11 (0.06)	97%	0.08 (0.06)	91%	0.06 (0.07)	80%	0.12 (0.08)	93%	0.09 (0.09)	85%	0.11 (0.08)	89%
	RF	0.79 (0.04)	0.77 (0.04)	0.78 (0.04)	0.78 (0.04)	0.77 (0.04)	0.77 (0.05)	0.76 (0.05)	0.02 (0.04)	69%	0.10 (0.06)	94%	0.06 (0.05)	88%	0.04 (0.06)	74%	0.09 (0.07)	87%	0.07 (0.08)	80%	0.08 (0.07)	86%
	SVR	0.78 (0.03)	0.77 (0.04)	0.78 (0.04)	0.78 (0.04)	0.77 (0.05)	0.78 (0.05)	0.76 (0.05)	0.03 (0.05)	75%	0.08 (0.05)	93%	0.05 (0.07)	83%	0.06 (0.08)	77%	0.08 (0.08)	85%	0.06 (0.08)	79%	0.09 (0.09)	84%
	Ridge	0.79 (0.04)	0.76 (0.04)	0.76 (0.04)	0.77 (0.04)	0.76 (0.05)	0.78 (0.04)	0.76 (0.05)	0.02 (0.05)	71%	0.10 (0.06)	94%	0.09 (0.05)	93%	0.07 (0.07)	85%	0.10 (0.07)	89%	0.06 (0.08)	83%	0.10 (0.08)	88%
	Lasso	0.79 (0.03)	0.77 (0.03)	0.78 (0.03)	0.78 (0.04)	0.76 (0.04)	0.77 (0.04)	0.76 (0.04)	0.02 (0.03)	74%	0.10 (0.04)	99%	0.05 (0.03)	95%	0.04 (0.06)	75%	0.10 (0.08)	92%	0.08 (0.08)	88%	0.10 (0.08)	91%
deconf.	EN	0.80 (0.03)	0.80 (0.03)	0.79 (0.03)	0.80 (0.03)	0.80 (0.03)	0.80 (0.03)	0.80 (0.03)	0.00 (0.02)	53%	0.00 (0.01)	41%	0.01 (0.03)	76%	-0.01 (0.04)	51%	-0.01 (0.04)	46%	-0.02 (0.04)	33%	-0.01 (0.04)	41%
	RF	0.80 (0.03)	0.79 (0.04)	0.79 (0.03)	0.81 (0.03)	0.80 (0.03)	0.81 (0.04)	0.81 (0.03)	-0.02 (0.04)	35%	0.00 (0.04)	60%	0.01 (0.04)	55%	-0.03 (0.04)	26%	-0.02 (0.05)	35%	-0.03 (0.05)	25%	-0.03 (0.05)	30%
	SVR	0.79 (0.03)	0.80 (0.03)	0.79 (0.03)	0.81 (0.04)	0.80 (0.03)	0.81 (0.03)	0.81 (0.04)	-0.01 (0.02)	35%	0.00 (0.02)	43%	0.00 (0.02)	62%	-0.03 (0.05)	34%	-0.02 (0.05)	30%	-0.04 (0.05)	18%	-0.03 (0.05)	27%
	Ridge	0.80 (0.03)	0.79 (0.03)	0.79 (0.03)	0.80 (0.03)	0.80 (0.03)	0.81 (0.04)	0.80 (0.03)	0.00 (0.01)	57%	0.00 (0.01)	65%	0.01 (0.02)	76%	-0.01 (0.04)	49%	-0.01 (0.04)	40%	-0.02 (0.03)	22%	-0.01 (0.04)	37%
	Lasso	0.79 (0.03)	0.80 (0.03)	0.80 (0.03)	0.80 (0.03)	0.80 (0.03)	0.80 (0.03)	0.80 (0.03)	0.00 (0.01)	37%	0.00 (0.01)	13%	0.00 (0.01)	11%	-0.02 (0.04)	19%	-0.01 (0.04)	8%	-0.02 (0.05)	15%	-0.02 (0.04)	14%

Supplementary Table S57. Regression performance (correlation) for theoretic composite cognition in the stacking approach in FSet B.

**FSet B**	**Global TH**	Unimodal			Multimodal			
	**Stack**	FC	SC	GMV	FC+ GMV	SC+ GMV	FC+ SC	FC+SC+GMV
Cond.	Algo.	r	r	r	r	r	r	r
no-deconf.	EN	0.16 (0.03)	0.33 (0.01)	0.31 (0.01)	0.27 (0.04)	0.34 (0.01)	0.31 (0.03)	0.35 (0.03)
	RF	0.16 (0.02)	0.31 (0.02)	0.27 (0.02)	0.23 (0.03)	0.30 (0.03)	0.28 (0.06)	0.31 (0.04)
	SVR	0.18 (0.02)	0.29 (0.02)	0.27 (0.01)	0.27 (0.04)	0.30 (0.04)	0.27 (0.01)	0.32 (0.02)
	Ridge	0.15 (0.01)	0.32 (0.01)	0.32 (0.01)	0.29 (0.01)	0.31 (0.02)	0.26 (0.02)	0.33 (0.02)
	Lasso	0.16 (0.03)	0.33 (0.03)	0.27 (0.03)	0.23 (0.03)	0.31 (0.04)	0.29 (0.02)	0.32 (0.03)
deconf.	EN	0.03 (0.05)	0.10 (0.04)	0.21 (0.02)	0.13 (0.04)	0.14 (0.03)	0.03 (0.05)	0.13 (0.04)
	RF	0.03 (0.02)	0.17 (0.04)	0.17 (0.02)	0.06 (0.02)	0.08 (0.05)	0.04 (0.06)	0.08 (0.03)
	SVR	0.12 (0.03)	0.10 (0.03)	0.19 (0.04)	0.06 (0.05)	0.06 (0.03)	-0.03 (0.02)	0.04 (0.04)
	Ridge	0.07 (0.04)	0.13 (0.05)	0.19 (0.03)	0.11 (0.04)	0.10 (0.05)	-0.01 (0.06)	0.11 (0.02)
	Lasso	0.03 (0.01)	0.02 (0.03)	0.03 (0.05)	0.00 (0.03)	0.02 (0.03)	0.00 (0.06)	0.00 (0.05)

Supplementary Figure S58. Prediction performance of data-driven and theoretically defined global cognition in FSet A in the concatenation approach.

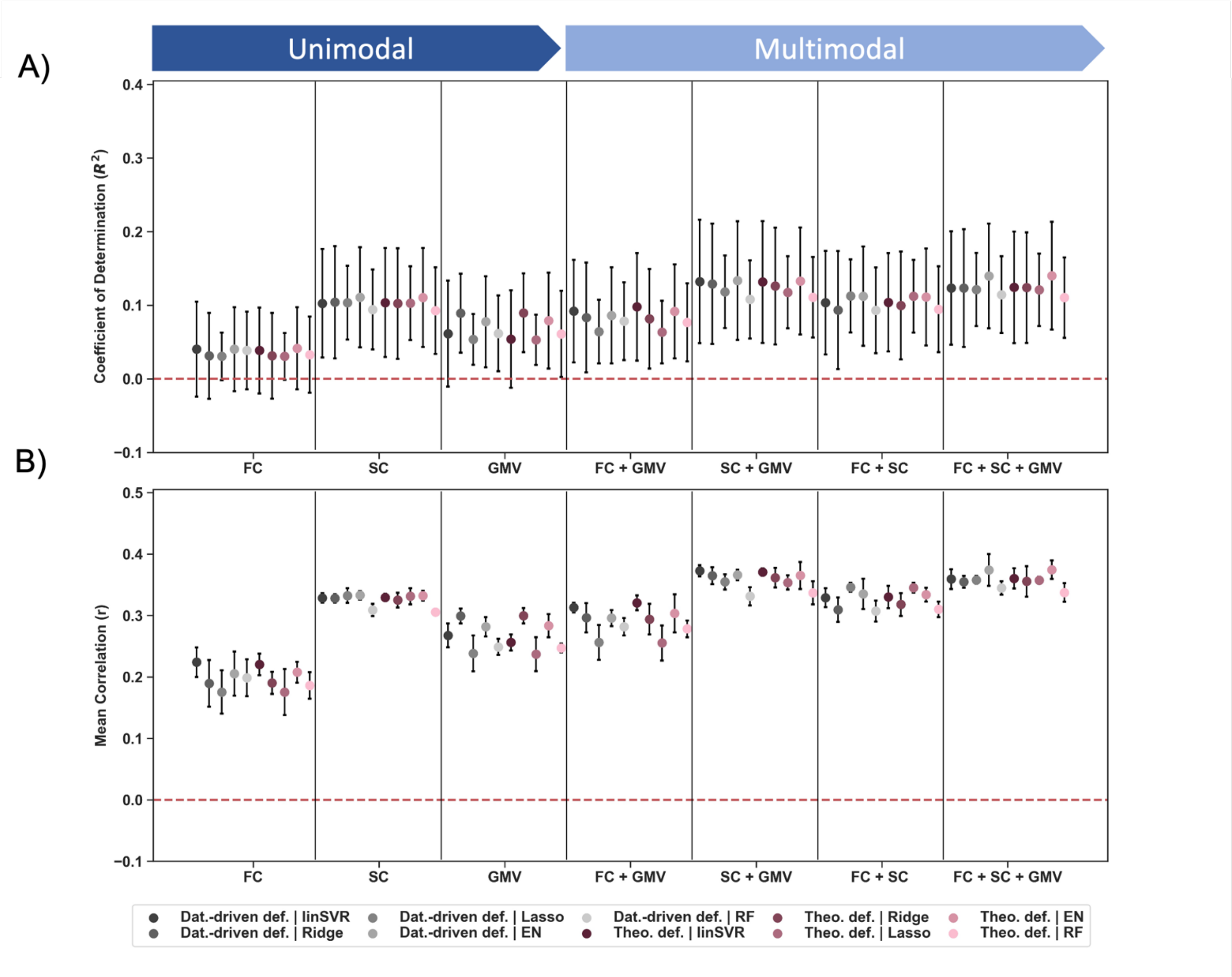

Note. Dat.-driven def. = data-driven defined global cognition, theo. def. = theoretically defined global cognition.

Supplementary Table S59. Results from classification of extreme cognitive groups in FSet A.

**Extreme cognitive**		**Unimodal**						**Multimodal**							
**FSet A**	Algo.	FC	folds > ref.	SC	folds > ref.	GMV	folds > ref.	FC+ GMV	folds > ref.	SC+ GMV	folds > ref.	FC & SC	folds > ref.	FC+SC+ GMV	folds > ref.
**Concat**	Log	49.39% (11.26%)	37%	50.10% (13.39%)	39%	47.48% (12.83%)	24%	47.28% (11.46%)	24%	46.89% (12.91%)	26%	54.06% (13.90%)	52%	49.88% (12.52%)	39%
	RF	55.58% (13.34%)	60%	65.36% (13.39%)	83%	53.58% (14.99%)	51%	56.89% (14.32%)	65%	63.46% (13.89%)	79%	64.55% (13.67%)	82%	64.02% (14.35%)	78%
	SVC	50.10% (12.09%)	42%	52.92% (14.82%)	45%	48.80% (13.15%)	28%	51.45% (15.49%)	43%	47.66% (13.76%)	33%	53.48% (12.98%)	50%	51.72% (14.80%)	47%
	Ridge	51.96% (13.37%)	52%	63.20% (14.44%)	78%	54.45% (14.24%)	49%	55.77% (13.37%)	59%	60.30% (13.34%)	70%	61.73% (13.62%)	74%	61.61% (13.81%)	77%
**Stack**	Log	50.63% (12.09%)	39%	46.74% (11.72%)	29%	49.67% (13.07%)	36%	48.77 % (12.98%)	39%	45.52% (12.21%)	24%	47.90% (13.14%)	42%	46.92% (13.32%)	30%
	RF	56.35% (12.91%)	62%	65.41% (14.57%)	81%	51.95% (15.22%)	50%	52.63% (11.41%)	53%	63.94% (14.14%)	81%	64.51% (14.45%)	79%	60.58% (14.08%)	71%
	SVC	51.02% (13.04%)	42%	53.23% (13.54%)	51%	49.19% (13.39%)	37%	50.17% (14.23%)	38%	49.20% (14.00%)	37%	50.27% (12.40%)	39%	48.5% (12.75%)	33%
	Ridge	52.52% (13.71%)	52%	63.89% (14.02%)	81%	52.60% (14.33%)	46%	52.33% (13.46%)	48%	61.05% (14.29%)	74%	62.34% (14.66%)	74%	59.08% (13.64%)	69%

Note. Mean accuracies (%) displayed with standard deviation (SD) appearing in parentheses.

Supplementary Figure S60. Extreme cognitive group classification results in FSet A.

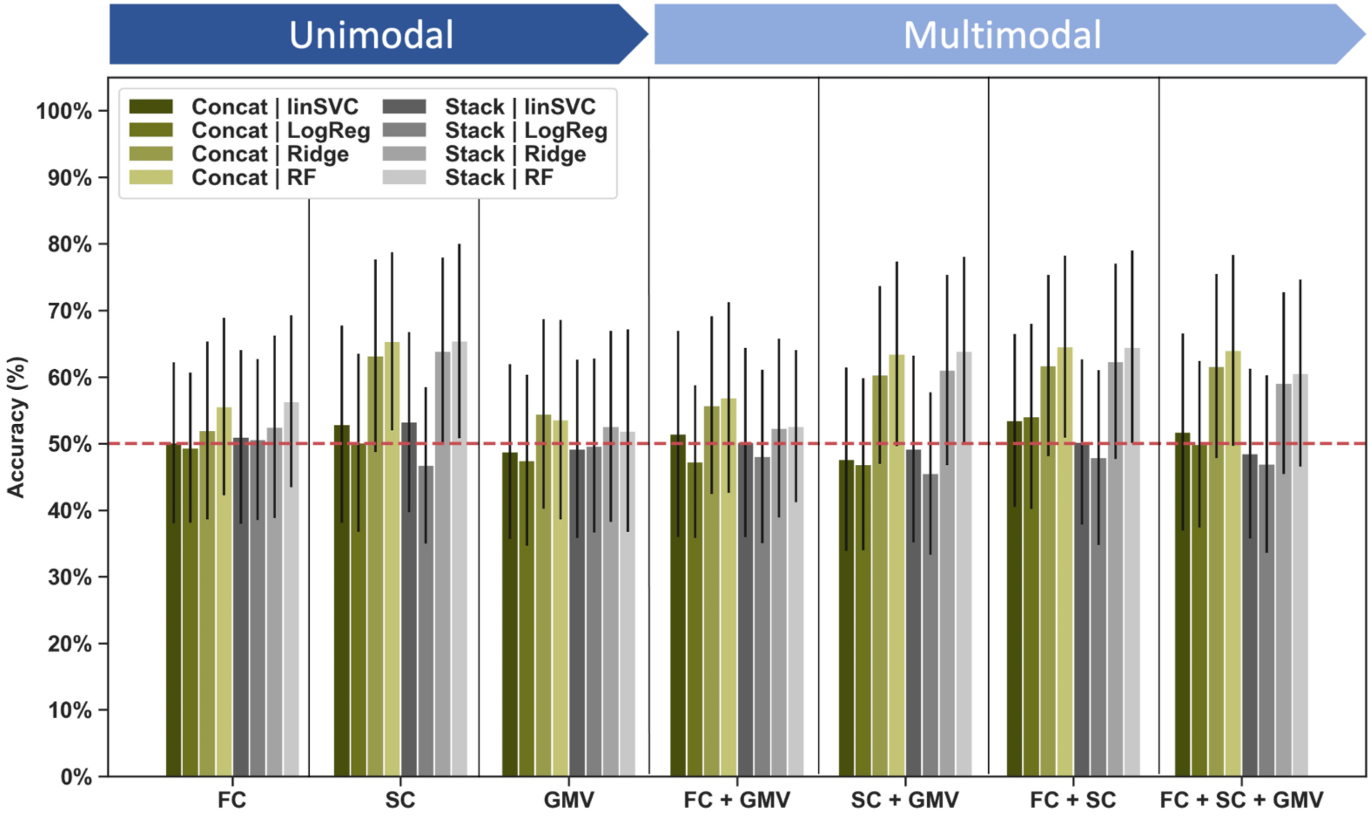

Supplementary Table S61. Regression performance for global cognition in the concatenation approach for FSet C.

**FSet C**	**Global**	Unimodal			Multimodal				Unimodal						Multimodal							
	**Concat**	FC	SC	GMV	FC+ GMV	SC+ GMV	FC+ SC	FC+SC +GMV	FC		SC		GMV		FC+ GMV		SC+ GMV		FC+ SC		FC+SC+GMV	
Cond.	Algo.	MAE	MAE	MAE	MAE	MAE	MAE	MAE	R^2	folds > ref.	R^2	folds > ref.	R^2	folds > ref.	R^2	folds > ref.	R^2	folds > ref.	R^2	folds > ref.	R^2	folds > ref.
no-deconf.	EN	0.77 (0.04)	0.76 (0.04)	0.76 (0.04)	0.76 (0.04)	0.74 (0.04)	0.75 (0.04)	0.74 (0.04)	0.05 (0.06)	79%	0.11 (0.07)	94%	0.08 (0.06)	88%	0.09 (0.07)	90%	0.14 (0.08)	94%	0.11 (0.07)	90%	0.14 (0.07)	98%
	RF	0.77 (0.04)	0.76 (0.04)	0.78 (0.04)	0.76 (0.04)	0.75 (0.07)	0.75 (0.04)	0.75 (0.04)	0.06 (0.05)	92%	0.09 (0.06)	93%	0.06 (0.06)	85%	0.08 (0.05)	93%	0.10 (0.07)	97%	0.10 (0.06)	95%	0.12 (0.05)	98%
	SVR	0.77 (0.04)	0.75 (0.04)	0.77 (0.04)	0.75 (0.04)	0.74 (0.05)	0.75 (0.04)	0.75 (0.04)	0.05 (0.06)	77%	0.10 (0.08)	90%	0.06 (0.07)	86%	0.10 (0.07)	92%	0.13 (0.08)	90%	0.11 (0.08)	91%	0.13 (0.08)	94%
	Ridge	0.77 (0.04)	0.76 (0.05)	0.76 (0.04)	0.76 (0.04)	0.75 (0.05)	0.76 (0.04)	0.74 (0.04)	0.06 (0.06)	85%	0.10 (0.07)	90%	0.09 (0.05)	93%	0.09 (0.07)	91%	0.12 (0.08)	92%	0.10 (0.08)	91%	0.13 (0.08)	94%
	Lasso	0.78 (0.03)	0.76 (0.03)	0.78 (0.03)	0.76 (0.03)	0.75 (0.04)	0.75 (0.03)	0.75 (0.04)	0.05 (0.04)	90%	0.10 (0.05)	96%	0.05 (0.03)	94%	0.07 (0.05)	93%	0.12 (0.05)	99%	0.12 (0.05)	98%	0.12 (0.05)	99%
deconf.	EN	0.79 (0.03)	0.80 (0.03)	0.79 (0.03)	0.79 (0.03)	0.79 (0.03)	0.79 (0.03)	0.79 (0.03)	0.00 (0.04)	49%	0.00 (0.04)	28%	0.01 (0.04)	71%	0.00 (0.04)	64%	0.00 (0.04)	55%	0.00 (0.04)	53%	0.00 (0.04)	59%
	RF	0.79 (0.03)	0.80 (0.03)	0.79 (0.03)	0.79 (0.04)	0.79 (0.03)	0.79 (0.04)	0.79 (0.04)	0.00 (0.04)	58%	-0.01 (0.03)	41%	0.01 (0.02)	54%	0.01 (0.05)	57%	0.00 (0.04)	49%	0.01 (0.05)	57%	0.01 (0.06)	64%
	SVR	0.79 (0.03)	0.80 (0.03)	0.79 (0.03)	0.79 (0.03)	0.79 (0.03)	0.79 (0.03)	0.79 (0.03)	-0.01 (0.02)	43%	0.00 (0.01)	45%	0.00 (0.02)	63%	0.00 (0.05)	53%	0.00 (0.02)	55%	-0.01 (0.03)	40%	0.00 (0.03)	50%
	Ridge	0.79 (0.03)	0.79 (0.03)	0.79 (0.03)	0.79 (0.03)	0.79 (0.03)	0.79 (0.03)	0.79 (0.03)	0.00 (0.02)	59%	0.00 (0.00)	64%	0.01 (0.01)	74%	0.00 (0.02)	71%	0.01 (0.01)	69%	0.00 (0.01)	65%	0.01 (0.01)	70%
	Lasso	0.80 (0.00)	0.79 (0.00)	0.79 (0.00)	0.80 (0.00)	0.79 (0.00)	0.79 (0.00)	0.79 (0.00)	0.00 (0.00)	11%	0.00 (0.00)	6%	0.00 (0.00)	8%	0.00 (0.00)	7%	0.00 (0.00)	5%	0.00 (0.00)	6%	0.00 (0.00)	4%

Supplementary Table S62. Regression performance (correlation) for global cognition in the concatenation approach for FSet C.

**FSet C**	**Global**	Unimodal			Multimodal			
	**Concat**	FC	SC	GMV	FC+ GMV	SC+ GMV	FC+ SC	FC+SC+GMV
Cond.	Algo.	r	r	r	r	r	r	r
no-deconf.	EN	0.23 (0.02)	0.33 (0.01)	0.28 (0.01)	0.30 (0.03)	0.37 (0.01)	0.34 (0.01)	0.38 (0.03)
	RF	0.25 (0.01)	0.30 (0.01)	0.24 (0.01)	0.29 (0.01)	0.35 (0.02)	0.33 (0.00)	0.35 (0.02)
	SVR	0.24 (0.02)	0.33 (0.01)	0.26 (0.02)	0.32 (0.02)	0.37 (0.01)	0.34 (0.02)	0.37 (0.02)
	Ridge	0.24 (0.04)	0.33 (0.00)	0.30 (0.01)	0.30 (0.02)	0.36 (0.01)	0.32 (0.02)	0.36 (0.03)
	Lasso	0.22 (0.03)	0.33 (0.01)	0.24 (0.03)	0.27 (0.03)	0.35 (0.01)	0.35 (0.00)	0.36 (0.01)
deconf.	EN	0.04 (0.05)	0.00 (0.03)	0.11 (0.04)	0.09 (0.04)	0.07 (0.02)	0.05 (0.03)	0.08 (0.02)
	RF	0.08 (0.01)	0.03 (0.02)	0.11 (0.02)	0.10 (0.03)	0.07 (0.01)	0.10 (0.01)	0.11 (0.02)
	SVR	0.06 (0.02)	0.05 (0.02)	0.09 (0.03)	0.11 (0.03)	0.09 (0.03)	0.07 (0.03)	0.10 (0.03)
	Ridge	0.05 (0.03)	0.04 (0.00)	0.11 (0.05)	0.09 (0.04)	0.10 (0.03)	0.05 (0.03)	0.09 (0.00)
	Lasso	-0.02 (0.00)	0.00 (0.00)	-0.02 (0.00)	-0.02 (0.00)	-0.02 (0.00)	-0.02 (0.00)	-0.01 (0.00)

Supplementary Table S63. Regression performance for global cognition in the concatenation approach in FSet C with confounders.

**FSet C**	**Global**	EN	RF	SVR	Ridge	Lasso	EN		RF		SVR		Ridge		Lasso		EN	RF	SVR	Ridge	Lasso
Cond.	**Concat**	MAE	MAE	MAE	MAE	MAE	R^2	folds > ref.	R^2	folds > ref.	R^2	folds > ref.	R^2	folds > ref.	R^2	folds > ref.	r	r	r	r	r
no-deconf.	FC	0.77 (0.04)	0.77 (0.04)	0.77 (0.04)	0.77 (0.04)	0.78 (0.03)	0.05 (0.06)	79%	0.06 (0.05)	92%	0.05 (0.06)	77%	0.06 (0.06)	85%	0.05 (0.04)	90%	0.23 (0.02)	0.25 (0.01)	0.24 (0.02)	0.24 (0.04)	0.22 (0.03)
	SC	0.76 (0.04)	0.76 (0.04)	0.75 (0.04)	0.76 (0.05)	0.76 (0.03)	0.11 (0.07)	94%	0.09 (0.06)	93%	0.10 (0.08)	90%	0.10 (0.07)	90%	0.10 (0.05)	96%	0.33 (0.01)	0.30 (0.01)	0.33 (0.01)	0.33 (0.00)	0.33 (0.01)
	GMV	0.76 (0.04)	0.78 (0.04)	0.77 (0.04)	0.76 (0.04)	0.78 (0.03)	0.08 (0.06)	88%	0.06 (0.06)	85%	0.06 (0.07)	86%	0.09 (0.05)	93%	0.05 (0.03)	94%	0.28 (0.01)	0.24 (0.01)	0.26 (0.02)	0.30 (0.01)	0.24 (0.03)
	CF	0.65 (0.05)	0.65 (0.05)	0.64 (0.05)	0.64 (0.05)	0.65 (0.04)	0.34 (0.08)	100%	0.32 (0.10)	100%	0.34 (0.09)	100%	0.34 (0.09)	100%	0.32 (0.07)	100%	0.58 (0.00)	0.57 (0.01)	0.58 (0.01)	0.58 (0.00)	0.58 (0.00)
	CF+FC	0.65 (0.04)	0.67 (0.05)	0.75 (0.05)	0.74 (0.05)	0.66 (0.04)	0.32 (0.07)	100%	0.29 (0.09)	100%	0.10 (0.09)	87%	0.11 (0.08)	93%	0.32 (0.07)	100%	0.57 (0.01)	0.55 (0.02)	0.32 (0.02)	0.34 (0.03)	0.57 (0.01)
	CF+SC	0.66 (0.04)	0.67 (0.05)	0.73 (0.05)	0.72 (0.05)	0.66 (0.04)	0.32 (0.07)	100%	0.29 (0.08)	100%	0.15 (0.08)	97%	0.18 (0.07)	98%	0.32 (0.07)	100%	0.57 (0.01)	0.54 (0.01)	0.40 (0.00)	0.43 (0.01)	0.58 (0.00)
	CF+ GMV	0.66 (0.04)	0.67 (0.05)	0.70 (0.05)	0.69 (0.05)	0.66 (0.04)	0.32 (0.08)	100%	0.30 (0.08)	100%	0.21 (0.10)	95%	0.24 (0.11)	99%	0.32 (0.07)	100%	0.57 (0.01)	0.55 (0.01)	0.47 (0.01)	0.50 (0.02)	0.58 (0.01)
	CF+FC+ GMV	0.66 (0.04)	0.67 (0.05)	0.73 (0.04)	0.73 (0.04)	0.66 (0.04)	0.31 (0.07)	100%	0.30 (0.08)	100%	0.15 (0.08)	96%	0.16 (0.08)	98%	0.31 (0.07)	100%	0.57 (0.02)	0.55 (0.01)	0.39 (0.01)	0.40 (0.01)	0.57 (0.01)
	CF+SC+ GMV	0.66 (0.04)	0.67 (0.07)	0.72 (0.05)	0.71 (0.05)	0.66 (0.04)	0.31 (0.07)	100%	0.29 (0.09)	100%	0.18 (0.08)	97%	0.20 (0.08)	99%	0.31 (0.07)	100%	0.57 (0.01)	0.56 (0.01)	0.43 (0.01)	0.44 (0.01)	0.57 (0.01)
	CF+FC+ SC	0.66 (0.04)	0.67 (0.05)	0.74 (0.05)	0.73 (0.05)	0.66 (0.04)	0.31 (0.07)	100%	0.29 (0.08)	100%	0.13 (0.08)	94%	0.15 (0.09)	93%	0.31 (0.07)	100%	0.57 (0.00)	0.54 (0.01)	0.37 (0.01)	0.39 (0.02)	0.57 (0.00)
	CF+FC+SC +GMV	0.66 (0.05)	0.67 (0.05)	0.73 (0.05)	0.72 (0.05)	0.66 (0.04)	0.31 (0.07)	100%	0.29 (0.08)	100%	0.15 (0.08)	96%	0.17 (0.09)	97%	0.31 (0.07)	100%	0.56 (0.01)	0.55 (0.02)	0.40 (0.02)	0.42 (0.02)	0.57 (0.00)

Supplementary Figure S64. Prediction of global cognition in FSet C in the concatenation approach.

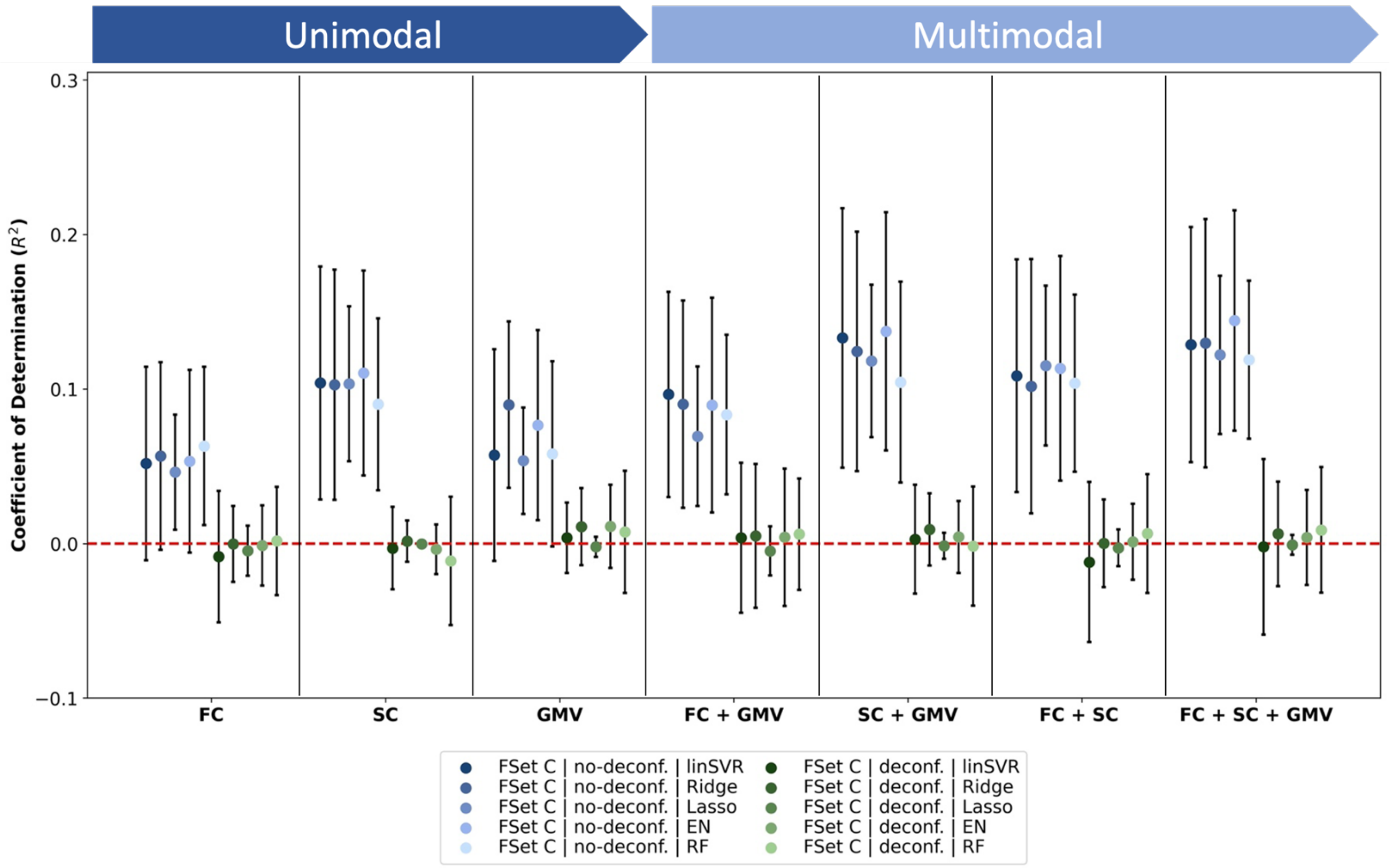

Supplementary Table S65. Age prediction results in the concatenation approach.

**Age**		Unimodal			Multimodal				Unimodal						Multimodal							
**Concat**		FC	SC	GMV	FC+ GMV	SC+ GMV	FC+ SC	FC+SC +GMV	FC		SC		GMV		FC+ GMV		SC+ GMV		FC+ SC		FC+SC+GMV	
Cond.	Algo.	MAE	MAE	MAE	MAE	MAE	MAE	MAE	R^2	folds > ref.	R^2	folds > ref.	R^2	folds > ref.	R^2	folds > ref.	R^2	folds > ref.	R^2	folds > ref.	R^2	folds > ref.
**FSet A**	EN	5.09 (0.40)	4.10 (0.34)	4.63 (0.38)	4.54 (0.41)	3.97 (0.33)	4.10 (0.36)	3.97 (0.34)	0.11 (0.11)	88%	0.42 (0.08)	100%	0.26 (0.10)	99%	0.28 (0.11)	99%	0.46 (0.08)	100%	0.42 (0.09)	100%	0.45 (0.09)	100%
nr	RF	5.42 (0.39)	4.62 (0.37)	5.05 (0.40)	5.10 (0.40)	4.56 (0.35)	4.70 (0.37)	4.61 (0.35)	0.06 (0.08)	83%	0.30 (0.08)	100%	0.15 (0.08)	96%	0.14 (0.09)	96%	0.31 (0.08)	100%	0.27 (0.08)	100%	0.30 (0.08)	100%
	SVR	5.19 (0.41)	4.18 (0.38)	4.69 (0.41)	4.53 (0.40)	4.02 (0.33)	4.21 (0.36)	4.04 (0.35)	0.09 (0.13)	80%	0.40 (0.10)	99%	0.22 (0.12)	96%	0.27 (0.13)	98%	0.43 (0.10)	100%	0.39 (0.10)	100%	0.43 (0.10)	100%
	Ridge	5.18 (0.40)	4.19 (0.38)	4.67 (0.41)	4.57 (0.43)	3.99 (0.33)	4.14 (0.38)	3.96 (0.35)	0.10 (0.12)	85%	0.40 (0.10)	99%	0.23 (0.11)	97%	0.28 (0.12)	97%	0.45 (0.09)	100%	0.41 (0.10)	100%	0.45 (0.10)	100%
	Lasso	5.10 (0.42)	4.23 (0.35)	4.68 (0.40)	4.80 (0.40)	3.98 (0.33)	4.31 (0.36)	4.06 (0.34)	0.10 (0.11)	84%	0.38 (0.08)	100%	0.23 (0.10)	99%	0.19 (0.12)	91%	0.45 (0.09)	100%	0.36 (0.08)	100%	0.42 (0.09)	100%
no-deconf.	EN	0.76 (0.05)	0.63 (0.05)	0.69 (0.04)	0.67 (0.05)	0.60 (0.05)	0.62 (0.05)	0.60 (0.05)	0.13 (0.11)	90%	0.42 (0.09)	100%	0.28 (0.09)	100%	0.31 (0.11)	97%	0.45 (0.09)	100%	0.42 (0.09)	100%	0.46 (0.09)	100%
	RF	0.81 (0.03)	0.69 (0.04)	0.75 (0.03)	0.76 (0.03)	0.68 (0.04)	0.70 (0.04)	0.69 (0.04)	0.09 (0.07)	89%	0.31 (0.08)	100%	0.18 (0.07)	100%	0.18 (0.07)	99%	0.33 (0.08)	100%	0.29 (0.08)	100%	0.32 (0.08)	100%
	SVR	0.78 (0.05)	0.64 (0.05)	0.68 (0.05)	0.69 (0.05)	0.61 (0.05)	0.64 (0.05)	0.61 (0.05)	0.11 (0.12)	86%	0.38 (0.10)	99%	0.28 (0.10)	99%	0.31 (0.11)	96%	0.43 (0.09)	100%	0.39 (0.10)	100%	0.44 (0.10)	100%
	Ridge	0.78 (0.05)	0.63 (0.05)	0.70 (0.05)	0.68 (0.04)	0.60 (0.05)	0.63 (0.05)	0.59 (0.05)	0.12 (0.11)	85%	0.41 (0.09)	99%	0.25 (0.10)	99%	0.31 (0.10)	99%	0.46 (0.09)	100%	0.41 (0.10)	100%	0.47 (0.09)	100%
	Lasso	0.81 (0.03)	0.68 (0.04)	0.75 (0.03)	0.75 (0.03)	0.66 (0.04)	0.68 (0.04)	0.65 (0.04)	0.09 (0.06)	93%	0.34 (0.07)	100%	0.19 (0.05)	100%	0.20 (0.07)	100%	0.37 (0.07)	100%	0.34 (0.07)	100%	0.37 (0.07)	100%
**FSet B**	EN	5.21 (0.38)	4.26 (0.37)	4.62 (0.39)	4.55 (0.37)	4.03 (0.36)	4.33 (0.35)	4.11 (0.34)	0.09 (0.09)	84%	0.37 (0.10)	100%	0.26 (0.10)	99%	0.28 (0.09)	100%	0.43 (0.09)	100%	0.35 (0.09)	100%	0.40 (0.09)	100%
nr	RF	5.43 (0.39)	4.71 (0.38)	5.07 (0.40)	5.09 (0.39)	4.57 (0.39)	4.68 (0.39)	4.57 (0.39)	0.05 (0.08)	78%	0.26 (0.09)	100%	0.15 (0.08)	97%	0.14 (0.08)	96%	0.29 (0.10)	100%	0.27 (0.10)	99%	0.29 (0.11)	99%
	SVR	5.30 (0.39)	4.16 (0.39)	4.70 (0.41)	4.56 (0.37)	4.04 (0.38)	4.41 (0.39)	4.17 (0.37)	0.05 (0.12)	72%	0.37 (0.11)	100%	0.21 (0.13)	97%	0.27 (0.11)	98%	0.41 (0.10)	100%	0.32 (0.11)	100%	0.39 (0.10)	100%
	Ridge	5.28 (0.37)	4.25 (0.37)	4.69 (0.40)	4.64 (0.38)	4.12 (0.38)	4.46 (0.36)	4.18 (0.37)	0.06 (0.11)	81%	0.36 (0.10)	100%	0.23 (0.11)	97%	0.26 (0.11)	99%	0.39 (0.11)	100%	0.30 (0.10)	99%	0.39 (0.09)	100%
	Lasso	5.22 (0.40)	4.38 (0.38)	4.69 (0.39)	4.77 (0.38)	4.10 (0.35)	4.31 (0.37)	4.14 (0.37)	0.07 (0.10)	82%	0.33 (0.10)	100%	0.23 (0.10)	99%	0.20 (0.10)	97%	0.40 (0.09)	100%	0.34 (0.09)	100%	0.39 (0.09)	100%
no-deconf.	EN	0.78 (0.04)	0.64 (0.05)	0.69 (0.04)	0.68 (0.04)	0.61 (0.05)	0.66 (0.05)	0.62 (0.04)	0.11 (0.08)	91%	0.37 (0.09)	100%	0.29 (0.09)	100%	0.31 (0.09)	100%	0.44 (0.09)	100%	0.35 (0.09)	100%	0.42 (0.09)	100%
	RF	0.81 (0.03)	0.71 (0.04)	0.75 (0.03)	0.76 (0.03)	0.68 (0.05)	0.71 (0.04)	0.68 (0.05)	0.07 (0.06)	90%	0.28 (0.09)	100%	0.18 (0.07)	99%	0.17 (0.07)	98%	0.32 (0.10)	100%	0.27 (0.09)	100%	0.31 (0.10)	100%
	SVR	0.78 (0.04)	0.64 (0.05)	0.68 (0.05)	0.69 (0.04)	0.60 (0.05)	0.66 (0.05)	0.63 (0.05)	0.10 (0.10)	83%	0.37 (0.10)	100%	0.28 (0.10)	99%	0.31 (0.09)	98%	0.43 (0.10)	100%	0.33 (0.10)	100%	0.38 (0.10)	100%
	Ridge	0.91 (0.04)	0.91 (0.05)	0.90 (0.05)	0.90 (0.04)	0.89 (0.05)	0.89 (0.04)	0.89 (0.05)	0.09 (0.09)	84%	0.37 (0.10)	100%	0.25 (0.10)	99%	0.30 (0.10)	100%	0.41 (0.10)	100%	0.31 (0.09)	100%	0.40 (0.09)	100%
	Lasso	0.81 (0.02)	0.71 (0.03)	0.75 (0.03)	0.75 (0.03)	0.67 (0.04)	0.71 (0.03)	0.67 (0.04)	0.07 (0.04)	93%	0.27 (0.07)	100%	0.19 (0.05)	100%	0.20 (0.06)	100%	0.34 (0.07)	100%	0.27 (0.07)	100%	0.33 (0.08)	100%

Note. In the no-deconf. condition, the impact of eTIV was controlled for in the target age. Standardized residuals were, then, used for predictions. Resulting MAE values in the nr and no-deconf. condition may, thus, not be easily comparable due to scale differences.

Supplementary Table S66. Age prediction results in the stacking approach.

**Age**		Unimodal			Multimodal				Unidmodal						Multimodal							
**Stack**		FC	SC	GMV	FC+ GMV	SC+ GMV	FC+ SC	FC+SC +GMV	FC		SC		GMV		FC+ GMV		SC+ GMV		FC+ SC		FC+SC+GMV	
Cond.	Algo.	MAE	MAE	MAE	MAE	MAE	MAE	MAE	R^2	folds > ref.	R^2	folds > ref.	R^2	folds > ref.	R^2	folds > ref.	R^2	folds > ref.	R^2	folds > ref.	R^2	folds > ref.
**FSet A**	EN	5.08 (0.40)	4.10 (0.34)	4.62 (0.39)	4.47 (0.40)	3.97 (0.33)	4.04 (0.35)	3.94 (0.33)	0.12 (0.11)	90%	0.42 (0.08)	100%	0.26 (0.10)	99%	0.29 (0.12)	98%	0.44 (0.09)	100%	0.43 (0.10)	100%	0.45 (0.09)	100%
nr	RF	5.42 (0.39)	4.62 (0.37)	5.06 (0.39)	4.89 (0.43)	4.31 (0.40)	4.49 (0.41)	4.29 (0.41)	0.06 (0.08)	86%	0.30 (0.08)	100%	0.15 (0.08)	97%	0.17 (0.12)	91%	0.34 (0.12)	100%	0.30 (0.11)	100%	0.35 (0.12)	99%
	SVR	5.19 (0.39)	4.18 (0.38)	4.69 (0.41)	4.55 (0.41)	4.08 (0.38)	4.16 (0.39)	4.04 (0.37)	0.09 (0.12)	83%	0.40 (0.10)	99%	0.22 (0.13)	96%	0.26 (0.13)	98%	0.41 (0.10)	99%	0.39 (0.11)	99%	0.42 (0.11)	99%
	Ridge	5.18 (0.40)	4.19 (0.35)	4.68 (0.40)	4.52 (0.43)	4.04 (0.35)	4.11 (0.37)	4.02 (0.35)	0.10 (0.12)	85%	0.40 (0.10)	99%	0.22 (0.11)	97%	0.27 (0.12)	96%	0.43 (0.09)	100%	0.41 (0.10)	100%	0.44 (0.09)	100%
	Lasso	5.10 (0.41)	4.23 (0.35)	4.68 (0.39)	4.60 (0.39)	4.07 (0.36)	4.21 (0.36)	4.07 (0.35)	0.10 (0.12)	84%	0.38 (0.09)	100%	0.23 (0.10)	99%	0.25 (0.11)	97%	0.41 (0.10)	100%	0.38 (0.10)	100%	0.41 (0.10)	100%
no-deconf.	EN	0.76 (0.05)	0.63 (0.05)	0.69 (0.04)	0.67 (0.05)	0.60 (0.06)	0.62 (0.06)	0.60 (0.06)	0.13 (0.11)	89%	0.42 (0.09)	100%	0.29 (0.09)	100%	0.30 (0.11)	99%	0.44 (0.09)	100%	0.42 (0.10)	100%	0.45 (0.10)	100%
	RF	0.81 (0.03)	0.69 (0.04)	0.75 (0.03)	0.73 (0.05)	0.65 (0.06)	0.68 (0.06)	0.64 (0.06)	0.08 (0.07)	88%	0.31 (0.08)	100%	0.18 (0.07)	99%	0.20 (0.11)	93%	0.35 (0.12)	100%	0.30 (0.12)	99%	0.36 (0.12)	99%
	SVR	0.77 (0.05)	0.64 (0.05)	0.68 (0.05)	0.67 (0.05)	0.61 (0.06)	0.64 (0.06)	0.61 (0.06)	0.11 (0.12)	85%	0.38 (0.10)	99%	0.28 (0.10)	99%	0.31 (0.11)	98%	0.42 (0.11)	100%	0.39 (0.12)	100%	0.43 (0.11)	100%
	Ridge	0.78 (0.05)	0.63 (0.05)	0.70 (0.05)	0.68 (0.05)	0.61 (0.06)	0.62 (0.06)	0.61 (0.06)	0.11 (0.12)	82%	0.41 (0.10)	99%	0.25 (0.10)	99%	0.29 (0.11)	99%	0.43 (0.10)	100%	0.41 (0.11)	100%	0.44 (0.10)	100%
	Lasso	0.81 (0.03)	0.68 (0.04)	0.75 (0.03)	0.72 (0.05)	0.63 (0.05)	0.66 (0.05)	0.63 (0.05)	0.09 (0.06)	93%	0.34 (0.07)	100%	0.19 (0.05)	100%	0.22 (0.11)	96%	0.39 (0.10)	100%	0.34 (0.10)	100%	0.39 (0.10)	100%
**FSet B**	EN	5.21 (0.38)	4.26 (0.37)	4.62 (0.39)	4.57 (0.40)	4.05 (0.36)	4.25 (0.36)	4.04 (0.35)	0.09 (0.09)	86%	0.37 (0.09)	100%	0.26 (0.10)	99%	0.27 (0.11)	99%	0.41 (0.09)	100%	0.36 (0.10)	100%	0.42 (0.10)	100%
nr	RF	5.43 (0.37)	4.70 (0.37)	5.05 (0.40)	4.87 (0.41)	4.39 (0.40)	4.64 (0.39)	4.36 (0.40)	0.05 (0.07)	79%	0.26 (0.09)	100%	0.15 (0.08)	94%	0.17 (0.13)	91%	0.31 (0.13)	99%	0.26 (0.12)	100%	0.32 (0.13)	99%
	SVR	5.30 (0.39)	4.16 (0.39)	4.70 (0.41)	4.61 (0.44)	4.04 (0.38)	4.19 (0.39)	4.02 (0.38)	0.06 (0.12)	74%	0.37 (0.11)	100%	0.21 (0.13)	97%	0.24 (0.13)	96%	0.41 (0.10)	100%	0.37 (0.11)	100%	0.41 (0.10)	100%
	Ridge	5.28 (0.39)	4.25 (0.37)	4.68 (0.40)	4.60 (0.44)	4.08 (0.40)	4.26 (0.38)	4.08 (0.39)	0.07 (0.11)	77%	0.36 (0.10)	100%	0.23 (0.11)	97%	0.25 (0.11)	98%	0.41 (0.10)	100%	0.36 (0.10)	99%	0.41 (0.10)	100%
	Lasso	5.23 (0.41)	4.38 (0.37)	4.69 (0.39)	4.68 (0.37)	4.11 (0.36)	4.36 (0.38)	4.10 (0.36)	0.07 (0.10)	81%	0.33 (0.10)	100%	0.23 (0.10)	99%	0.23 (0.09)	100%	0.39 (0.09)	100%	0.33 (0.10)	100%	0.40 (0.09)	100%
no-deconf.	EN	0.78 (0.04)	0.64 (0.05)	0.69 (0.04)	0.68 (0.05)	0.61 (0.05)	0.64 (0.05)	0.61 (0.05)	0.10 (0.09)	89%	0.37 (0.09)	100%	0.29 (0.09)	100%	0.30 (0.10)	99%	0.43 (0.09)	100%	0.37 (0.09)	100%	0.43 (0.09)	100%
	RF	0.81 (0.03)	0.71 (0.04)	0.75 (0.03)	0.73 (0.05)	0.65 (0.06)	0.70 (0.05)	0.65 (0.06)	0.07 (0.06)	86%	0.28 (0.08)	100%	0.18 (0.07)	100%	0.19 (0.12)	92%	0.34 (0.12)	100%	0.28 (0.11)	98%	0.34 (0.12)	100%
	SVR	0.78 (0.05)	0.64 (0.05)	0.68 (0.05)	0.68 (0.05)	0.61 (0.05)	0.64 (0.05)	0.61 (0.05)	0.09 (0.10)	84%	0.37 (0.10)	100%	0.28 (0.10)	99%	0.29 (0.11)	99%	0.42 (0.10)	100%	0.37 (0.10)	100%	0.43 (0.10)	100%
	Ridge	0.79 (0.04)	0.64 (0.05)	0.70 (0.05)	0.69 (0.05)	0.61 (0.05)	0.65 (0.05)	0.61 (0.05)	0.09 (0.09)	84%	0.37 (0.10)	100%	0.25 (0.10)	99%	0.27 (0.10)	100%	0.42 (0.10)	100%	0.36 (0.10)	100%	0.42 (0.10)	100%
	Lasso	0.81 (0.02)	0.71 (0.03)	0.75 (0.03)	0.72 (0.04)	0.64 (0.05)	0.69 (0.05)	0.64 (0.05)	0.07 (0.04)	93%	0.27 (0.07)	100%	0.19 (0.05)	100%	0.21 (0.10)	99%	0.36 (0.10)	100%	0.29 (0.10)	100%	0.37 (0.10)	100%

Note. In the no-deconf. condition, the impact of eTIV was controlled for in the target age. Standardized residuals were, then, used for predictions. Resulting MAE values in the nr and no-deconf. condition may, thus, not be easily comparable due to scale differences.

Supplementary Figure S67. Age prediction results in FSet A.

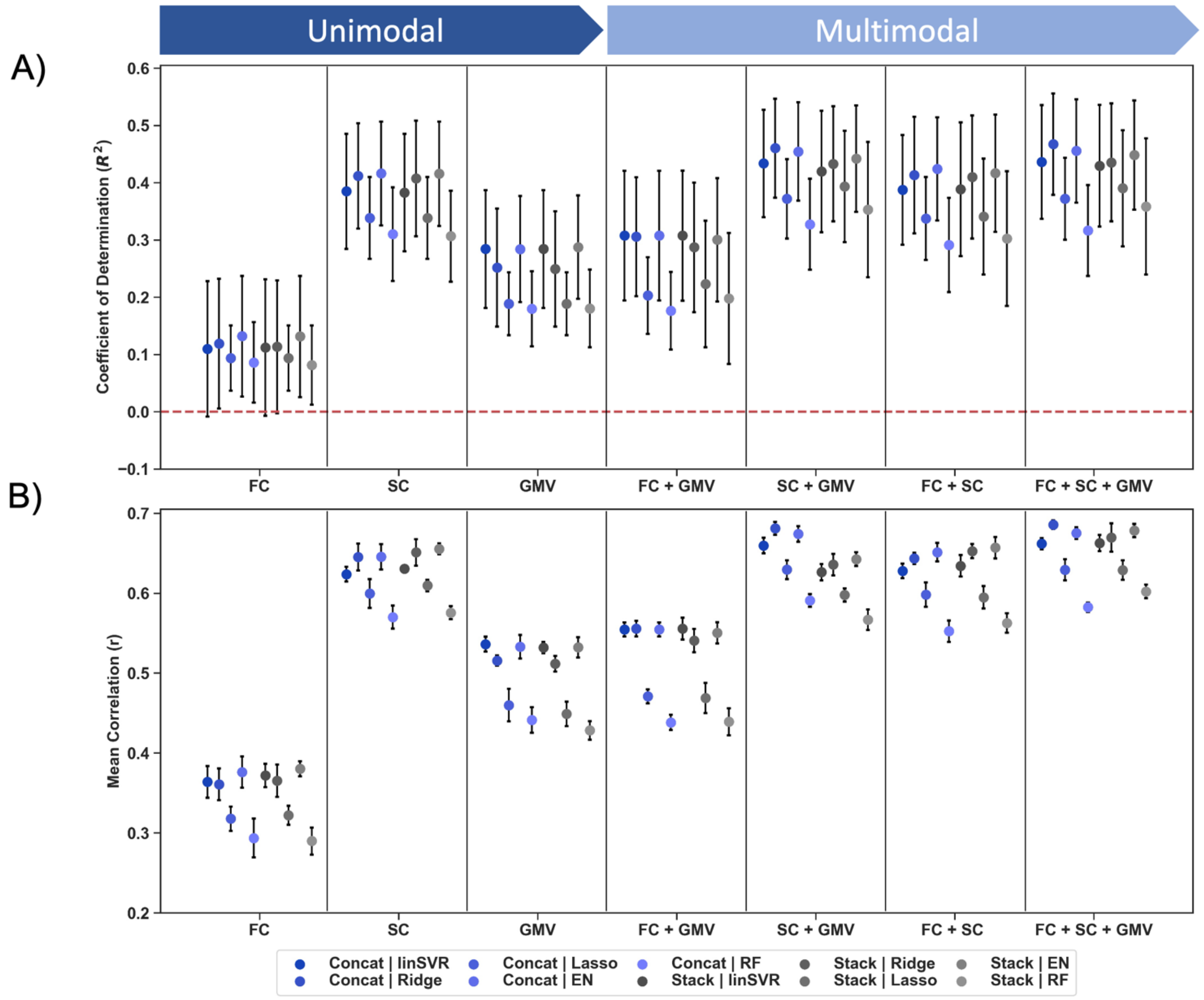

Supplementary Table S68. Sex classification results in FSet A.

**FSet A**	**Sex**	**Unimodal**						**Multimodal**							
App.	Algo.	FC	folds > ref.	SC	folds > ref.	GMV	folds > ref.	FC+ GMV	folds > ref.	SC+ GMV	folds > ref.	FC & SC	folds > ref.	FC+SC +GMV	folds > ref.
**Concat**	Log	60.47% (8.05%)	87%	74.71% (7.05%)	100%	73.50% (7.08%)	100%	74.82% (6.72%)	100%	77.56% (7.89%)	100%	72.50% (7.58%)	100%	75.65% (6.80%)	100%
	RF	63.47% (7.95%)	93%	72.74% (6.54%)	100%	70.44% (8.19%)	99%	70.56% (8.09%)	99%	73.12% (6.76%)	100%	71.38% (8.22%)	100%	74.68% (7.59%)	100%
	SVC	61.53% (7.20%)	91%	74.09% (6.91%)	100%	74.38% (7.24%)	100%	74.56% (6.65%)	100%	76.09% (7.28%)	100%	72.00% (6.40%)	100%	75.21% (7.20%)	100%
	Ridge	65.59% (7.94%)	97%	79.68% (6.49%)	100%	77.59% (6.56%)	100%	79.56% (6.53%)	100%	82.06% (7.16%)	100%	78.47% (7.03%)	100%	82.97% (6.12%)	100%
**Stack**	Log	61.24% (8.35%)	84%	74.44% (6.98%)	100%	74.56% (7.10%)	100%	74.56% (7.10%)	100%	73.59% (7.03%)	100%	74.44% (6.98%)	100%	76.76% (7.03%)	100%
	RF	64.09% (8.56%)	91%	72.32% (6.64%)	100%	69.76% (7.73%)	97%	69.68% (7.63%)	98%	71.03% (7.00%)	99%	72.26% (6.76%)	100%	77.5% (7.12%)	100%
	SVC	60.65% (8.41%)	88%	74.56% (6.61%)	100%	73.82% (7.14%)	100%	73.82% (7.14%)	100%	74.18% (6.25%)	100%	74.56% (6.61%)	100%	76.65% (7.17%)	100%
	Ridge	66.03% (7.70%)	96%	78.68% (6.61%)	100%	77.59% (6.65%)	100%	77.59% (6.65%)	100%	78.24% (6.72%)	100%	78.68% (6.61%)	100%	82.65% (6.29%)	100%

Note. Mean accuracies (AC (%)) displayed with standard deviation (SD) appearing in parentheses.

Supplementary Figure S69. Sex classification results in FSet A.

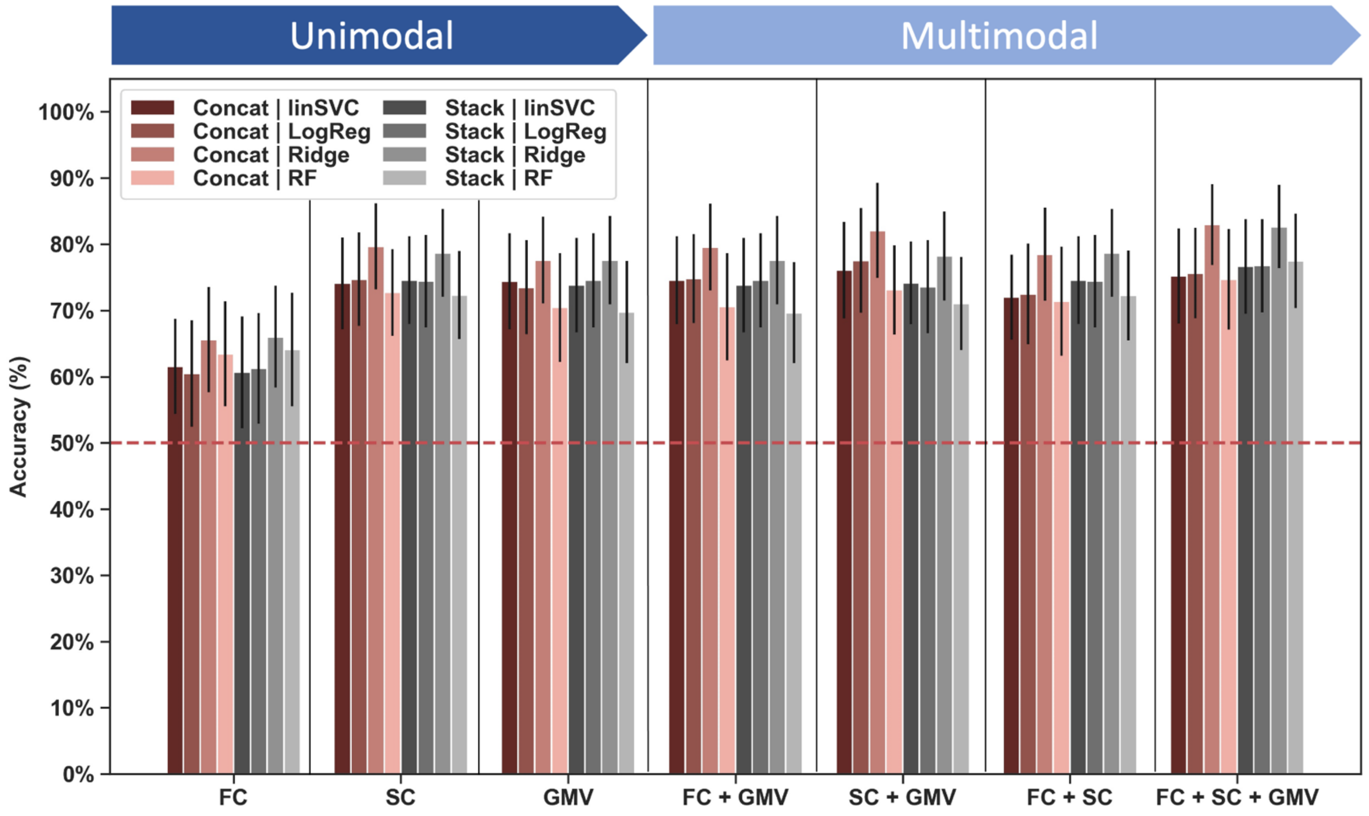

Supplementary Table S70. Educational level prediction results in FSet A.

**FSet A**	**Edu.**	Unimodal			Multimodal				Unimodal						Multimodal							
		FC	SC	GMV	FC+ GMV	SC+ GMV	FC+ SC	FC+SC +GMV	FC		SC		GMV		FC+ GMV		SC+ GMV		FC+ SC		FC+SC+GMV	
Cond.	Algo.	MAE	MAE	MAE	MAE	MAE	MAE	MAE	R^2	folds > ref.	R^2	folds > ref.	R^2	folds > ref.	R^2	folds > ref.	R^2	folds > ref.	R^2	folds > ref.	R^2	folds > ref.
**Concat**	EN	1.74 (0.09)	1.74 (0.10)	1.73 (0.09)	1.69 (0.09)	1.68 (0.09)	1.70 (0.10)	1.67 (0.09)	-0.04 (0.09)	45%	-0.03 (0.10)	50%	-0.01 (0.09)	56%	0.01 (0.09)	69%	0.01 (0.09)	65%	-0.02 (0.11)	50%	0.01 (0.10)	60%
nr	RF	1.75 (0.09)	1.78 (0.07)	1.72 (0.09)	1.71 (0.09)	1.73 (0.08)	1.76 (0.08)	1.72 (0.08)	0.00 (0.06)	69%	-0.01 (0.05)	67%	0.02 (0.08)	66%	0.04 (0.07)	81%	0.02 (0.07)	72%	0.01 (0.06)	72%	0.04 (0.06)	84%
	SVR	1.67 (0.15)	1.73 (0.15)	1.68 (0.14)	1.63 (0.13)	1.67 (0.13)	1.66 (0.14)	1.63 (0.12)	-0.16 (0.16)	17%	-0.37 (0.23)	3%	-0.10 (0.19)	33%	-0.03 (0.13)	46%	-0.08 (0.16)	37%	-0.09 (0.15)	32%	-0.02 (0.12)	54%
	Ridge	1.74 (0.09)	1.74 (0.08)	1.73 (0.09)	1.68 (0.09)	1.70 (0.09)	1.70 (0.10)	1.67 (0.10)	-0.02 (0.12)	60%	-0.01 (0.08)	58%	0.00 (0.09)	59%	0.00 (0.09)	60%	-0.01 (0.09)	60%	0.00 (0.10)	59%	0.00 (0.10)	59%
	Lasso	1.74 (0.08)	1.72 (0.09)	1.72 (0.09)	1.68 (0.09)	1.67 (0.09)	1.68 (0.09)	1.66 (0.09)	-0.01 (0.07)	60%	0.02 (0.07)	73%	0.01 (0.08)	71%	0.01 (0.09)	71%	0.04 (0.09)	79%	0.03 (0.08)	74%	0.04 (0.09)	79%
no-deconf.	EN	0.89 (0.04)	0.89 (0.04)	0.90 (0.03)	0.88 (0.04)	0.88 (0.04)	0.88 (0.04)	0.87 (0.05)	-0.02 (0.07)	47%	-0.01 (0.09)	56%	-0.02 (0.06)	42%	-0.01 (0.07)	47%	0.00 (0.08)	56%	-0.01 (0.09)	50%	0.00 (0.10)	57%
	RF	0.89 (0.03)	0.91 (0.03)	0.89 (0.03)	0.88 (0.03)	0.89 (0.03)	0.90 (0.03)	0.88 (0.04)	0.01 (0.05)	66%	0.01 (0.04)	55%	0.00 (0.05)	45%	0.03 (0.05)	73%	0.01 (0.05)	57%	0.01 (0.04)	66%	-0.01 (0.08)	77%
	SVR	0.85 (0.06)	0.88 (0.05)	0.87 (0.05)	0.85 (0.04)	0.87 (0.05)	0.87 (0.06)	0.86 (0.05)	-0.08 (0.09)	16%	-0.14 (0.07)	3%	-0.08 (0.06)	9%	-0.01 (0.07)	30%	-0.07 (0.08)	15%	-0.07 (0.09)	21%	-0.04 (0.10)	33%
	Ridge	0.88 (0.04)	0.90 (0.04)	0.90 (0.03)	0.88 (0.04)	0.89 (0.04)	0.87 (0.05)	0.87 (0.05)	0.00 (0.06)	54%	-0.01 (0.07)	46%	-0.02 (0.05)	40%	-0.03 (0.08)	39%	-0.03 (0.08)	36%	-0.01 (0.09)	53%	-0.02 (0.09)	43%
	Lasso	0.90 (0.03)	0.91 (0.02)	0.90 (0.02)	0.90 (0.03)	0.90 (0.02)	0.90 (0.03)	0.89 (0.03)	0.02 (0.03)	77%	0.01 (0.02)	67%	0.01 (0.03)	64%	0.02 (0.04)	69%	0.02 (0.03)	71%	0.02 (0.04)	76%	0.02 (0.04)	70%
**Stack**	EN	1.74 (0.09)	1.74 (0.10)	1.73 (0.09)	1.73 (0.10)	1.74 (0.10)	1.77 (0.09)	1.72 (0.09)	-0.04 (0.09)	45%	-0.03 (0.11)	50%	-0.02 (0.10)	53%	-0.04 (0.11)	53%	-0.04 (0.12)	51%	-0.06 (0.11)	48%	-0.02 (0.10)	54%
nr	RF	1.75 (0.09)	1.78 (0.08)	1.72 (0.09)	1.70 (0.11)	1.74 (0.09)	1.76 (0.10)	1.71 (0.10)	0.00 (0.07)	71%	-0.01 (0.05)	63%	0.02 (0.07)	71%	-0.01 (0.10)	56%	-0.03 (0.11)	53%	-0.03 (0.10)	55%	-0.01 (0.11)	63%
	SVR	1.67 (0.16)	1.73 (0.16)	1.69 (0.15)	1.73 (0.09)	1.75 (0.09)	1.76 (0.10)	1.72 (0.09)	-0.17 (0.16)	15%	-0.38 (0.23)	2%	-0.11 (0.19)	33%	-0.02 (0.09)	52%	-0.04 (0.12)	51%	-0.05 (0.12)	45%	-0.01 (0.10)	54%
	Ridge	1.73 (0.09)	1.74 (0.08)	1.72 (0.09)	1.73 (0.10)	1.74 (0.11)	1.74 (0.09)	1.71 (0.10)	-0.01 (0.09)	61%	-0.01 (0.08)	58%	0.00 (0.08)	59%	-0.02 (0.11)	55%	-0.03 (0.11)	48%	-0.04 (0.11)	52%	-0.01 (0.10)	57%
	Lasso	1.74 (0.08)	1.72 (0.09)	1.72 (0.09)	1.72 (0.10)	1.72 (0.10)	1.74 (0.10)	1.70 (0.10)	-0.01 (0.07)	60%	0.02 (0.07)	73%	0.01 (0.08)	71%	-0.01 (0.09)	63%	-0.01 (0.10)	62%	-0.02 (0.11)	62%	0.02 (0.09)	66%
no-deconf.	EN	0.89 (0.04)	0.89 (0.04)	0.90 (0.03)	0.90 (0.04)	0.90 (0.04)	0.91 (0.04)	0.89 (0.04)	-0.02 (0.07)	47%	-0.01 (0.09)	55%	-0.02 (0.06)	44%	-0.02 (0.07)	39%	-0.02 (0.07)	46%	-0.04 (0.10)	43%	-0.02 (0.07)	44%
	RF	0.89 (0.03)	0.91 (0.03)	0.89 (0.03)	0.89 (0.04)	0.91 (0.04)	0.90 (0.04)	0.89 (0.04)	0.02 (0.05)	66%	0.00 (0.04)	56%	0.00 (0.06)	44%	-0.01 (0.07)	47%	-0.04 (0.09)	33%	-0.02 (0.08)	44%	-0.02 (0.08)	46%
	SVR	0.85 (0.06)	0.88 (0.05)	0.87 (0.05)	0.90 (0.04)	0.91 (0.04)	0.90 (0.04)	0.89 (0.04)	-0.08 (0.08)	15%	-0.14 (0.05)	1%	-0.08 (0.06)	5%	-0.03 (0.09)	40%	-0.05 (0.10)	37%	-0.02 (0.09)	50%	-0.02 (0.08)	45%
	Ridge	0.88 (0.04)	0.90 (0.05)	0.90 (0.03)	0.90 (0.04)	0.90 (0.04)	0.90 (0.04)	0.90 (0.04)	0.00 (0.06)	54%	-0.02 (0.11)	46%	-0.02 (0.06)	39%	-0.03 (0.07)	36%	-0.04 (0.09)	37%	-0.04 (0.09)	32%	-0.03 (0.08)	37%
	Lasso	0.90 (0.03)	0.91 (0.02)	0.90 (0.02)	0.89 (0.04)	0.91 (0.03)	0.90 (0.04)	0.89 (0.04)	0.02 (0.03)	77%	0.01 (0.02)	66%	0.01 (0.03)	64%	-0.02 (0.09)	55%	-0.04 (0.10)	37%	-0.02 (0.08)	52%	-0.02 (0.08)	52%

Supplementary Figure S71. Prediction performance of educational level in FSet A.

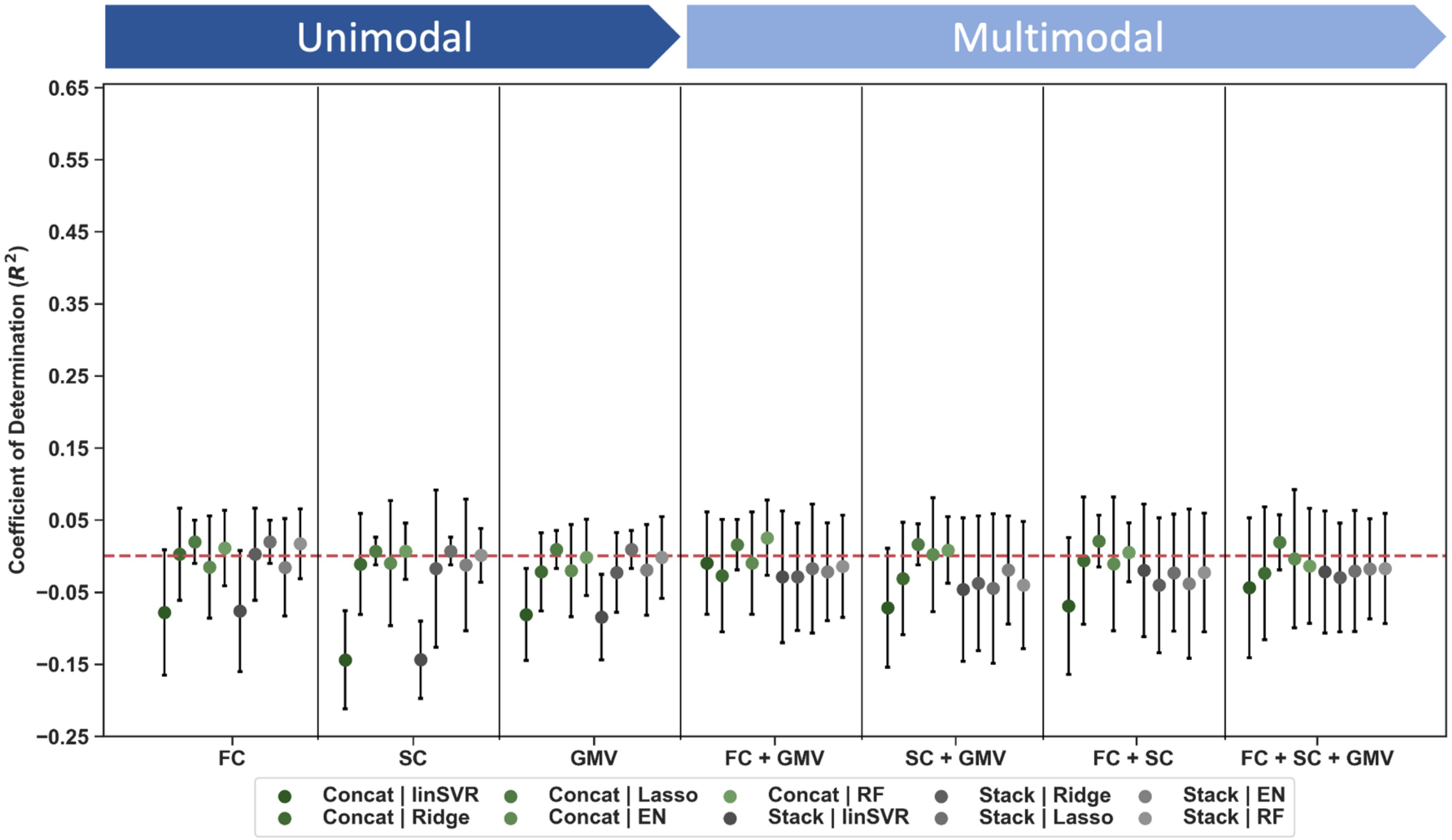

References

Aschenbrenner, S., Tucha, O., & Lange, K. W. (2000). *RWT: Regensburger Wortflüssigkeits-Test*. Hogrefe.

Bäumler, G. (1985). *Farbe-Wort-Interferenztest nach JR Stroop*. Hogrefe, Verlag für Psychologie.

Benton, A., Sivan, A. B., Spreen, O., & Steck, P. (2009). *Der Benton-Test*. Verlag Hans Huber.

Caspers, S., Moebus, S., Lux, S., Pundt, N., Schütz, H., Mühleisen, T. W., Gras, V., Eickhoff, S. B., Romanzetti, S., Stöcker, T., Stirnberg, R., Kirlangic, M. E., Minnerop, M., Pieperhoff, P., Mödder, U., Das, S., Evans, A. C., Jöckel, K.-H., Erbel, R., … Amunts, K. (2014). Studying variability in human brain aging in a population-based German cohort- rationale and design of 1000BRAINS. *Frontiers in Aging Neuroscience*, *6*. https://doi.org/10.3389/fnagi.2014.00149

Della Sala, S., Gray, C., Baddeley, A. D., & Wilson, L. (1997). *The visual patterns test: A test of short-term visual recall.* Thames Valley Test Company.

Gatterer, G. (2008). *Der Alters-Konzentrations-Test* (2nd Edn). Hogrefe.

Jockwitz, C., Caspers, S., Lux, S., Eickhoff, S. B., Jütten, K., Lenzen, S., Moebus, S., Pundt, N., Reid, A., Hoffstaedter, F., Jöckel, K.-H., Erbel, R., Cichon, S., Nöthen, M. M., Shah, N. J., Zilles, K., & Amunts, K. (2017). Influence of age and cognitive performance on resting-state brain networks of older adults in a population-based cohort. *Cortex*, *89*, 28–44. https://doi.org/10.1016/j.cortex.2017.01.008

Lux, S., Hartje, W., Reich, C., & Nagel, C. (2012). *VGT: Verbaler Gedächtnistest: Bielefelder Kategorielle Wortlisten*. Verlag Hans Huber.

Morris, J. C., Heyman, A., Mohs, R. C., Hughes, J. P., van Belle, G., Fillenbaum, G., Mellits, E. D., & Clark, C. (1989). The Consortium to Establish a Registry for Alzheimer's Disease (CERAD). Part I. Clinical and neuropsychological assesment of Alzheimer's disease. *Neurology*, *39*(9), 1159–1159. https://doi.org/10.1212/WNL.39.9.1159

Oswald, W. D., & Fleischmann, U. M. (1997). *Das Nürnberger-Alters-Inventar (NAI)*. Hogrefe.

Regard, M., Strauss, E., & Knapp, P. (1982). Children's Production on Verbal and Non-Verbal Fluency Tasks. *Perceptual and Motor Skills*, *55*(3), 839–844. https://doi.org/10.2466/pms.1982.55.3.839

Schelling, D. (1997). *Block-tapping-test*. Swets Test Service GmbH.

Schmidt, K. H., & Metzler, P. (1992). *Wortschatztest (WST)*. Beltz Test GmbH.

Stroop, J. R. (1935). Studies of interference in serial verbal reactions. *Journal of Experimental Psychology*, *18*(6), 643–662. https://doi.org/10.1037/h0054651

Sturm, W., Willmes, K., & Horn, W. (1993). *Leistungsprüfsystem für 50-90jährige (LPS 50+)*. Hogrefe Verlag für Psychologie.
